# In Vitro Evaluation and Docking Studies of 5-oxo-5*H*-furo[3,2-*g*]chromene-6-carbaldehyde Derivatives as Potential Anti-Alzheimer’s Agents

**DOI:** 10.3390/ijms20215451

**Published:** 2019-11-01

**Authors:** Malose J. Mphahlele, Samantha Gildenhuys, Emmanuel N. Agbo

**Affiliations:** 1 Department of Chemistry, College of Science, Engineering and Technology, University of South Africa, Private Bag X06, Florida 1710, South Africa; 2 Department of Life & Consumer Sciences, College of Agriculture and Environmental Sciences, University of South Africa, Private Bag X06, Florida 1710, South Africa

**Keywords:** 5*H*-furo[3,2-*g*]chromen-5-ones, cholinesterases, β-secretase, lipoxygenases, anti-oxidant, cytotoxicity, molecular docking

## Abstract

A series of novel 2-carbo–substituted 5-oxo-5*H*-furo[3,2-*g*]chromene-6-carbaldehydes and their 6-(4-trifluoromethyl)phenylhydrazono derivatives have been prepared and evaluated for biological activity against the human acetylcholinesterase (AChE) and butyrylcholinesterase (BChE). The most active compounds from each series were, in turn, evaluated against the following enzyme targets involved in Alzheimer’s disease, β-secretase (BACE-1) and lipoxygenase-15 (LOX-15), as well as for anti-oxidant potential. Based on the in vitro results of ChE and β-secretase inhibition, the kinetic studies were conducted to determine the mode of inhibition by these compounds. 2-(4-Methoxyphenyl)-5-oxo-5*H*-furo[3,2-*g*]chromene-6-carbaldehyde (**2f**), which exhibited significant inhibitory effect against all these enzymes was also evaluated for activity against the human lipoxygenase-5 (LOX-5). The experimental results were complemented with molecular docking into the active sites of these enzymes. Compound **2f** was also found to be cytotoxic against the breast cancer MCF-7 cell line.

## 1. Introduction

The World Health Organization (WHO) has predicted that neurodegenerative diseases (NDs) that mainly affect the motor functions will overtake cancer to become the second-most prevalent cause of death after cardiovascular diseases [1,2]. Alzheimer‘s disease (AD) is one of the most severe neurodegenerative conditions that affect elderly people throughout the world and its symptoms are characterized by a progressive loss of memory, impaired decision-making, decline in behavior and function [3]. Several mechanisms are involved in AD including the cholinesterase hypothesis, β-amyloid cascade, oxidative stress and metal imbalance [4] as well as inflammation [5] and immune suppression [6]. Chronic inflammation is a common phenomenon present in the background of multiple neurodegenerative diseases and neuroinflammation has become to be recognized as an important pathophysiological feature of AD [7,8]. Lipoxygenases (LOXs) are a class of nonheme iron-containing enzymes that catalyze the hydroperoxidation of polyunsaturated fatty acids regio- and stereospecifically. The three main lipoxygenase enzymes found in humans are lipoxygenase-5 (LOX-5), lipoxygenase-12 (LOX-12) and lipoxygenase-15 (LOX-15) [9,10]. Lipoxygenases have been implicated in neurodegenerative diseases including AD. LOX-5 inhibitors, for example, reduce neuro-inflammation, amyloid plaques and neurofibrillary tangles, which are hallmarks of Alzheimer’s disease [8]. Oxidative stress has also been implicated in neurological disorders such as Alzheimer’s disease [11]. Treatment of AD focuses predominantly on reducing cognitive decline with acetylcholinesterase (AChE) and butyrylcholinesterase (BChE) inhibitors [12]. However, AD has many facets and contributing factors and inhibition of cholinesterase enzymes does not slow progression of the disease, but only helps to alleviate some of the symptoms [13]. In an attempt to address the complexity of AD, it has become necessary to design and develop drugs with increased potency capable of controlling multiple targets involved in this disease at the same time. This multi-target-directed ligand design strategy has been found to be more effective for the treatment of AD [14,15]. Moreover, it reduces the risk associated with poor patient compliance, drug–drug interactions and pharmacokinetic differences between the individual drugs.

**Figure 1:**
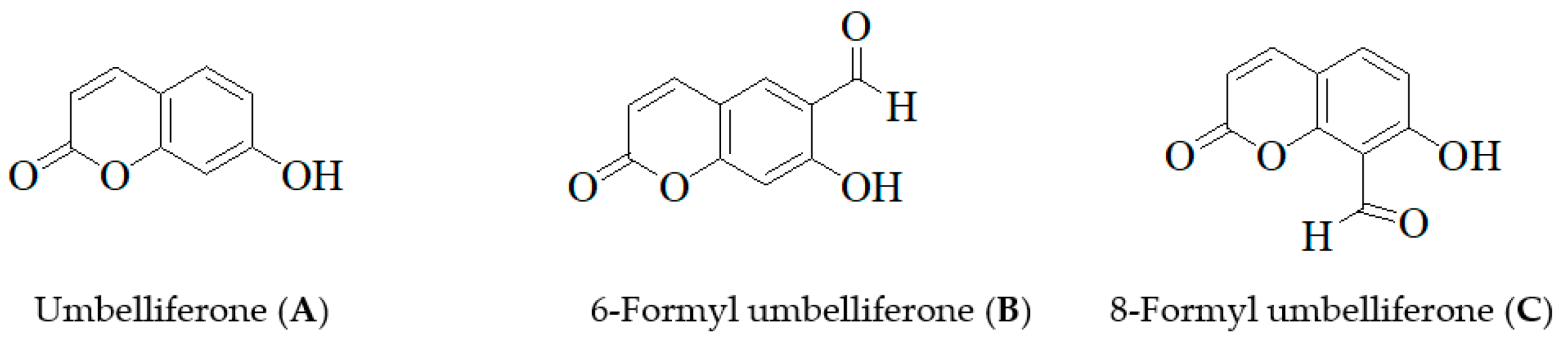
Chemical structures of unsubstituted (**A**), 6-formyl (**B**) and 8-formyl (**C**) umbelliferones.

Benzo[*b*]furan-based compounds exhibit cytotoxic, antimicrobial, anti-inflammatory, anti-asthma, anti-oxidant and anti-Alzheimer’s properties [16,17,18,19]. This moiety has become an important pharmacophore in the design of cholinesterase inhibitors [20]. Chromone moiety is also prevalent in compounds with pharmacological application in the field of neurodegenerative, inflammatory and infectious diseases as well as diabetes and cancer [21,22]. The analogous coumarins **A** and **B** (Figure 1) previously isolated from *Angelica decursiva* were evaluated along with the synthetic isomer **C** for anti-Alzheimer’s properties in vitro against AChE, BChE and β-secretase [23]. Compound **B** substituted with a carbaldehyde group at the 6-position was found to exhibit strong anti-Alzheimer’s activity than compound **A** and **C**. Based on this literature precedent and the anti-Alzheimer’s activity of benzofuran- and chromone-based compounds, we decided to integrate benzofuran, chromone and carbaldehyde moieties on the same molecular framework to afford furochromone carbaldehyde derivatives. The intention was to enrich the biological profile of the resulting furochromone carbaldehyde derivatives as potential multi-target drugs against the following targets involved in AD diseases: cholinesterases (AChE and BChE), β-secretase (BACE-1) and lipoxygenases (LOX-5 and LOX-15), as well as for anti-oxidant potential.

## 2. Results and Discussion

### 2.1. Chemistry

**Scheme 1:**
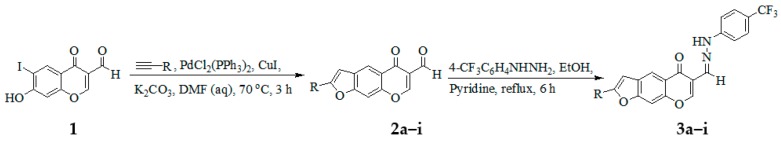
Synthesis of chromone-6-carbaldehydes **2a**–**i** and their hydrazone derivatives **3a**–**i**.

**Figure 2:**
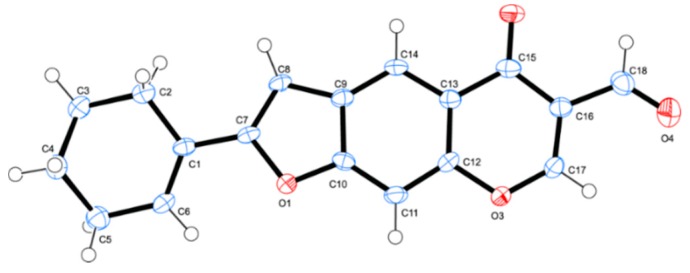
Oak ridge thermal ellipsoid plot (ORTEP) diagrams (50% probability level) of **2i**. For clarity, hydrogen atoms are not labeled.

Our strategy involved initial tandem Sonogashira cross-coupling and cycloisomerization of 7-hydroxy-6-iodo-4-oxo-4*H*-chromene-3-carbaldehyde (**1**) with terminal acetylenes followed by transformation of the intermediate furochromone-6-carbaldehydes (**2**) into hydrazono derivatives (3) as shown in Scheme 1. Compound **1** was prepared in 68% yield by treatment of 2,4-dihydroxy-5-iodoacetophenone with phosphoryl oxychloride–dimethyl formamide (POCl_3_/DMF) mixture at room temperature (RT) for 12 h. The ^1^H-NMR spectrum of compound 1 is distinguished from that of the substrate by the absence of the methyl and hydroxyl proton signals and revealed the presence of a set of singlets at δ 8.50 ppm and δ 10.27 ppm, which correspond to H-2 and CHO, respectively. Its ^13^C-NMR spectrum, on the other hand, revealed the presence of increased number of carbon signals in the aromatic region and the additional carbonyl signal at δ 189.2 ppm corresponding to the formyl group. The *o*-iodophenolic moiety has previously been found to undergo tandem palladium catalyzed Sonogashira cross-coupling and heteroannulation with terminal acetylenes to afford benzofuran derivatives [24]. Next we exploited this strategy on 1 with various terminal acetylenes in the presence of dichlorobis(triphenyl)phosphine(II) as a source of active Pd(0) species and CuI as co-catalyst using potassium carbonate as a base in aqueous dimethyl formamide (DMF) at RT for 3 h. Aqueous work-up and purification by silica gel column chromatography afforded the corresponding 2-aryl-5-oxo-5*H*-furo[3,2-*g*]chromene-6-carbaldehydes **2a**–**i** (see Table 1 for the designation of substituent R). The ^1^H- and ^13^C-NMR spectra of compounds **2a**–**h** revealed the presence of increased number of proton and carbon signals in the aromatic region. The aliphatic protons of **2i** resonated as set of multiplets in the region δ 1.63–2.39 ppm with the signal for the vinylic proton at δ 6.64 ppm. The accurate calculated *m*/*z* values for compounds 2a–i are consistent with the assigned molecular structures. Single crystal X-ray diffraction (XRD) analysis of the 2-(cyclohex-1-en-1-yl)-5-oxo-5*H*-furo[3,2-*g*]chromene-6-carbaldehyde (**2i**) distinctly confirmed the tricyclic structure of these compounds (Figure 2) [25]. The analogous 2-unsubstituted 5-oxo-5*H*-furo[3,2-*g*]chromene-6-carbaldehyde derivative was prepared before via the Vilsmeier–Haack reaction of 1-(6-hydroxybenzofuran-5-yl)ethanone in the presence of DMF-oxalyl chloride mixture [26]. 3-Hydrazones possessing an azomethine proton (–C**H**=N–NH) constitute an important class of compounds for new drug development [27]. Since drugs targeting the CNS need to be able to pass through the blood–brain barrier [28], we opted for the use of 4-trifluoromethylphenylhydrazine as the nucleophile to transform the strongly electrophilic 3-carbaldehyde group of compounds **2a**–**i** into hydrazono functionality. A trifluoromethyl (–CF_3_) group has previously been found to increase biological activity of the molecule due to its enhanced lipid solubility and metabolic stability often coupled with diminished side effects [29]. Compounds **2a**–**i** were treated with 4-(trifluoromethylphenyl)hydrazine in the presence of pyridine as a base in ethanol under reflux followed by aqueous work-up and purification by column chromatography on silica gel to afford the corresponding hydrazono derivatives **3a**–**i** in high yield and purity. The structures of the compounds were characterized using a combination of NMR (^1^H-, ^13^C- and ^19^F-), IR and mass spectrometric techniques.

About 47% of all current drugs in the market have been found to inhibit enzyme targets [30]. The potential for a compound to become a drug can be revealed through mechanism of action of the target enzyme. Consequently, compounds **2a**–**i** and their hydrazono derivatives **3a**–**i** were evaluated for inhibitory properties against acetylcholinesterase and butyrylcholinesterase activities. The most active compounds from each series were, in turn, evaluated against the following enzyme targets, β-secretase (BACE-1) and lipoxygenases (LOX-5 and LOX-15), which are also involved in Alzheimer’s disease. The structure activity relationship (SAR) of these compounds was studied with respect to the substitution pattern of the phenyl substituent on the benzofuran ring and the effect of transforming the hydrogen bond accepting carbaldehyde into hydrogen bond donor hydrozone moiety.

### 2.2. Biology

#### 2.2.1. Evaluation for Anti-Cholinesterase Activities

The in vitro AChE and BChE inhibitory activities of compounds **2a**–**i** and **3a**–**i** together with the standard drugs, donepezil and galantamine, were evaluated by Ellman’s method. Donepezil is a potent, selective, uncompetitive and reversible acetylcholinesterase (AChE) inhibitor that has been found to enhance cholinergic function by increasing ACh levels in the central nervous system [31]. Galantamine, on the other hand, is a centrally acting, selective, reversible and competitive acetylcholinesterase (AChE) inhibitor, as well as an allosteric modulator of the neuronal nicotinic receptor for acetylcholine [32]. The corresponding results against AChE and BChE are expressed as means of IC_50_ values (the concentration that inhibits enzyme activity by 50%) in µM and the data were obtained from triplicate runs (Table 2). The 5-oxo-chromene-6-carbaldehyde derivatives within the series **2a**–**i** were found to exhibit variable inhibiting effect against AChE and BChE relatively less than the activity of the reference standards donepezil and galantamine. A phenyl group at the 2-position of furan ring resulted in reduced activity of compound **2a** against AChE and moderate inhibitory effect against BChE activity with IC_50_ values of 28.6 µM and 12.3 µM, respectively. Compound **2b** substituted with a 3-fluorophenyl group at the 2-position of the furan ring exhibited significant inhibitory effect against AChE and BChE activities with IC_50_ values of 5.4 µM and 6.4, µM, respectively. The presence of fluorine at the para position of the phenyl substituent in isomer **2c**, on the other hand, resulted in reduced inhibitory effect against both enzymes. Likewise, the presence of chlorine at meta or para position of the phenyl ring of **2d** and **2e** also resulted in reduced inhibitory effect against both enzymes. Compound **2f** substituted with a 4-methoxyphenyl group on the furan scaffold, on the other hand, exhibited significant inhibitory effect against AChE (IC_50_ = 9.4 µM) and comparable activity to donepezil against BChE with IC_50_ value of 5.1 µM. The presence of a bulky 3,5-dimethoxyphenyl group in **2g** resulted in significantly reduced activity against both enzymes when compared to the 4-methoxyphenyl substituted derivative **2f** with IC_50_ values of 10.8 µM (AChE) and 41.2 µM (BChE). A non-lipophilic 4-methyl group on the 2-phenyl ring of **2h**, on the other hand, imparted moderate inhibitory effect against AChE (IC_50_ = 12.9 µM), but significantly reduced inhibitory effect against BChE (IC_50_ = 28.1 µM). Compound **2i** substituted with a cyclohexenyl moiety instead of a phenyl substituent was found to be less active against AChE and even less so against BChE.

Replacement of the hydrogen bond accepting 6-carbaldehyde moiety of **2a**–**i** with a hydrogen bond donor hydrazono functionality in compounds **3a**–**i** resulted in improved inhibitory effect only for compounds **3b**, **3d** and **3f** substituted at the C-2 position of the furan moiety with a 3-fluorophenyl-, 3-chlorophenyl- or 4-methoxyphenyl- group, respectively. (Table 3) The 3-fluorophenyl-6-hydrazono furochromen-5-one derivative **3b** exhibited significant inhibitory effect against AChE activity (IC_50_ = 7.6 µM), but relatively reduced activity against BChE (IC_50_ = 11.2 µM) when compared to the activity of the corresponding substrate **2b** (IC_50_ = 5.4 and 6.4 µM, respectively). The 2-(4-fluorophenyl)–substituted hydrazono derivative **3c** was found to be slightly more active against BChE (IC_50_ = 14.1 µM) than the corresponding substrate **2c** (IC_50_ = 15.7 µM). The observed results for the 3-fluorophenyl- **2b** and **3b** versus 4-fluorophenyl substituted derivatives **2c** and **3c** seem to suggest that a fluorine atom is more favorable for inhibitory activity against AChE activity when it is at the meta position of the 2-phenyl ring. A combination of 2-(3-chlorophenyl) group and 6-hydrazono moiety imparted significant inhibitory properties on compound **3d** against BChE (IC_50_ = 7.2 µM) than AChE (IC_50_ = 18.3 µM). The isomeric 2-(4-chlorophenyl)hydrazono derivative **3e** was found to be moderately active against AChE (IC_50_ = 12.9 µM) and less active against BChE (IC_50_ = 24.6 µM). AChE has been found to predominate over BChE in the healthy brains, however, its activity is known to decline during the development of AD while BChE activity increases. Although selective inhibition of BChE can lead to adverse peripheral side effects [33], compound **3d**, which is selective against BChE activity, may be more beneficial in alleviating the cognitive symptoms on moderate forms of AD. The presence of the hydrazono moiety in the case of the 4-methoxyphenyl substituted derivative **3f** (IC_50_ values of 5.4 µM and 9.6 µM, respectively) led to a reverse trend in inhibitory effect to that of **2f** (IC_50_ = 9.4 and 5.1 μM, respectively) against AChE and BChE activities. No significant differences in inhibitory effect against both enzymes were observed between **2g** and its hydrazono derivative **3g** substituted with a bulky 3,5-dimethoxyphenyl group at the C-2 position of the furan framework. Significantly reduced activities against both enzymes were observed for the non-lipophilic 4-methylphenyl- **3h** and the cyclohexenyl substituted hydrazono **3i** derivatives.

AChE is known to promote amyloid-β (Aβ) fibril formation and enhancing its neurotoxicity through its peripheral anionic site (PAS) [34]. The design of ChE inhibitors that simultaneously block both the catalytic and peripheral anionic sites of AChE and the catalytic activity of BChE may lead to a dual benefit, namely increasing cholinergic transmission and potentially slowing down the formation of the neurotoxic extracellular plaques [35]. With the aim to discover compounds that could target different pathological features of Alzheimer’s disease, we decided to evaluate the most active compounds from each series, namely, **2b** and **2f**, and the hydrazono derivatives **3b**, **3d** and **3f** with a potential dual inhibitory effect against AChE and BChE activities for a inhibitory effect against β-secretase activity.

#### 2.2.2. Evaluation of Compounds **2b**, **2f**, **3b**, **3d** and **3f** Against β-Secretase (BACE-1) Activity

β-Site amyloid precursor protein cleaving enzyme 1 (β-secretase) plays a significant role in the cleavage of amyloid precursor protein, which leads to the production of α,β-peptide. Overproduction of amyloid beta (Aβ) by β-secretase has been found to result in toxic fibrils, which cause a neurodegeneration characteristic of Alzheimer’s disease [36]. β-Secretase is considered the initial and rate-limiting step in Aβ production, which makes it another target for the treatment and prevention of AD. The most active compounds **2b**, **2f**, **3b**, **3d** and **3f** against AChE and/or BChE were evaluated for potential to inhibit β-secretase activity (Table 4). Compound **2b** with dual inhibitory potential against AChE (5.4 µM) and BChE (6.4 µM) was found to exhibit moderate activity against β-secretase (IC_50_ = 21.6 µM) when compared to the activity of the reference standard quercetin (IC_50_ = 12.5 µM). The presence of a strongly electron-delocalizing 4-methoxyphenyl group on the furochromone-6-carbaldehyde framework resulted in significant activity against β-secretase for **2f** (IC_50_ = 15.4 µM) with dual cholinesterase inhibitory potential. The 3-chlorophenyl–substituted hydrazono derivative **3d**, which showed selectivity against BChE activity (IC_50_ = 7.2 µM) was found to be less active against β-secretase (IC_50_ = 32.8 µM). Replacement of the carbaldehyde moiety of **2f** with a hydrazono group resulted in significantly reduced inhibitory effect for derivative **3f** (IC_50_ = 25.3 µM) against β-secretase.

Studies on animal models previously showed that either 5- or 15-LOX inhibitors have the capability of reducing amyloid and tau pathology as well as improving cognitive impairment [37]. Experimental evidence also highlighted a correlation between these lipoxygenases with oxidative stress in AD patients [38].

#### 2.2.3. Inhibition of Lipoxygenase-15 by **2b**, **2f**, **3b**, **3d** and **3f**, and Lipoxygenase-5 by **2f**

Compounds **2b**, **2f**, **3b**, **3d** and **3f** were also evaluated for potential to inhibit lipoxygenase-15 (Soybean LOX-15) and the corresponding results of this assay are represented in Table 5. These compounds exhibited variable inhibitory activity against LOX-15 with IC_50_ values ranging from 9.6 μM to 26.5 μM against quercetin as a reference standard (IC_50_ = 3.4 μM). Quercetin has been used as a reference standard because it has previously been found to inhibit production of inflammation-producing cyclooxygenases (COX) and lipoxygenases (LOX) [39]. Compound **2b**, which exhibits dual cholinesterase activity and moderate inhibitory effect against β-secretase, was found to be moderately inhibiting against LOX-15 with IC_50_ of 16.3 μM. A significant inhibitory effect against LOX-15 was observed for **2f (**IC_50_ = 9.6 μM), which has been found to exhibit dual anti-cholinesterase and anti–β-secretase properties (see Table 2 and Table 4 above). Compound **2f** was, in turn, evaluated for potential to inhibit human lipoxygenase-5 activity against quercetin and zileuton as the reference standards. Although not better than both reference standards, compound **2f** also displayed significant inhibitory effect against LOX-5 (Table 5).

Numerous LOX inhibitors are known to exhibit inhibitory effects via antioxidant mechanisms by either scavenging the radicals or preventing oxidation of iron at the active site thereby inhibiting enzyme activity [40]. The efficiency of the brain’s anti-oxidant system, on the other hand, has been found to decrease gradually with age, and this effect becomes more pronounced in patients with Alzheimer’s disease [41]. Evaluation of the antioxidant potential becomes an essential part of the LOX inhibition study and drugs that could prevent the formation of free radicals in the brain would be beneficial against AD [42]. The free radical-scavenging activities of compounds **2b**, **2f**, **3b**, **3d** and **3f** were evaluated against the natural antioxidant ascorbic acid as a reference standard. The results are expressed as IC_50_ values, i.e., the concentration of each sample required or able to scavenge 50% of the 2,2-diphenyl-1-picrylhydrazyl (DPPH). Preliminary DPPH radical scavenging results (Table 6) revealed that the most active furochromone-3-carbaldehydes and hydrazono derivatives against AChE and/or BChE activity exhibit moderate anti-oxidant activity (IC_50_ 7.4–23.9 µM) when compared to the reference standard, ascorbic acid (IC_50_ = 4.8 µM). Though less active when compared to the activity of the reference standard, compound **2b** is capable of scavenging free radicals. Compound **2f**, which exhibits significant inhibitory properties against cholinesterases and β-secretase as well as lipoxygenases, was also found to exhibit moderate anti-oxidant activity with IC_50_ value of 18.1 µM.

Understanding the mechanism of action of the target enzyme is critical in early discovery and development of drug candidates. Consequently, kinetic studies were undertaken in order to elucidate the plausible mechanisms of inhibition of AChE, BChE and BACE-1 by **2b**, **2f**, **3b**, **3d** and **3f**.

#### 2.2.4. Kinetic Studies of **2b**, **2f**, **3b**, **3d** and **3f** against AChE, BChE and BACE-1

The mechanisms of action of these compounds against cholinesterases or β-secretase were evaluated by building the Lineweaver–Burk double reciprocal plots and the Dixon plots at increasing inhibitor and substrate concentrations (0.1, 0.5, 2.5 and 5 µM). The corresponding graphs against AChE (Figure S2.1), BChE (Figure S2.2) and BACE-1 (Figure S2.3) form part of the Supplementary Information. The Lineweaver–Burk plots of compounds **2b**, **3b**, **3d** and **3f** against AChE revealed a decrease in the velocity of the reaction when the enzyme is fully saturated by substrate (V_max_ values 0.066−0.018, 0.055−0.018, 0.037−0.018 and 0.08−0.017 respectively) and relatively unchanged Michaelis constant (K_m_) values of 0.19 ± 0.02, 0.22 ± 0.03, 0.18 ± 0.005 and 0.20 ± 0.01, respectively. The Dixon plots of inverse of the velocity of the reaction versus inhibitor concentrations for compounds **2b**, **3b**, **3d** and **3f** produced intersecting lines on the x-axis and K*_i_* values of 1.1 ± 0.02, 0.58 ± 0.04, 2.5 ± 0.01 and 8.16 ± 0.02, respectively. The observed kinetic results suggest that these compounds exhibit non-competitive modes of inhibition against AChE activity. The Lineweaver–Burk plots for the most active compound **2f** against this enzyme target, on the other hand, displayed an increase in the Michaelis constant values (K_m_ = 0.20–0.24) with relatively unchanged V_max_ (0.03 ± 0.018) value. This behavior suggests a competitive mode of enzyme inhibition against AChE activity by this compound. Dixon plot for this compound produces intersecting lines above the x-axis, which also confirms the competitive mode of inhibition and provides a K_i_ value of 1.34 ± 0.02. The Lineweaver–Burk plots of compounds **2b**, **2f**, **3b** and **3d** against BChE activity at substrate concentrations of 0–5 µM also displayed decreases in the V_max_ values (0.018–0.009, 0.025–0.01, 0.0035–0.016 and 0.026–0.011, respectively) with relatively unchanged K_m_ values (0.1 ± 0.008, 0.21 ± 0.006, 0.185 ± 0.02 and 0.23 ± 0.02, respectively). This observation suggests a non-competitive mode of inhibition against BChE for these compounds, which was confirmed by the Dixon plot analysis. The calculated K_i_ values for **2b**, **2f**, **3b** and **3d** against BChE are 1.7 ± 0.01, 3.9 ± 0.01, 6.1 ± 0.02 and 0.85 ± 0.01, respectively. The K_m_ values for **3f** (0.21–0.31) against BChE activity increase with less or no changes of V_max_ value (0.013 ± 0.02), and this observation suggests a competitive mode of enzyme inhibition for this compound. The Dixon plot for compound **3f** was used to determine a K*_i_* value of 5.4 ± 0.04 and confirm the mode of inhibition. Compound **2f** with dual inhibitory effect against cholinesterases was selected for evaluation of mode of action against β-secretase. The K_m_ value at inhibitor concentrations, 0, 4, 8 and 16 µM, remained constant (0.002) with decreasing V_max_ (0.02–0.005) indicating a non-competitive mode of inhibition. The Dixon plot was used to determine the K*_i_* value of 1.49 ± 0.01 and displayed x-intercept above the x-axis indicative of a competitive mode of inhibition. These observations support a mixed mode of inhibition of this enzyme by **2f**, displaying a mixture of competitive and non-competitive inhibition.

In order to figure out the plausible protein-ligand interactions at molecular level and to rationalize the structure activity relationship, we performed molecular dockings of the most active compounds into the active pockets of AChE (PDB: 1GQR) and BChE (PDB: 1P0I). Compound **2f** was also docked into the active sites of β-secretase (PDB: 3IXJ) and LOX-5 (PDB: 3O8Y).

### 2.3. Molecular Docking Studies

#### 2.3.1. Molecular Docking Studies of **2b**, **2f**, **3b**, **3d** and **3f** into AChE (PDB: 1GQR) Active Sites

**Figure 3:**
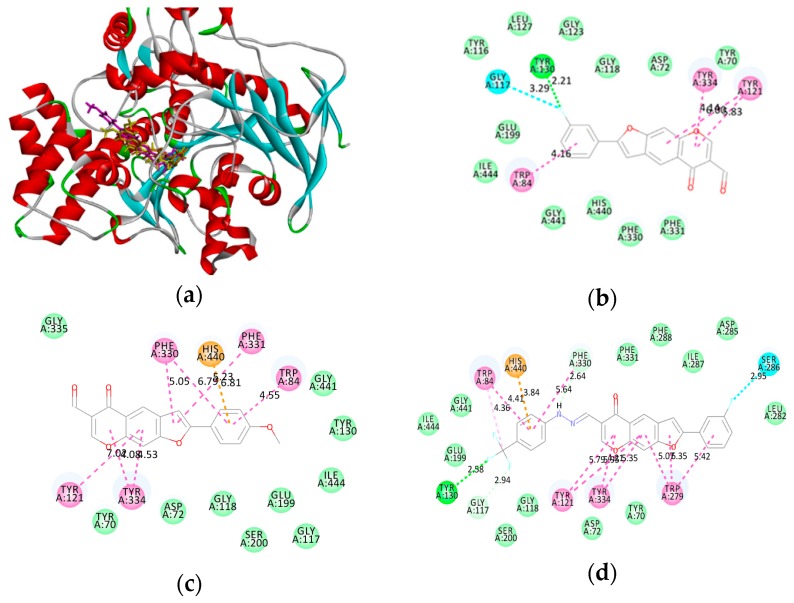

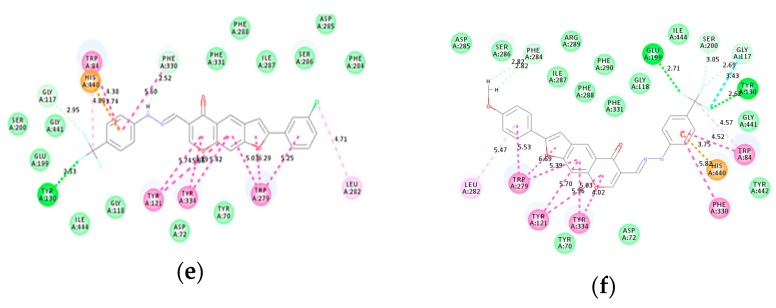
Docking poses of donepezil (yellow), **2b** (orange) and **3f** (purple) represented with AChE (1GQR) in ribbon format (**a**). The 2-dimensional (2D) plots of docking results for **2b** (**b**), **2f** (**c**), **3b** (**d**) **3d** (**e**) and **3f** (**f**) into AChE including bonding interaction distances. Bright green represents conventional hydrogen bonds, light green—van der Waals interactions, very light green carbon hydrogen bonds, dark pink—pi–pi interactions, light pink—pi–alkyl interactions, blue—halogen (florine) interactions and orange—pi–cation interactions.

The crystal structure of AChE with rivastigmine co-crystallized was downloaded from the Protein Data Bank (PDB code: 1GQR) and used in this investigation. Donepezil was docked into the active site of this crystal and the top scoring docked pose with the calculated binding free energy (BE) of –73.50 kcal/mol was applied as starting point for molecular dockings (see Figure S3 in the Supplementary Information for its interactions with the AChE residues). Donepezil has been found to interact with both the catalytic active site (CAS) and the peripheral anionic site (PAS) tryptophans via ring-stacking interactions [35]. The compounds were docked individually into the active site of AChE using the same parameters and site as for the docking of donepezil. Figure 3a shows the orientation of the most active furochromone carbaldehyde **2b** (orange) and the hydrazono derivative **3f** (purple) docked into the same site of AChE as donepezil (yellow). Comparison of the calculated binding free energies for the top scoring docked poses of these compounds into the active site of AChE revealed that **2f** is more favorable for binding to AChE than the other derivatives (Table 7). The binding conformations of the furochromone carbaldehydes **2b** and **2f** as well as those of the hydrazono derivatives **3b**, **3d** and **3f** were similar and their interactions with protein residues of AChE are represented in Figure 3. Molecular docking of **2b** (Figure 3b) or **2f** (Figure 3c) into the active site of AChE predicts aromatic–aromatic (pi–pi stacked and pi–pi T-shaped) interactions between chromen-4-one framework and the protein residue Tyr121 and Tyr334 (PAS residue), as well as between the aryl substituent and Trp84. The 2-(3-fluorophenyl)- moiety of **2b** is involved in hydrogen and halogen bonding interactions with the protein residues, Tyr130 and Gly117 in the active site, respectively. These interactions could explain the enhanced non-competitive mode of inhibition observed for **2b**. The methoxyphenyl ring of **2f**, on the other hand, is involved in pi-cation interaction with His440 of the catalytic triad, which might explain its competitive mode of inhibition as demonstrated by the kinetics studies against this enzyme. Although **2b** is more inhibiting against AChE than **2f** molecular docking predicts more interactions of the latter with several protein residues including His440 of the catalytic triad consistent with the calculated binding energies of these compounds −56.7 and −66.8 kcal/mol, respectively. The furochromenone scaffold and the phenyl ring of **3b** (Figure 3d) and **3d** (Figure 3e) are involved in pi–pi stacking and pi–pi T-shaped interactions with the protein residues Tyr121, Tyr279 and Tyr334 residues. The 4-trifluorophenyl wing in both cases is involved in aromatic–aromatic stacking interaction with Trp84 and phenylalanine (Phe330) in the acyl binding pocket as well as the pi–cation interaction with His440 in the catalytic triad. The hydrogen bonding interaction is predicted between the trifluoromethyl group and Tyr130 for both compounds. The halogen bonding interaction is also predicted between the 3-(fluorophenyl) group of **3b** and Ser286 whereas chlorine atom of **3d** is involved in alkyl and pi-alkyl interaction with Leu282. The active site of this enzyme is a large cleft, which may also allow binding of the substrate and corroborate the non-competitive behavior of **3b** and **3d** observed through kinetic studies. The furochromenone scaffold of compound **3f** (Figure 3f) substituted with a lipophilic 4-methoxyphenyl group on the furan ring is involved in several aromatic-aromatic (pi–pi and pi-T-shaped) stacking interactions. The 4-trifluorophenyl ring of this compound is involved in pi–pi stacking interaction with phenylalanine (Phe330) in the acyl binding pocket and in the pi–cation interaction with His440 of the catalytic triad. Hydrogen and halogen bonding interactions are predicted between the trifluoromethyl group and the protein residues Tyr130 and Gly117, respectively. An additional hydrogen bonding interaction is also predicted between the trifluoromethyl group of **3f** and Glu199. The hydrazone derivatives seem to penetrate the aromatic cleft of the AChE. The 5-oxo-5*H*-furo[3,2-*g*]chromene-6-carbaldehydes and their 6-(4-trifluoromethyl)phenylhydrazone derivatives interact with protein residues in both the peripheral anionic site (PAS: Trp279 and/or Tyr334) and catalytic anionic site (CAS: His440) site of AChE. The trend in the calculated binding energies for the hydrazono derivatives **3b** (−75.0 kcal/mol), **3d** (−67.5 kcal/mol) and **3f** (−85.0 kcal/mol) against AChE is consistent with their IC_50_ values, which showed the following trend 5.4 (**3f**) > 7.6 (**3b**) > 18.3 μM (**3d**).

#### 2.3.2. Molecular Docking Studies of **2b**, **2f**, **3b**, **3d** and **3f** into BChE (PDB: 1P0I) Active Sites

**Figure 4:**
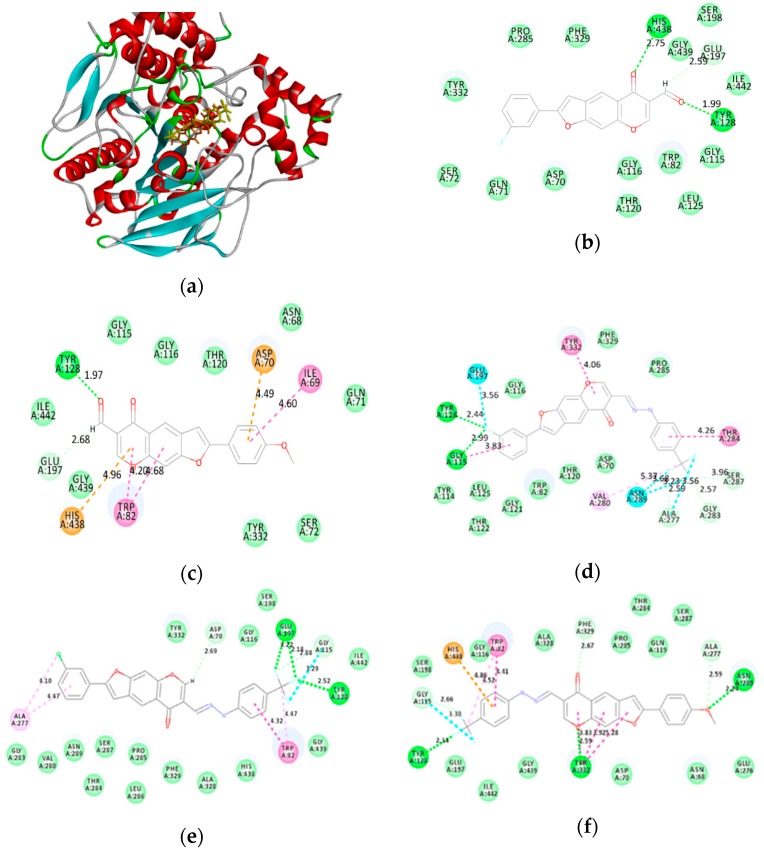
Docking poses of donepezil (yellow) and **2f** (orange) represented with BChE (1P0I) in ribbon format (**a**). 2D plots of docking results for **2b** (**b**), **2f** (**c**), **3b** (**d**), **3d** (**e**) and **3f** (**f**) into BChE including the bonding interaction. Bright green represents conventional hydrogen bonds, light green—van der Waals interactions, very light green carbon hydrogen bonds, dark pink—pi–pi interactions, light pink—pi–alkyl interactions, orange—pi–anion or pi–cation interactions and blue—halogen (fluorine) interactions.

Figure 4a shows the overlaid docking poses of donepezil (yellow) and the most active compound **2f** (orange) in the active site of BChE. The binding conformations and interactions of **3b**, **3d** and **3f** with protein residues in the active site of this enzyme are represented as Figure 4a–f (see Figure S4 in the Supplementary Information (SI) for the interaction of donepezil with the residues of BChE). The carbaldehyde hydrogen atom of **2b** (Figure 4b) is involved in weak van der Waals and carbon hydrogen bonding interactions with Gly439 and Glu197, respectively. Two hydrogen bonding interactions are predicted between the ketone and carbaldehyde moieties with the protein residues histidine-438 of the catalytic triad and Tyr128, respectively. This compound might also bind additional alternative sites on the enzyme consistent with its non-competitive inhibition mode. The furochromenone scaffold of **2f** (Figure 4c) is involved in several weak aromatic–aromatic (pi–pi and amide–pi) stacking interactions with Ile69 and Trp82 as well as the pi–cation interaction with imidazole ring of His438 and pi–anion interaction with Asp70. Hydrogen and oxygen atoms of the carbaldehyde functionality are involved in weak van der Walls interactions and hydrogen bonding interactions with Glu197 and Tyr128, respectively. These interactions corroborate the non-competitive behavior observed through kinetic studies that confirmed that this compound binds to the active site (while not excluding substrate binding) and elsewhere on the protein affecting enzyme activity. The calculated binding energies of **2b** (−60.48 kcal/mol) and **2f** (−62.80 kcal/mol) shown in Table 8 were consistent with their IC_50_ values of 6.4 and 5.1 μM against this enzyme. The protein residues Gly115, Tyr332 and Thr284 were involved in aromatic–aromatic stacking interactions with the 3-fluorophenyl, pyran-4-one and 4-(trifluoromethyl)phenyl rings of **3b** (Figure 4d), respectively. Halogen bonding interaction is observed between the 3-fluorophenyl moiety and Glu197 and also between the trifluoromethyl group and the protein residues Asn289 and Ala277. The fluorine atom of the 3-fluorophenyl ring was involved in hydrogen bonding interactions with Tyr128 and Gly115. Alkyl and pi–alkyl interactions were predicted between the 3-chlorophenyl ring and trifluoromethyl group of **3d** with Ala227 (Figure 4e). There was also a pi–pi stacking interaction predicted between the phenyl ring of 4-(trifluoromethyl)phenyl group of this compound with Try82. The protein residues Glu197 and Tyr128 were predicted to be involved in hydrogen bonding interaction with the trifluoromethyl group, which was also involved in halogen bonding interaction with the protein residue Gly115. Molecular docking predicts a non-competitive behavior for this compound in agreement with results from the kinetic study. The planar furochromenone scaffold and the arylhydrazone moiety of **3f** (Figure 4f) were involved in aromatic–aromatic (pi–pi) stacking interactions with Tyr332 and Trp82, respectively. The latter was also involved in weak pi–alkyl interaction with the trifluoromethyl group. The phenyl ring of the hydrazone wing was also involved in a pi–cation interaction with His438 of the catalytic triad with its trifluoromethyl group involved in halogen bonding interaction with Gly115. Hydrogen bonding interactions existed between the trifluoromethyl group with Tyr128 and between pyran-4-one endocyclic oxygen and Tyr332, as well as between the methoxy oxygen and Asn289. This compound interacts strongly via hydrogen bonding interactions with the active site, which probably account for the competitive behavior and relatively increased inhibitory effect against BChE activity. The larger size of the BChE binding pocket has probably enabled a bent conformation of the 4-(trifluoromethyl)phenylhydrazono moiety of **3b**. The relatively distinct orientation of **3b** (BE = −37.07 kcal/mol; IC_50_ = 11.2 μM) compared to **3d** (BE = −49.14 kcal/mol; IC_50_ = 7.2 μM) may be one of the possible reasons behind its lower binding strength and reduced inhibitory effect. Although **3f** (IC_50_ = 9.6 μM) is relatively less active than **3d** against BChE, molecular docking predicts the former to bind strongly to the active site with the calculated binding energy of −62.59 kcal/mol. This is presumably because the hydrazono arm of **3f** is deeply anchored within the catalytic site to interact with His438 of the catalytic triad and this interaction may account for the envisaged binding strength of this compound.

#### 2.3.3. Molecular Docking Studies of **2f** into β-Secretase Active Site

**Figure 5:**
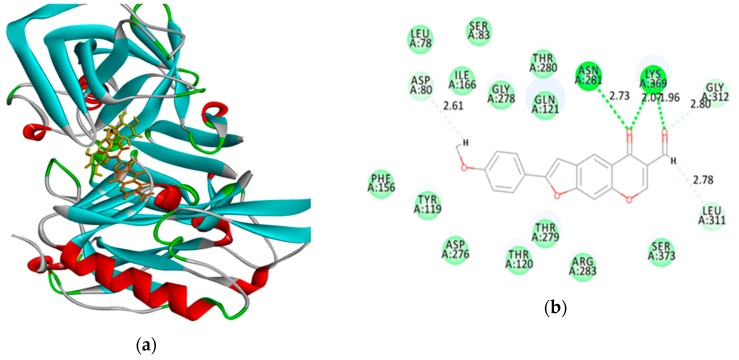
Quercetin (yellow) and **2f** (orange) represented with β-secretase (3IXJ) in ribbon format (**a**), and 2D docking plot for **2f** including bonding interaction distances (**b**). Bright green represents conventional hydrogen bonds, light green—van der Waals interactions and very light green carbon hydrogen bonds.

Quercetin was docked into the active site of β-secretase (PDB code: 3IXJ) and the top scoring docked pose with the calculated binding free energy (BE) of −32.74 kcal/mol was applied as starting point for molecular docking (Figure 5). Molecular docking predicts several hydrogen bonding interactions between quercetin and β-secretase residues (refer to Figure S5 in the SI). The top scoring docked pose of **2f** (orange) was shown to be deeply embedded into the active pocket of β-secretase than quercetin (yellow) did (Figure 3a). The methoxy hydrogen and hydrogen atom of the carbaldehyde moiety of **2f** were involved in the carbon hydrogen bonding interaction with Asp80 and Leu311, respectively. Weak van der Waals interaction was also predicted between the carbaldehyde oxygen and Gly312 (Figure 3b). Strong hydrogen bonding interactions were predicted between the protein residue Lys369 and both the carbonyl oxygen atom of the ketone (Hb = 2.07 Å) and carbaldehyde (Hb = 1.96 Å) functionalities. The ketone oxygen was also involved in an additional hydrogen bonding interaction with Asp281 (Hb = 2.73 Å). The binding orientation or alignment of the furochromone carbaldehyde framework of **2f** along the substrate binding cleft enabled formation of a six-membered ring involving strong hydrogen bonding interaction between the carbonyl oxygen atoms of the pyrone and carbaldehyde moieties with Lys369. This thermodynamically favorable hydrogen bonding interaction presumably increased the compound’s binding strength (BE = −92.28 kcal/mol) in the β-secretase binding pocket and therefore its inhibitory effect against this enzyme.

#### 2.3.4. Molecular Docking of **2f** into Human LOX-5 (PDB: 3O8Y) Active Site

**Figure 6:**
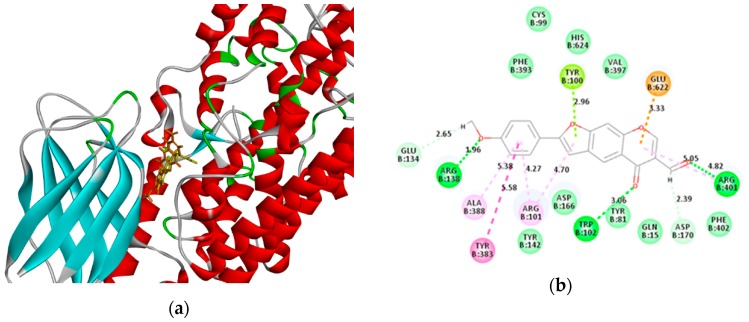
Docking poses of zileuton (yellow) and **2f** (orange) represented with LOX-5 (3O8Y) in ribbon format (**a**) and 2D docking plot for **2f** including bonding interaction distances (**b**). Bright green represents conventional hydrogen bonds, light green—van der Waals interactions, very light green carbon hydrogen bonds, dark pink—pi–pi interactions, light pink—pi–alkyl interactions, orange—pi–anion or pi–cation interactions, orange—pi–anion interactions and green yellow—pi–lone pair interactions.

The overlaid docking poses of zileuton (yellow) and **2f** (orange) into LOX-5 are represented in ribbon (Figure 6a) and the interactions of **2f** with protein residues of LOX-5 in Figure 6b. The corresponding 2D docking plot figure for zileuton is included as Figure S6 in the SI. The calculated binding energy of the top scoring pose of zileuton and of **2f** into LOX-5 were −40.22 kcal/mol and −55.62 kcal/mol, respectively. The 6- and 5-membered heterocyclic rings of **2f** were predicted to be involved in pi–anion and pi–lone pair interactions with Glu622 and Tyr100, respectively. There was a pi–alkyl interaction between the pyran-4-one ring with Arg401 and also between the furan ring and the protein residue Arg101. A similar interaction was also predicted between the phenyl group and Ala388 as well as between the furan ring and Arg101. Weak carbon hydrogen bond interactions exist between the aldehyde hydrogen and Asp170 and also between the hydrogen atom of the methoxy group and Glu134. Hydrogen bonding interactions were predicted between the carbaldehyde oxygen with Arg401, its ketone oxygen and Trp101, and also between the oxygen atom of the methoxy group and Arg138 (Hb = 1.96 Å). Hydrophobic (pi–anion, pi–lone pair and pi–alkyl) and hydrogen bonding interactions stabilized the conformation of 2f and thereby increased its binding strength to the receptor binding pocket.

Compound **2f** was also evaluated for potential anticancer properties in vitro against the breast cancer cell line MCF-7 using the anticancer drug, doxorubicin hydrochloride, as a reference standard (see Figure S7 in the SI). The preliminary 3-(4,5-dimethylthiazol-2-yl)-2,5-diphenyltetrazolium bromide (MTT) assay results indicated that 2f was moderately cytotoxic (IC_50_ = 8.65 ± 0.03 μM) against MCF-7 cancer cell line when compared to doxorubicin (IC_50_ = 1.59 ± 0.01 μM). This compound did not show any major toxicity in the Hek23-T cells till 50 μM (see Figure S7 in SI).

## 3. Experimental

### 3.1. General

The melting point (mp.) values of the prepared compounds were recorded on a thermocouple digital melting point apparatus (Mettler Toledo LLC, Columbus, OH, USA) and their IR spectra were recorded as powders using a Bruker VERTEX 70 FT-IR Spectrometer (Bruker Optics, Billerica, MA, USA) with a diamond ATR (attenuated total reflectance) accessory by using the thin-film method. For column chromatography, we employed Merck kieselgel 60 (0.063–0.200 mm; Merck KGaA, Frankfurt, Germany) as a stationary phase. The nuclear magnetic resonance (NMR) spectra were recorded as DMSO-*d*_6_ solutions on a Varian Mercury 300 MHz NMR spectrometer (Varian Inc., Palo Alto, CA, USA) operating at 300 MHz (^1^H-NMR), 75 MHz (^13^C-NMR) or 282 MHz (^19^F{^1^H}-NMR). The low- and high-resolution mass spectra were recorded at an ionization potential of 70 eV using Micromass Autospec-Time-of-Flight (TOF) Double Focusing High Resolution Instrument (Waters Corp., Milford, MA, USA). The synthesis and analytical data of 5-iodo-2,4-dihydroxyacetophenone used as precursor in this investigation have been reported before [24].

### 3.2. Vilsmeier Reaction to Afford 7-hydroxy-6-iodo-4-oxo-4H-chromen-3-carbaldehyde (***1***)

Phosphoryl chloride (12.13 g, 79.1 mmol) was added slowly to a stirred mixture of 5-iodo-2,4-dihydroxyacetophenone (5.00 g, 15.8 mmol) and DMF (5.78 g 79.1 mmol) at 0 °C. The mixture was stirred at this temperature for 30 min and then for additional 12 h at room temperature (RT). The reaction mixture was poured slowly into crush ice and the product was extracted into chloroform. The combined organic phases were dried over anhydrous MgSO_4_ and the salt was filtered off. The solvent was evaporated under reduced pressure on a rotary evaporator and the residue was purified by column chromatography on silica gel to afford compound **1** as a bright yellow solid (3.40 g, 68%), mp. 195–196 °C; ν_max_ (ATR) 424, 519, 649, 768, 837, 1241, 1290, 1385, 1569, 1603, 1632, 1698, 2859, 3074, 3195 cm^-1^; δ_H_ (300 MHz, DMSO-*d*_6_) 7.15 (1H, s, H-8), 8.50 (1H, s, H-2), 8.92 (1H, s, H-5), 10.27 (1H, s, –CHO), 12.30 (1H, s, –OH); δ_C_ (75 MHz, DMSO-*d*_6_) 86.1, 103.2, 119.3, 120.5, 136.3, 157.7, 162.8, 163.6, 173.7, 189.2; HRMS (ES^+^): *m*/*z* [M + H]^+^ calculated (calc.) for C_10_H_6_O_4_I: 316.9311; found 316.9308.

### 3.3. Typical Procedure for Tandem Sonogashira cross-coupling–heteroannulation of ***1***

A mixture of **1** (0.50 g, 1.58 mmol), PdCl_2_(PPh_3_)_2_ (0.06 g, 0.08 mmol), CuI (0.03 g, 0.16 mmol) and K_2_CO_3_ (0.33 g, 2.37 mmol) in 9:1 DMF-H_2_O (v/v, 20 mL) was placed in a two necked round-bottom flask equipped with a stirrer bar, condenser and rubber septum. The mixture was purged with argon for 30 min and a solution of phenylacetylene (0.19 g, 1.90 mmol) was introduced through the rubber septum with the aid of a syringe. The mixture was stirred at 70 °C for 3 h and then poured into an ice-cold water. The product was extracted into chloroform, and the combined organic layers were washed with brine and then dried over anhydrous MgSO_4_. The salt was filtered off, and the solvent was evaporated under reduced pressure on a rotary evaporator. The residue was purified by column chromatography on silica gel using 2:1 toluene-ethyl acetate (v/v) mixture as an eluent. Compounds **2a**–**i** were prepared in this fashion.

5-Oxo-2-phenyl-5*H*-furo[3,2-*g*]chromene-6-carbaldehyde (**2a**):

Brown solid (0.28 g, 61%), R*_f_* (toluene-ethyl acetate) 0.69, mp. 145–146 °C; ν_max_ (ATR) 495, 647, 682, 773, 950, 1083, 1230, 1301, 1379, 1446, 1469, 1567, 1616, 1644, 1698, 2853, 2922, 3068, 3097 cm^-1^; δ_H_ (300 MHz, DMSO-*d*_6_) 7.74–7.82 (3H, m, Ar), 7.90 (1H, s, H-3), 8.21 (2H, d, *J* = 6.3 Hz, Ar), 8.36 (1H, s, H-9), 8.66 (1H, s, H-7), 9.20 (1H, s, H-4), 10.41 (1H, s, –CHO); δ_C_ (75 MHz, DMSO-*d*_6_) 101.8, 112.3, 115.1, 119.4, 119.9, 121.2, 121.9, 127.2, 131.2, 150.3, 152.7, 157.4, 157.63, 160.8, 175.2, 188.9; HRMS (ES^+^): *m*/*z* [M + H]^+^ calc. for C_18_H_11_O_4_: 291.0650; found 291.0657.

2-(3-Fluorophenyl)-5-oxo-5*H*-furo[3,2-*g*]chromene-6-carbaldehyde (**2b**):

Yellow solid (0.31 g, 64%), R*_f_* (toluene-ethyl acetate) 0.60, mp. 250–251 °C; ν_max_ (ATR) 463, 524, 669, 781, 827, 868, 1229, 1318, 1447, 1476, 1569, 1619, 1644, 1700, 2853, 2923, 3096 cm^−1^; δ_H_ (300 MHz, DMSO-*d*_6_) 7.20 (1H, t, *J* = 7.0 Hz, H-5′), 7.47 (1H, d, *J* = 6.0 Hz, H-4′), 7.54 (1H, s, H-3), 7.64 (1H, s, H-2′), 7.65 (1H, d, *J* = 7.0 Hz, H-6′), 7.96 (1H, s, H-9), 8.26 (1H, s, H-7), 8.82 (1H, s, H-4), 10.0 (1H, s, –CHO); δ_C_ (75 MHz, DMSO-*d*_6_) 101.7, 103.8, 112.2 (d, ^2^*J_CF_* = 23.75 Hz), 117.0 (d, ^2^*J*_CF_ = 20.8 Hz), 118.4, 119.6, 121.6, 121.8, 128.6 (d, ^4^*J*_CF_ = 7.5 Hz), 129.4, 131.4 (d, ^3^*J*_CF_ = 8.5 Hz), 131.8 (d, ^3^*J*_CF_ = 8.5 Hz), 154.2, 157.4, 163.0 (d, ^1^*J*_CF_ = 242.8 Hz), 163.8, 175.5, 188.8; HRMS (ES^+^): *m*/*z* [M + H]^+^ calc. for C_18_H_10_FO_4_: 309.0561; found 309.0563.

2-(4-Fluorophenyl)-5-oxo-5*H*-furo[3,2-*g*]chromene-6-carbaldehyde (**2c**):

Yellow solid (0.34 g, 71%), R*_f_* (toluene-ethyl acetate) 0.61, mp. 260–261 °C; ν_max_ (ATR) 511, 648, 767, 807, 1001, 1024, 1226, 1300, 1504, 1600, 1615, 1637, 1698, 3074, 3093, 3386 cm^-1^; δ_H_ (300 MHz, DMSO-*d*_6_) 7.34 (2H, t, *J* = 9.6 Hz, H-3′,5′), 7.55 (1H, s, H-3), 7.93 (2H, dd, *J*_HH_ = 5.4 Hz and *J*_HF_ = 8.1 Hz, H-2′,6′), 8.03 (1H, s, H-9), 8.31 (1H, s, H-7), 8.84 (1H, s, H-4), 10.10 (1H, s, –CHO); δ_C_ (75 MHz, DMSO-*d*_6_) 101.6, 102.3, 116.8 (d, ^2^*J*_CF_ = 22.87 Hz), 118.0, 121.7 (d, ^4^*J*_CF_ = 2.25 Hz), 125.8, 127.8, 127.9, 129.2, 129.3, 131.8, (d, ^3^*J*_CF_ = 9.15 Hz), 154.0, 157.4, 163.2 (d, ^1^*J*_CF_ = 246.2 Hz), 175.5, 188.9; HRMS (ES^+^): *m*/*z* [M + H]^+^ calc. for C_18_H_9_O_4_F: 309.0559; found 309.0552.

2-(3-Chlorophenyl)-5-oxo-5*H*-furo[3,2-*g*]chromene-6-carbaldehyde (**2d**):

Yellow solid (0.35 g, 69%), R*_f_* (toluene-ethyl acetate) 0.67, mp. 198–199 °C; ν_max_ (ATR) 436, 646, 680, 738, 778, 951, 1095, 1306, 1446, 1476, 1601, 1621, 1661, 1694, 2853, 2923, 3070, 3107 cm^−1^ δ_H_ (300 MHz, DMSO-*d*_6_) 7.45–7.60 (2H, m, H-4′ and H-5′), 7.71 (1H, s, H-3), 7.84 (1H, d, *J* = 7.0 Hz, H-6′), 7.94 (1H, s, H-2′), 8.06 (1H, s, H-9), 8.35 (1H, s, H-7), 8.89 (1H, s, H-4) 10.09 (1H, s, –CHO); δ_C_ (75 MHz, DMSO-*d*_6_) 101.0, 111.6, 119.3, 120.8, 121.0, 122.2, 124.1, 125.1, 129.8, 131.1, 131.7, 134.5, 150.3, 156.0, 158.1, 163.0, 175.0, 189.0; HRMS (ES^+^): *m*/*z* [M + H]^+^ calc. for C_18_H_10_^35^ClO_4_: 325.0268; found 325.0261.

2-(4-Chlorophenyl)-5-oxo-5*H*-furo[3,2-*g*]chromene-6-carbaldehyde (**2e**):

Yellow solid (0.34 g, 66%), R*_f_* (toluene-ethyl acetate) 0.63, mp. 231–232 °C; max (ATR) 480, 644, 791, 811, 1011, 1092, 1296, 1444, 1471, 1489, 1567, 1609, 1651, 1696, 2852, 2921, 3078, 3102 cm^−1^; δ_H_ (300 MHz, DMSO-*d*_6_) 7.58 (2H, d, *J* = 8.1 Hz, H-2′,6′), 7.67 (1H, s, H-3), 7.93 (2H, d, *J* = 8.1 Hz, H-3′,5′), 8.10 (1H, s, H-9), 8.38 (1H, s, H-7), 8.94 (1H, s, H-4), 10.13 (1H, s, –CHO); δ_C_ (75 MHz, DMSO-*d*_6_) 100.2, 103.2, 118.3, 119.6, 127.1, 127.3, 128.1, 128.8, 129.2, 132.0, 134.8, 154.1, 157.4, 163.9, 175.5, 188.9; HRMS (ES^+^): *m*/*z* [M + H]^+^ calc. for C_18_H_10_^35^ClO_4_: 325.0268; found 325.0264.

2-(4-Methoxyphenyl)-5-oxo-5*H*-furo[3,2-*g*]chromene-6-carbaldehyde (**2f**):

Yellow solid (0.30 g, 60%), R*_f_* (toluene-ethyl acetate) 0.63, mp. 228–229 °C; max (ATR) 519, 651, 790, 833, 1021, 1175, 1251, 1302, 1505, 1568, 1615, 1645, 1697, 2847, 2925, 3069, 3110 cm^−1^; δ_H_ (300 MHz, DMSO-*d*_6_) 3.75 (3H, s, OCH_3_), 7.00 (2H, d, *J* = 8.1 Hz, H-3′,5′), 7.90 (1H, s, H-3), 7.77 (2H, d, *J* = 8.4 Hz, H-2′,6′), 7.96 (1H, s, H-9), 8.23 (1H, s, H-7), 8.84 (1H, s, H-4), 10.06 (1H, s, –CHO); δ_C_ (75 MHz, DMSO-*d*_6_) 56.4, 101.1, 102.0, 115.7, 117.9 (2×C), 120.1, 122.3, 127.8, 129.9, 154.3, 157.9, 159.9, 161.5, 164.3, 176.1, 189.5; HRMS (ES^+^): *m*/*z* [M + H]^+^ calc. for C_19_H_12_O_5_: 321.0763; found 321.0773.

2-(3,5-Dimethoxyphenyl)-5-oxo-5*H*-furo[3,2-*g*]chromene-6-carbaldehyde (**2g**):

Yellow solid (0.39 g, 71%), R*_f_* (toluene-ethyl acetate) 0.52, mp. 236–237 °C; ν_max_ (ATR) 575, 602, 669, 791, 863, 1053, 1363, 1581, 1631, 1673, 2896, 2938 cm^−1^; δ_H_(300 MHz, DMSO-*d*_6_) 3.81 (6H, s, -OCH_3_), 6.55 (1H, t, *J* = 1.8 Hz, H-4′), 7.04 (2H, d, *J* = 1.8 Hz, H-2′,6′), 7.66 (1H, s, H-3), 8.07 (1H, s, H-9), 8.34 (1H, s, H-7), 8.91 (1H, s, H-4), 10.12 (1H, s, –CHO); δ_C_ (75 MHz, DMSO-*d*_6_) 56.0 (2×C), 101.7, 103.2, 118.3, 119.6, 121.9, 128.1, 128.8, 129.2, 129.3, 129.8, 131.9, 134.8, 154.1, 157.4, 157.8, 163.9, 175.5, 188.9; HRMS (ES^+^): *m*/*z* [M + H]^+^ calc. for C_20_H_13_O_6_ 350.0788; found 350.0790.

5-Oxo-2-(*p*-tolyl)-5*H*-furo[3,2-*g*]chromene-6-carbaldehyde (**2h**):

Yellow solid (0.36 g, 76%), R*_f_* (toluene-ethyl acetate) 0.52, mp. 214–215 °C; ν_max_ (ATR) 499, 582, 777, 831, 1025, 1175, 1251, 1305, 1505, 1572, 1606, 1650, 2858, 3059 cm^−1^; δ_H_(300 MHz, DMSO-*d*_6_) ; 2.36 (3H, s, –CH_3_), 7.33 (2H, d, *J* = 8.1 Hz, H-3′,5′), 7.57 (1H, s, H-3), 7.83 (2H, d, *J* = 7.5 Hz, H-2′,6′), 8.10 (1H, s, H-9), 8.38 (1H, s, H-7), 8.95 (1H, s, H-4), 10.15 (1H, s, –CHO); δ_C_ (75 MHz, DMSO-*d*_6_) 21.5, 101.5, 117.7, 119.6, 121.7, 125.5, 126.5, 129.1, 129.4, 130.3, 140.1, 153.9, 157.4, 159.3, 163.9, 175.6, 189.9; HRMS (ES^+^): *m*/*z* [M + H]^+^ calc. for C_19_H_11_O_4_ 305.0814; found 305.0810.

2-(Cyclohex-1-en-1-yl)-5-oxo-5*H*-furo[3,2-*g*]chromene-6-carbaldehyde (**2i**):

Yellow solid (0.34 g, 74%), R*_f_* (toluene-ethyl acetate) 0.52, mp. 183–184 °C; ν_max_ (ATR) 467, 560, 764, 1223, 1312, 1418, 1573, 1621, 1651, 1702, 2924, 3080 cm^−1^; δ_H_ (300 MHz, DMSO-*d*_6_) 1.63 (2H, t, *J* = 1.5 Hz, –CH_2_), 1.73 (2H, t, *J* = 3.9 Hz, –CH_2_), 2.25 (2H, t, *J* = 3.6 Hz, –CH_2_), 2.39 (2H, t, *J* = 1.6 Hz, –CH_2_), 6.64 (1H, t, *J* = 3.9 Hz, –CH), 7.12 (1H, s, H-3), 7.72 (1H, s, H-9), 8.00 (1H, s, H-8), 8.96 (1H, s, H-5), 10.15 (1H, s, –CHO); δ_C_ (75 MHz, DMSO-*d*_6_) 21.9, 22.1, 24.6, 25.4, 101.0, 117.5, 119.5, 121.4, 126.8, 128.7, 128.9, 134.3 153.9, 157.2, 160.3, 163.7, 175.5, 189.0; HRMS (ES^+^): *m*/*z* [M + H]^+^ calc. for C_18_H_14_O_4_: 294.0889; found 294.0892.

### 3.4. Typical Procedure for the Synthesis of Hydrazones ***3a***–***i*** from ***2a***–***i***

A stirred mixture of 3a (0.20 g, 0.63 mmol) and pyridine (0.05 g, 0.63 mmol) in EtOH (15 mL) was treated with 4-trifluoromethylphenyhydrazine (0.13 g, 0.76 mmol). The mixture was heated under reflux for 6 h and then poured onto crush ice. The precipitate was filtered off and purified by silica gel column chromatography to afford compound 3a as a brown solid. Compounds **3b**–**i** were prepared in this fashion.

2-Phenyl-6-[(4-trifluoromethylphenyl)hydrazonomethyl]furo[3,2-*g*]chromen-5-one (**3a**):

Brown solid (0.18 g, 61%), R*_f_* (ethyl acetate-hexane) 0.65, mp. 254–255 °C; max (ATR) 418, 469, 596, 795, 830, 1062, 1102, 1209, 1327, 1573, 1606, 1641, 2361, 3041, 3266 cm^−1^; δ_H_ (300 MHz, DMSO-*d*_6_) 7.18 (2H, d, *J* = 7.5 Hz, H-3″,5″), 7.40 7.60 (5H, m, Ar), 7.63 (1H, s, H-3), 7.85–7.93 (2H, m, Ar), 8.03 (1H, s, H-7), 8.07 (1H, s, H-8), 8.36 (1H, s, H-4), 8.84 (1H, s, –CH=N), 10.87 (1H, s, NH); δ_C_ (75 MHz, DMSO-*d*_6_) 99.7, 110.9, 112.2, 118.7, 119.2 (q, ^2^*J* = 25.2 Hz), 120.0, 121.7, 123.7, 125.4 (q, ^1^*J* = 269.1 Hz), 126.9, 127.2, 129.3, 129.6, 130.0, 131.1, 148.4 150.4, 152.7, 157.2, 157.6, 175.1; δ_F_ (282 MHz, DMSO-*d_6_*) -59.3; HRMS (ES^+^): *m*/*z* [M + H]^+^ calc. for C_25_H_16_ N_2_O_3_F_3_: 449.1113; found. 449.1117.

2-(3-Fluorophenyl)-6-[(4-trifluoromethylphenyl)hydrazonomethyl]furo[3,2-*g*]chromen-5-one (**3b**):

Yellow solid (0.17 g, 59%), R*_f_* (ethyl acetate-hexane) 0.61, mp. 250–251 °C; ν_max_ (ATR) 469, 595, 627, 781, 830, 1058, 1100, 1288, 1321, 1603, 1640, 2361, 3040, 3263 cm^−1^; δ_H_ (300 MHz, DMSO-*d*_6_) 7.13 (2H, d, *J* = 7.2 Hz, H-3″,5″), 7.28 (1H, t, *J* = 7.2 Hz, H-5′), 7.51 (2H, d, *J* = 7.5 Hz, H-2″,6″), 7.53 (1H, s, H-7), 7.18 (1H, s, H-9), 7.74 (2H, d, *J* = 13.5 Hz, H-4′), 8.01 (2H, d, *J* = 12.3 Hz, H-4′), 8.37 (1H, s, H-4), 8.84 (1H, s, –CH = N), 10.87 (1H, s, NH); δ_C_ (75 MHz, DMSO-*d*_6_) 101.1, 103.9, 112.1, 112.2 (d, ^2^*J*_CF_ = 23.75 Hz), 116.8 (d, ^2^*J*_CF_ = 20.8 Hz), 118.2, 118.7 (q, ^2^*J* = 46.5 Hz), 121.6, 125.9 (q, ^1^*J* = 262.6 Hz), 127.0 (d, ^4^*J*_CF_ = 3.87 Hz), 127.3 (d, ^3^*J*_CF_ = 3.75 Hz), 128.1, 131.2, 131.9 (d, ^3^*J*_CF_ = 8.5 Hz), 133.0, 143.6, 148.5, 153.6, 154.4, 157.2 (d, ^3^*J*_CF_ = 8.6 Hz), 163.0 (d, ^1^*J*_CF_ = 242.8 Hz), 175.5; δ_F_ (282 MHz, DMSO-*d_6_*) -112.1 (1F, d, *J* = 5.9 Hz,), -59.4; HRMS (ES^+^): *m*/*z* [M + H]^+^ calc. for C_25_H_15_N_2_O_3_F_4_: 467.1019; found 467.1009..

2-(4-Fluorophenyl)-6-[(4-trifluoromethylphenyl)hydrazonomethyl]furo[3,2-*g*]chromen-5-one (**3c**):

Yellow solid (0.19 g, 68%), R*_f_* (ethyl acetate-hexane) 0.65, mp. 252–253 °C; ν_max_ (ATR) 516, 600, 657, 796, 837, 1064, 1111, 1160, 1236, 1305, 1327, 1504, 1620, 1643, 2360, 3077, 3259 cm^−1^; δ_H_ (300 MHz, DMSO-*d*_6_) 7.17 (2H, d, *J* = 8.7 Hz, H-3″,5″), 7.34 (2H, t, *J* = 8.7 Hz, H-3′,5′), 7.51 (2H, d, *J* = 8.7 Hz, H-2″,6″), 7.60 (1H, s, H-7), 8.00 (2H, dd, *J*_HH_ = 3.3 Hz and *J*_HF_ = 6.9 Hz, H-2′,6′), 8.06 (1H, s, H-9), 8.35 (1H, s, H-4), 8.85 (1H, s, –CH = N), 10.86 (1H, s, -NH); δ_C_ (75 MHz, DMSO-*d*_6_) 99.4, 102.7, 116.9 (d, ^2^*J*_CF_ = 22.87 Hz), 119.2 (q, ^2^*J* = 35.3 Hz), 120.8, 121.9, 122.0 (d, ^4^*J*_CF_ = 2.25 Hz), 124.3, 124.7 (d, ^3^*J*_CF_ = 8.6 Hz), 125.3 (q, ^1^*J* = 270.2 Hz), 129.2, 129.3, 129.6, 129.9, 131.9, 132.5, 135.9, 136.0, 143.0, 155.8, 158.2, 158.4, 161.4 (d, ^1^*J*_CF_ = 242.8 Hz), 175.4; δ_F_ (282 MHz, DMSO-*d_6_*) –111.0 (1F, ddd, *J* = 2.8, 9.0, 13.8), –59.3; HRMS (ES^+^): *m*/*z* [M + H]^+^ calc. for C_25_H_15_N_2_O_3_F_4_: 467.1019; found 467.1014.

2-(3-Chlorophenyl)-6-[(4-trifluoromethylphenyl)hydrazonomethyl]furo[3,2-*g*]chromen-5-one (**3d**):

Yellow solid (0.18 g, 65%), R*_f_* (ethyl acetate-hexane) 0.62, mp. 239–240 °C; ν_max_ (ATR) 443, 508, 796, 826, 1052, 1085, 1222, 1320, 1602, 1640, 2353, 3035, 3267 cm^−1^; δ_H_ (300 MHz, DMSO-*d*_6_) 7.14 (2H, d, *J* = 8.1 Hz, H-3″,5″), 7.26 (1H, t, *J* = 9.0 Hz, H-3′,5′) 7.50 (2H, d, *J* = 8.1 Hz, H-3′,5′), 7.53 (1H, s, H-3), 7.69–7.77 (3H, m, H-2″,6″), 7.80 (1H, s, H-7), 8.04 (1H, s, H-9), 8.34 (1H, s, H-4), 8.81 (1H, s, –CH = N), 10.84 (1H, s, -NH); δ_C_ (75 MHz, DMSO-*d*_6_) 102.5, 112.2, 117.0, 118.3, 120.5 (q, ^2^*J* = 64.30 Hz), 125.5, 125.8 (q, ^1^*J* = 260.0 Hz), 126.8, 127.4, 128.4, 129.3, 129.7, 130.1, 131.3, 131.6, 143.6, 148.5, 153.4, 154.2, 157.3, 159.6, 175.6; δ_F_ (282 MHz, DMSO-*d_6_*) –59.4; HRMS (ES^+^): *m*/*z* [M + H]^+^ calc. for C_25_H_15_N_2_O_3_F_3_^35^Cl: 483.0723; found 483.0720.

2-(4-Chlorophenyl)-6-[(4-trifluoromethylphenyl)hydrazonomethyl]furo[3,2-*g*]chromen-5-one (**3e**):

Yellow solid (0.17 g, 60%), R*_f_* (ethyl acetate-hexane) 0.63, mp. 230–231 °C; ν_max_ (ATR) 437, 503, 594, 796, 827, 1062, 1095, 1224, 1325, 1606, 1642, 2359, 3045, 3262 cm^−1^; δ_H_ (300 MHz, DMSO-*d*_6_) 7.15 (2H, d, *J* = 8.0 Hz, H-3″,5″), 7.50 (2H, d, *J* = 8.0 Hz, H-3′,5′), 7.56 (2H, d, *J* = 8.0 Hz, H-2″,6″), 7.62 (1H, s, H-3), 7.91 (2H, d, *J* = 8.0 Hz, H-2′,6′), 7.96 (1H, s, H-7), 8.03 (1H, s, H-9), 8.33 (1H, s, H-4), 8.80 (1H, s, –CH=N), 10.83 (1H, s, –NH); δ_C_ (75 MHz, DMSO-*d*_6_) 101.0, 103.1, 112.1, 118.0, 118.5 119.0 (q, ^2^*J* = 31.4 Hz), 120.6, 126.9, 127.1, 127.0 (q, ^2^*J* = 276.1 Hz), 129.5, 129.6, 129.7 131.2, 134.6, 148.4, 153.4, 154.2, 157.2, 157.4, 175.4; δ_F_ (282 MHz, DMSO-*d_6_*) –59.3; HRMS (ES^+^): *m*/*z* [M + H]^+^ calc. for C_25_H_15_N_2_O_3_F_3_^35^Cl: 483.0723; found 483.0731.

2-(4-Methoxyphenyl)-6-[(4-trifluoromethylphenyl)hydrazonomethyl]furo[3,2-*g*]chromen-5-one (**3f**):

Brown solid (0.24 g, 78%), R*_f_* (ethyl acetate-hexane) 0.58, mp. 233–234 °C; max (ATR) 510, 632, 792, 831, 1064, 1109, 1172, 1222, 1258, 1299, 1473, 1506, 1568, 1615, 1640, 1698, 2840, 2934, 3075, 3258 cm^−1 1^H (300 MHz, DMSO-*d*_6_) 3.81 (3H, s, -OCH_3_), 7.05 (2H, d, *J* = 8.4 Hz, H-3″,5″), 7.77 (2H, d, *J* = 8.1 Hz, H-3′,5′), 7.45 (1H, s, H-7), 7.51 (2H, d, *J* = 8.1 Hz, H-2″,6″), 7.86 (2H, d, *J* = 8.1 Hz, H-2′,6′), 7.97 (1H, s, 8-H), 8.07 (1H, s, 5-H), 8.30 (1H, s, 2-H), 8.84 (1H, s,), 10.87 (1H, s,); ^13^C-NMR (75 MHz, DMSO-*d*_6_) 55.8, 100.5, 100.8, 112.1, 115.1, 117.1, 118.4 (q, ^2^*J* = 36.4 Hz), 120.5, 121.9, 125.3 (q, ^1^*J* = 272.3 Hz), 127.0, 127.1, 127.9, 128.7, 148.5, 153.5, 153.9, 157.2, 158.9, 160.8, 175.5; δ_F_ (282 MHz, DMSO-*d_6_*) –59.3; HRMS (ES^+^): *m*/*z* [M + H]^+^ calc. for C_26_H_18_N_2_O_4_F_3_ 479.1219; found 479.1211.

2-(3,5-Dimethoxyphenyl)-6-[(4-trifluoromethylphenyl)hydrazonomethyl]furo[3,2-*g*]chromen-5-one (**3g**):

Yellow solid (1.77 g, 63%), R*_f_* (ethyl acetate-hexane) 0.52, mp.166–167 °C; ν_max_ (ATR) 680, 793, 831, 1063, 1110,1155, 1205,1322, 1456, 1600, 1637, 2934, 3255 cm^−1^; δ_H_ (300 MHz, DMSO-*d*_6_) 3.81 (6H, s, –OCH_3_), 6.53 (1H, t, *J* = 1.8 Hz, H-4′), 7.01 (2H, d, *J* = 1.8 Hz, H-2′,6′), 7.21 (1H, s, H-3), 7.46 (1H, s, H-9), 7.91 (2H, d, *J* = 8.7 Hz, H-2″,6″), 8.06 (1H, s, H-7), 8.21 (2H, d, *J* = 8.7 Hz, H-3″,5″) 8.31 (1H, s, H-4), 9.30 (1H, s, –CH=N), 11.36 (1H, s, -NH); δ_C_ (75 MHz, DMSO-*d*_6_) 56.0 (2×C), 100.5, 102.2, 103.4, 110.9, 112.3, 119.2 (q, ^2^*J* = 36.0 Hz), 120.1, 121.9, 125.8, 127.0, 127.6, 127.9 (q, ^1^*J* = 265.5 Hz), 129.4, 131.1, 131.2, 148.4, 150.5, 152.6, 157.1, 157.6, 161.4, 175.1; δ_F_ (282 MHz, DMSO-*d_6_*) –59.3; HRMS (ES^+^): *m*/*z* [M + H]^+^ calc. for C_27_H_20_N_2_O_5_F_3_ 509.1324; found 509.1317.

2-(4-*p*-Tolyl)-6-[(4-trifluoromethylphenyl)hydrazonomethyl]furo[3,2-*g*]chromen-5-one (**3h**):

Yellow solid (0.16 g, 74%), R*_f_* (ethyl acetate-hexane) 0.68, mp. 258–259 °C; max (ATR) 490, 774, 835, 1064, 1119, 1156, 1210, 1326, 1418, 1597, 1616, 1632, 2926, 3259 cm^−1 1^H (300 MHz, DMSO-*d*_6_) 2.31 (3H, s, –CH_3_), 7.16 (2H, d, *J* = 8.7 Hz, H-3″,5″), 7.33 (2H, d, *J* = 8.7 Hz, H-3′,5′), 7.50 (1H, s), 7.52 (2H, d, *J* = 8.1 Hz, H-2″,6″), 7.83 (2H, d, *J* = 8.1 Hz, H-2′,6′), 8.00 (1H, s, 8-H), 8.07 (1H, s, 5-H), 8.33 (1H, s, 2-H), 8.86 (1H, s,), 10.90 (1H, s,); δ_C_ (75 MHz, DMSO-*d*_6_) 31.2, 104.2, 110.8, 112.2, 119.4 (q, ^2^*J* = 32.1 Hz), 120.0, 120.2, 121.5, 124.2, 125.1, 125.4, 126.9, 127.6 (q, ^1^*J* = 273.8 Hz), 130.1, 131.2, 139.2, 143.6, 152.7, 155.9, 157.5, 158.9, 175.1; δ_F_ (282 MHz, DMSO-*d_6_*) –59.4; HRMS (ES^+^): *m*/*z* [M + H]^+^ calc. for C_26_H_18_N_2_O_3_F_3_; 460.1267; found. 463.1270.

2-(Cyclohex-1-en-1-yl)-6-((2-(4-(trifluoromethyl)phenyl)hydrazono)methyl)-5*H*-furo[3,2-*g*]chromen-5-one (**3i**):

Brown solid (1.90 g, 62%), R*_f_* (ethyl acetate-hexane) 0.67, mp. 232–233 °C; ν_max_ (ATR) 501, 597, 827, 1004, 1025, 1060, 1105, 1155, 1323, 1472, 1584, 1605, 1637, 1719, 2924, 3259 cm^−1^; δ_H_ (300 MHz, DMSO-*d*_6_) 1.65 (4H, t, *J* = 34.5 Hz –CH_2_), 2.27 (4H, t, *J* = 34.5 Hz, –CH_2_), 6.60 (1H, t, *J* = 3.9 Hz, –CH), 6.93 (1H, s, H-3), 7.16 (2H, d, *J* = 8.4 Hz, H-2″,6″), 7.52 (2H, d, *J* = 8.7 Hz, H-3″,5″) 7.88 (1H, s, H-9), 8.02 (1H, s, H-8), 8.26 (1H, s, H-5), 8.83 (1H, s, –CH=N), 10.87 (1H, s, –NH); δ_C_ (75 MHz, DMSO-*d*_6_) 21.9, 22.1, 24.7, 25.4, 29.4, 99.1, 100.5, 101.1, 112.3, 117.3, 118.6 (q, ^2^*J* = 38.2 Hz), 120.1, 122.7, 127.9 (q, ^1^*J* = 264.5 Hz), 126.1, 126.9, 128.4, 131.5, 132.9, 148.5, 153.5, 154.2, 160.0, 175.5; δ_F_ (282 MHz, DMSO-*d_6_*) –59.3; HRMS (ES^+^): *m*/*z* [M + H]^+^ calc. for C_25_H_20_N_2_O_3_F_3_: 453.1426; found 453.1424..

### 3.5. AChE and BChE Inhibitory Assays

768104 BL Recombinant human AChE (carrier-free) and RPC348Hu0 2_100 CLC Recombinant BChE (Type, cloud-clone corp.) were purchased through BIOCOM Africa (Pty) Ltd. (Biocom Africa, Pretoria, South Africa) and the anti-AChE and BChE activities were determined by spectrophotometric Ellman’s method [43]. The stock solutions of the test compounds (200 µM) were prepared in a 2:8 DMSO-water mixture (v/v) followed by dilution with 50 mM tris buffer (pH 7.7) to obtain final assay concentrations of 10, 25, 50 and 100 µM. The assays were conducted in triplicates using donepezil and galantamine as positive controls (10, 25, 50 and 100 µM), respectively. One microliter (1.0 µL) of the respective enzyme, 9.0 µL of the test compounds (various concentrations of **2b**, **2f**, **3b**, **3d**, **3f** and controls, respectively) and 70 µL of tris buffer were added into the wells of a 96-well plate. The mixtures were incubated for 15 min at RT. The reaction was initiated by adding of 10 µL of mixture solution of 5 mM 5,50-dithio-bis-nitrobenzoic acid (DTNB) in 50 mM tris buffer (pH 7.7) and 10 µL acetylthiocholine (ASCh; 5 mM) in 50 mM tris buffer, pH 7.7 into the wells containing the mixtures. The hydrolysis of ASCh was determined by monitoring the formation of the yellow 5-thio-2-nitrobenzoate anion resulting from reaction of DTNB with thiocholines (SCh). The mixtures were further incubated for 5 min at RT and absorbance measured at a wavelength of 405 nm using Varioskan flash microplate spectrophotometer (Thermo Scientific, Waltham, MA, USA). Inhibition of BChE was measured in the same manner as described for AChE. Percent of inhibition was calculated by the following equation:

(1) $$\mathrm{Inhibition}\;\mathrm{activity}\;\left(\%\right)=¼\frac{\left(\mathrm{Absorbance}\;\mathrm{of}\;\mathrm{control}-\mathrm{Absorbance}\;\mathrm{of}\;\mathrm{sample}\right)}{\mathrm{Absorbance}\;\mathrm{of}\;\mathrm{control}}\times 100.$$

The IC_50_ values were determined graphically from inhibition curves (inhibitor concentration and absorbance) using the graph pad prism.

### 3.6. In Vitro β-Secretase Inhibitory Assays for Compounds ***2b***, ***2f***, ***3b***, ***3d*** and ***3f***

The inhibitory properties of compounds **2b**, **2f**, **3b**, **3d** and **3f** on β-secretase were evaluated by a fluorescence resonance energy transfer (FRET) assay (Pan Vera) with a recombinant baculovirus-expressed β-secretase and a specific substrate (Rh-EVNLDAEFK-Quencher) according to manufacturer instructions. A mixture of human recombinant BACE-1 (1.0 U/mL), the substrate (75 µM in 50 mM ammonium bicarbonate) and test compound (various concentration of 2b, 2f, 3b, 3d, 3f and quercetin, respectively) dissolved in an assay buffer (50 mM sodium acetate, pH 4.5), was incubated for 60 min at 25 °C in a 96 well plate. The assays were performed in triplicates and the increase in fluorescence intensity produced by substrate was observed on a fluorescence microplate reader with an excitation wavelength of 545 nm and emission wavelength 590 nm. The inhibition ratio was calculated using the following equation:

(2) $$\mathrm{Inhibition}\;\left(\%\right)=\frac{1-\left(S-S_{0}\right)}{\left(C-C_{0}\right)}\times 100,$$

where C is the fluorescence of control (enzyme, assay buffer and substrate) after 60 min of incubation, C_0_ is the fluorescence of control at time 0, S is the fluorescence of tested samples (enzyme, sample solution, and substrate) after 60 min. of incubation and S_0_ the fluorescence of the tested samples at time 0.

### 3.7. Lipoxygenase Activity Assays

#### 3.7.1. LOX-15 (Soybean) Inhibitory Assay of Compounds **2b**, **2f**, **3b**, **3d** and **3f**

The assay was conducted as described in the literature [44] with some modification and each compound was tested in duplicate with quercetin used as a reference standard. The reagents were prepared according to the standard protocol (Lipoxygenase inhibitor screening assay kit, Item No. 760700. Cayman Chemicals, Ann Arbor, MI, USA). Stock solutions (200 µM) of tested samples were prepared in DMSO. The stock solutions were further diluted with 50 mM Tris buffer of pH 7.7 to obtain concentrations of 0.01, 0.1, 1, 10 and 100 µM. The cells of a 96-well plate where distributed as follows: two wells taken as blanks (100 µL 50 mM Tris buffer pH 7.7), two wells taken as positive controls (90 µL of 15-LO Standard and 10 µL of DMSO) and two wells as 100% initial activity wells (90 µL lipoxygenase enzyme solution and 10 µL tris buffer). Then 90 µL of lipoxygenase enzyme solution and 10 µL of each compound (**2b**, **2f**, **3b**, **3d**, **3f** and quercetin, respectively) from the above concentrations were added in the other wells of a 96-well plate. The 96-well plate was then incubated for 5 min at room temperature. The reaction was initiated by the addition of 10 µL of the substrate (arachidonic acid) to each well containing the mixtures. After shaking the 96-well plate on a shaker for 5 min, 100 µL of the chromogen (solution of equal volume developing reagent 1 and 2) was added to each well to stop enzyme catalysis and develop the reaction. The plate was again shaken for 5 min and the absorbance read at 490 nm in a Varioskan flash microplate spectrophotometer reader. The inhibitory concentration was expressed as a percentage using the formula below.

(3) $$\mathrm{Inhibitory}\;\mathrm{concentration}\;=\frac{A_{\mathrm{Initial}\;\mathrm{activity}}-\left(A_{\mathrm{Sample}}-A_{\mathrm{Blank}\;\mathrm{sample}}\right)}{A_{\mathrm{Initial}\;\mathrm{activity}}}\times 100.$$

A_Sample_ is the absorbance of the reaction mixture of the test sample, A_Blank_ sample is the absorbance of the reaction mixture containing all reagents except enzyme and A_Initial activity_ is the absorbance of 100% initial activity.

#### 3.7.2. Human LOX-5 Inhibitory Assay of Compound **2f**

The reagents were prepared according to the Lipoxygenase inhibitor screening kit (Item No. K980; BioVision, Milpitas Blvd, Milpitas, CA, USA) standard protocol. Stock solution (200 µM) of **2f** was prepared in DMSO and was further diluted with 50 mM Tris HCl buffer (pH 7.7) to obtain final concentrations of 0.001, 0.1, 1, 5 and 10 µM. The compound and positive controls (quercetin and zileuton) were tested in duplicate for this assay. The cells of a 96-well plate where distributed as follows: two wells were taken as blanks (100 µL of assay buffer), two wells enzyme controls (90 µL of 15-LO Standard and 10 µL of tris buffer), two wells as 100% initial activity wells (90 µL and 10 µL DMSO) and three wells as enzyme control (40 µL). Two microliters (2 µL) of the test compound (2f, quercetin or zileuton, respectively) and 38 µL of LOX assay buffer were added to the other wells of the 96-well plate in duplicates. Forty µL of the reaction mixture (prepared by mixing thoroughly 36 µL of LOX assay buffer, 2 µL of LOX probe and 2 µL of 5-LOX enzyme) were added to each well and incubated for 10 min. at RT. The DMSO concentration in each reaction well was 1% to prevent enzyme structural deformation and therefore, reduced activity. The reaction was initiated by adding 20 µL of LOX substrate solution (prepared by diluting LOX substrate in a ratio 1:25 with assay buffer) to each well and absorbance read at 234 nm in a Varioskan flash microplate spectrophotometer reader. The percentage inhibition (%) was calculated using the equation below.

(4) $$\%\;\mathrm{Inhibition}=\frac{\mathrm{Control}-\mathrm{Test}}{\mathrm{Control}}\times 100.$$

### 3.8. Kinetic Studies against ChEs and β-Secretase

#### 3.8.1. Kinetic studies of **2b**, **2f**, **3b**, **3d** and **3f** Against ChEs

Compounds **2f**, **2b**, **2f**, **3b**, **3d** and **3f** were selected for the kinetic studies with substrate concentrations 0.1, 0.5, 2. 5 and 5 µM for ChEs following procedures described in our previous investigation [45]. The Lineweaver–Burk plots (plots of the inverse of velocity (1/v) against the inverse of the substrate concentration (1/[S])) were used to ascertain the mode of inhibition of these compounds. The Dixon plots of 1/v against concentration of inhibitor at each concentration of substrate were used to determine their inhibitor constants (K*_i_*).

#### 3.8.2. Kinetic Studies of **2f** Against β-Secretase

Compound **2f** was selected for the kinetic studies with substrate concentrations 150, 300 and 450 nM for β-secretase following procedure described in our previous investigation [45]. The Lineweaver–Burk plot (plot of the inverse of velocity (1/v) against the inverse of the substrate concentration (1/[S])) was used to ascertain the mode of inhibition of this compound. The plot of 1/v against concentration of inhibitor at each concentration of substrate (the Dixon plot) was used to determine the inhibitor constant (K*_i_*).

### 3.9. 2.2-Diphenyl-1-Picrylhydrazyl (DPPH) Radical Scavenging Activity Assay

DPPH radical scavenging activity of compounds **2b**, **2f**, **3b**, **3d** and **3f** was evaluated following the literature method [46]. Ascorbic acid (Sigma Aldrich, Saint Louis, Missouri, USA) was used as a positive control. The test compounds and controls at various concentrations ranging from (0 µM to 40 µM) in DMSO were mixed with a solution of DPPH (0.20 mM) in methanol. The mixtures were incubated in the dark for 45 min and the absorbances were recorded at 512 nm using Varioskan flash microplate spectrophotometer reader. All tests and analyses were run in triplicate and averaged. The inhibition was calculated in terms of percentage using the formula below:

(5) $$\mathrm{DPPH}\;\mathrm{radical}\;\mathrm{scavenged}\;\left(\%\right)=\frac{\mathrm{AbC}-\mathrm{AbS}}{\mathrm{AbC}}\times 100,$$

where AbC is absorbance of control and AbS the absorbance of the test sample. A graph of percentage inhibition of free radical activity was plotted against concentration of the sample and the IC_50_ (compound concentration required to reduce the absorbance of the DPPH control solution by 50%) was obtained from the graph.

### 3.10. Molecular Modeling of Compounds ***2b***, ***2f***, ***3b***, ***3d*** and ***3f***

The CDOCKER module of Discovery studio software (version 17.1.0.16143; Accelrys, San Diego, CA, USA) was used to explore the hypothetical interactions of the title compounds with AChE, BChE, β-secretase and LOX-5. Protein Data Bank (PDB) structures used were as follows: 1GQR for AChE, 1P0I for BChE, 3IXJ for β-Secretase and 3O8Y for LOX-5. The proteins structures were prepared prior to docking using default settings of Discovery Studio except that co-crystalized ligands were removed. The compounds were drawn in discovery studio then prepared using default parameters prior to docking. The binding sites used to dock compounds represented co-crystalized ligand or substrate locations as identified by Discovery Studio software. The x, y and z coordinates for the binding spheres were as follows: 1GQR (3.05658, 65.714 and 65.7878); 1POI (135.617, 115.53 and 38.362); 3IXJ (−1.47841, 15.4838 and 32.8487) and 3O8Y (−7.04812, 75.138 and 20.4803). The best scoring pose (optimal CDOCKER and CDOCKER interaction energies), without unfavorable interactions, was selected and represented as 2D plots using Discovery Studio. Binding energies were calculated for the best scoring pose using the calculate binding energy tool with default settings.

### 3.11. Cytotoxicity Studies of ***2f*** Against MCF-7 and Hek293-T Cells

Briefly, the cells were seeded in 96-well plate at a density of 20 × 103 cells per well and then incubated at 37 °C in 5% CO_2_ to allow cell attachment. The medium was removed and replaced with fresh medium containing various concentrations of **2f** and doxorubicin hydrochloride (10, 25, 50, 100 and 200 μM) and incubated for 24 h. An equal volume of medium was used as the control treatment. After treatment for 24 h, 10 μL MTT (5 mg/mL) was added to each well and the plate was further incubated for 4 h later. The supernatant was removed and 100 μL of DMSO was added to each well to dissolve the resulting formazan crystals. The absorbance was read at 570 nm using the Varioskan flash microplate spectrophotometer reader. The percentages of cell viability were used to determine the IC_50_ values.

## 4. Conclusions

A combination of 3-fluorophenyl- group at the C-2 position of the furan moiety and a 6-carbaldehyde functionality resulted in significant inhibitory effect of **2b** against cholinesterases and moderate activity against β-secretase and lipoxygenase-15 accompanied by increased anti-oxidant effect. Replacement of the 6-carbaldehyde group of **2b** with 6-hydrazono functionality in **3b**, on the other hand, reduced the anticholinesterase, anti-lipoxygenase and anti-oxidant activities, but enhanced activity against β-secretase. The presence of fluorine atom on the para position of the phenyl substituent appears not favorable for anticholinesterase activity for the title compounds. Increased and reverse trend in inhibitory effect against AChE and BChE were observed for the 4-methoxyphenyl substituted furanochromenone-6-carbaldehyde **2f** and its hydrazono derivative **3f**. Although active against cholinesterases, the presence of the hydrazono functionality in **3f** resulted in reduced inhibitory effect against β-secretase and lipoxygenase-15, as well as reduced anti-oxidant activity. A combination of 4-methoxyphenyl and carbaldehyde groups on the furochromenone scaffold makes compound **2f** capable of controlling multiple targets involved in this disease at the same time, namely, cholinesterases (AChE and BChE), β-secretase and lipoxygenases (LOX-5/15) including free radical scavenging potential. This compound has also been found to be moderately cytotoxic against the MCF-7 cancer cell line with no toxicity towards the Hek293-T cells when compared to the anticancer drug, doxorubicin. Compound **2f** represents a potential candidate for pharmacological application in the field of neurodegenerative and inflammatory diseases as well as cancer therapy. Further studies are required to clarify the underlying mechanisms of cancer cell death (e.g., apoptosis vs. necrosis) and to determine if **2f** has the same effects on these enzyme targets in vivo.

## Figures and Tables

**Table 1:** Substitution pattern and percentage yields of **2a**–**i** and **3a**–**i**.

Entry	R	%Yield 2a–i	%Yield 3a–i
1	C_6_H_5_-	61 (**2a**)	61 (**3a**)
2	3-FC_6_H_4_-	64 (**2b**)	59 (**3b**)
3	4-FC_6_H_4_-	71 (**2c**)	68 (**3c**)
4	3-ClC_6_H_4_-	69 (**2d**)	65 (**3d**)
5	4-ClC_6_H_4_-	66 (**2e**)	60 (**3e**)
6	4-MeOC_6_H_4_-	60 (**2f**)	78 (**3f**)
7	3,5-MeO(C_6_H_3_)-	71 (**2g**)	63 (**3g**)
8	4-MeC_6_H_4_-	76 (**2h**)	74 (**3h**)
9	-Cyclohex-1-en-1-yl	74 (**2i**)	62 (**3i**)

**Table 2:** AChE and BChE inhibitory activities of compounds **2a**–**i**.

Compound	IC_50_ (µM)		SI	
	AChE	BChE	BChE/AChE	AChE/BChE
**2a**	28.6 ± 0.01	12.3 ± 0.02	0.43	2.33
**2b**	5.4 ± 0.05	6.4 ± 0.04	1.19	0.84
**2c**	15.3 ± 0.01	15.7 ± 0.02	1.03	0.97
**2d**	33.4 ± 0.03	21.6 ± 0.01	0.65	1.54
**2e**	21.8 ± 0.03	14 ± 0.04	0.62	1.61
**2f**	9.4 ± 0.01	5.1 ± 0.02	0.54	1.85
**2g**	10.8 ± 0.02	41.2 ± 0.03	3.81	0.26
**2h**	12.9 ± 0.01	28.1 ± 0.03	2.18	0.46
**2i**	22.6 ± 0.03	34.8 ± 0.02	1.54	0.65
Donepezil	0.02 ± 0.03	6.11 ± 0.01	305.5	0.003
Galantamine	0.01 ± 0.01	0.16 ± 0.02	16.00	0.06

Selectivity index (SI) means IC_50_ (BChE)/IC_50_ (AChE) or (AChE)/IC_50_ (BChE).

**Table 3:** AChE and BChE inhibitory activities of compounds 3a–i.

Compound	IC_50_ (µM)		SI	
	AChE	BChE	BChE/AChE	AChE/BChE
**3a**	18.4 ± 0.01	26.3 ± 0.02	1.43	0.70
**3b**	7.6 ± 0.03	11.2 ± 0.02	1.47	0.68
**3c**	21.2 ± 0.01	14.1 ± 0.01	0.67	1.49
**3d**	18.3 ± 0.04	7.2 ± 0.03	0.39	2.56
**3e**	12.9 ± 0.02	24.6 ± 0.02	1.91	0.52
**3f**	5.4 ± 0.02	9.6 ± 0.05	1.78	0.56
**3g**	34.9 ± 0.01	31.2 ± 0.04	0.89	1.12
**3h**	10.5 ± 0.03	35.4 ± 0.015	3.37	0.30
**3i**	29.8 ± 0.01	30.9 ± 0.02	1.04	0.96
Donepezil	0.02 ± 0.03	6.11 ± 0.01	305.50	0.003
Galantamine	0.01 ± 0.01	0.16 ± 0.02	16.00	0.06

**Table 4:** β-Secretase inhibitory activity of compounds **2b**, **2f**, **3b**, **3d** and **3f**.

Compound	IC_50_ (µM) BACE-1
**2b**	21.6 ± 0.01
**2f**	15.4 ± 0.02
**3b**	17.3 ± 0.03
**3d**	32.8 ± 0.02
**3f**	25.3 ± 0.04
Quercetin	12.5 ± 0.01

**Table 5:** Evaluation of **2b**, **2f**, **3b**, **3d**, **3f** and **3h** against lipoxygenases (LOX-5 and LOX-15).

Compound		IC_50_ (μM)	
	LOX-15	LOX-5	SI
**2b**	16.3 ± 0.01	-	-
**2f**	9.6 ± 0.01	15.3 ± 0.01	0.63
**3b**	21.6 ± 0.04	-	-
**3d**	19.8 ± 0.02	-	-
**3f**	26.5 ± 0.05	-	-
Quercetin	3.4 ± 0.01	10.2 ± 0.01	0.33
Zileuton	-	11.8 ± 0.02	-

**Table 6:** Evaluation of **2b**, **2f**, **3b**, **3d** and **3f** for antioxidant activity.

Compound	IC_50_ (μM)
	DPPH
**2b**	7.4 ± 0.01
**2f**	18.1 ± 0.03
**3b**	21.8 ± 0.02
**3d**	20.9 ± 0.03
**3f**	23.9 ± 0.04
Ascorbic acid	4.8 ± 0.01

**Table 7:** Calculated binding free energy (BE) values **2b**, **2f**, **3b**, **3d** and **3f**.

Compound	BE (kcal/mol)
**2b**	−56.65
**2f**	−66.76
**3b**	−74.98
**3d**	−67.49
**3f**	−85.00
Donepezil	−73.50

**Table 8:** Calculated binding energy values for **2b**, **2f**, **3b**, **3d** and **3f**.

Compound	BE (kcal/mol)
**2b**	−60.48
**2f**	−62.80
**3b**	−37.07
**3d**	−49.14
**3f**	−62.59
Donepezil	−39.00

## Supplementary Materials

Supplementary materials can be found at https://www.mdpi.com/1422-0067/20/21/5451/s1. Copies of ^1^H- and ^13^C-NMR spectra of compounds **1**–**3** (Figure S1), Lineweaver-Burk and Dixon plots for AChE (Figure S2.1), BChE (Figure S2.2) and BACE-1 (Figure S2.3); the docking poses of donepezil, quercetin and zileuton and their interaction with AChE (Figure S3) & BChE (Figure S4), BACE-1 (Figure S5) and LOX-5 (Figure S6) protein residues and graphs for evaluation of cytotoxicity of **2f** and doxorubicin against MCF-7 cells (Figure S7).

## References

[1] Gammon K. (2014). Neurodegenerative disease: Brain windfall. Nature.

[2] Durães F., Pinto M., Sousa E. (2018). Old drugs as new treatments for neurodegenerative diseases. Pharmaceuticals.

[3] Emmerzaal T.L., Kiliaan A.J., Gustafson D.R. (2015). 2003–2013: A decade of body mass index, Alzheimer’s disease, and dementia. J. Alzheimer’s Dis..

[4] Marco-Contelles J., Unzeta M., Bolea I., Esteban G., Ramsay R.R., Romero A., Martinez-Murillo R., Carreiras M.C., Ismaili L. (2016). Ass234, as a new multi-target directed propargylamine for Alzheimer’s disease therapy. Front. Neurosci..

[5] Verdile G., Fuller S.J., Martins R.N. (2015). The role of type 2 diabetes in neurodegeneration. Neurobiol. Dis..

[6] Kan M.J., Lee J.E., Wilson J.G., Everhart A.L., Brown C.M., Hoofnagle A.N., Jansen M., Vitek M.P., Gunn M.D., Colton C.A. (2015). Arginine deprivation and immune suppression in a mouse model of Alzheimer’s disease. J. Neurosci..

[7] Van Eldik L.J., Carrillo M.C., Cole P.E., Feuerbach D., Greenberg B.D., Hendrix J.A., Kennedy M., Kozauer N., Margolin R.A., Molinuevo J.L. (2016). The roles of inflammation and immune mechanisms in Alzheimer’s disease. Alzheimer’s Dement. Transl. Res. Clin. Interv..

[8] Zhang F., Jiang L. (2015). Neuroinflammation in Alzheimer’s disease. Neuropsychiatr. Dis. Treat..

[9] Pratico D., Zhukareva V., Yao Y., Uryu K., Funk C.D., Lawson J.A., Trojanowski J.Q., Lee V.M.-Y. (2004). 12/15-Lipoxygenase is increased in Alzheimer’s disease: Possible involvement in brain oxidative stress. Am. J. Pathol..

[10] Ikonomovic M.D., Abrahamson E.E., Uz T., Manev H., DeKosky S.T. (2008). Increased 5-lipoxygenase immunoreactivity in the hippocampus of patients with Alzheimer’s disease. J. Histochem. Cytochem..

[11] Christen Y. (2000). Oxidative stress and Alzheimer disease. Am. J. Clin. Nutr..

[12] Darvesh S., Hopkins D., Geula A.C. (2003). Neurobiology of butyrylcholinesterase. Nat. Rev. Neurosci..

[13] Colovic M.B., Krstic D.Z., Lazarevic-Pasti T.D., Bondzic A.M., Vasic V.M. (2013). Acetylcholinesterase inhibitors: Pharmacology and toxicology. Curr. Neuropharmacol..

[14] Morphy R., Rankovic Z. (2005). Designed multiple ligands. An emerging drug discovery paradigm. J. Med. Chem..

[15] Bajda M., Guzior N., Ignasik M., Malawska B. (2011). Multi-target-directed ligands in Alzheimer’s disease treatment. Curr. Med. Chem..

[16] Reis J., Gaspar A., Milhazes N., Borges F. (2017). Chromone as a privileged scaffold in drug discovery: Recent advances. J. Med. Chem..

[17] Ando K., Kawamura Y., Akai Y., Kunitomo J., Yokomizo T., Yamashita M., Ohta S., Ohishi T. (2008). Preparation of 2-, 3-, 4- and 7-(2-alkylcarbamoyl-1-alkylvinyl)benzo[*b*]furans and their BLT_1_ and/or BLT_2_ inhibitory activities. Org. Biomol. Chem..

[18] Galal S.A., AbdEl-All A.S., Abdallah M.M., El Diwani H.I. (2009). Synthesis of potent antitumor and antiviral benzofuran derivatives. Bioorg. Med. Chem. Lett..

[19] Negavi R.J., Dighe S.N., Dighe S.N. (2015). Biological and medicinal significance of benzofuran. Eur. J. Med. Chem..

[20] Namdanung U., Athipornchai A., Khammee T., Kuno M., Suksamrarn S. (2018). 2-Arylbenzofurans from *Artocarpus lakoocha* and methyl ether analogs with potent cholinesterase inhibitory activity. Eur. J. Med. Chem..

[21] Gaspar A., Matos M.J., Garrido J., Uriarte E., Borges F. (2014). Chromone: A valid scaffold in medicinal chemistry. Chem. Rev..

[22] Keri R.S., Budagumpi S., Pai R.K., Balakrishna R.G. (2014). Chromones as a privileged scaffold in drug discovery: A review. Eur. J. Med. Chem..

[23] Ali Y.M., Seong S.H., Reddy M.R., Seo S.Y., Choi J.S., Jung H.A. (2017). Kinetics and molecular docking studies of 6-formyl umbelliferone isolated from *Angelica decursiva* as an inhibitor of cholinesterase and BACE1. Molecules.

[24] Mphahlele M.J., Olomola T.O. (2019). Synthesis and transformation of 5-acetyl-2-aryl-6-hydroxybenzofurans into furanoflavanone derivatives. Synthesis.

[B25-ijms-20-05451] 25.CCDC-1954844 contains the supplementary crystallographic data of **2i**. These data can be obtained free of charge from The Cambridge Crystallographic Data Centre via www.ccdc.cam.ac.uk/data_request/cif (Accessed 20/09/2019).

[26] Raju B.C., Rao R.N., Suman P., Yogeeswari P., Sriram D., Shaik T.B., Kalivendi S.V. (2011). Synthesis, structure–activity relationship of novel substituted 4*H*-chromen-1,2,3,4-tetrahydropyrimidine-5-carboxylates as potential anti-mycobacterial and anticancer agents. Bioorg. Med. Chem. Lett..

[27] Rollas S., Küçükgüzel Ş.G. (2007). Biological activities of hydrazone derivatives. Molecules.

[28] Pardridge W.M. (2005). The blood-brain barrier: Bottleneck in brain drug development. NeuroRx.

[29] Leroux F.R., Manteau1 B., Vors J.-P., Pazenok S. (2008). Trifluoromethyl ethers – synthesis and properties of an unusual substituent. Beilstein J. Org. Chem..

[30] Hopkins A.L., Groom C.R. (2002). The druggable genome. Nat. Rev. Drug Discov..

[31] Cheewakriengkrai L., Gauthier S. (2013). A 10-year perspective on donepezil. Expet. Opin. Pharmacother..

[32] Marco-Contelles P., do Carmo Carreiras M., Rodríguez C., Villarroya M., García A.G. (2006). Synthesis and pharmacology of galantamine. Chem. Rev..

[33] Gupta S., Mohan C.G. (2014). Dual Binding Site and Selective Acetylcholinesterase Inhibitors Derived from Integrated Pharmacophore Models and Sequential Virtual Screening. Biomed. Res. Int..

[34] Inestrosa N.C., Alvarez A., Perez C.A., Moreno R.D., Vicente M., Linker C., Casanueva O.I., Soto C., Garrido J. (1996). Acetylcholinesterase accelerates assembly of amyloid-β-peptides into Alzheimer’s fibrils: Possible role of the peripheral site of the enzyme. Neuron.

[35] Ballard C.G. (2002). Advances in the treatment of Alzheimer’s disease: Benefits of dual cholinesterase inhibition. Eur. Neurol..

[36] Kang J.E., Cho J.K., Curtis-Long M.J., Ryu H.W., Kim J.H., Kim H.J., Yuk H.K., Kim D.W., Park K.H. (2013). Inhibitory evaluation of sulfonamide chalcones on β-secretase and acylcholinesterase. Molecules.

[37] Di Meco A., Lauretti E., Vagnozzi A.N., Praticò D. (2014). Zileuton restores memory impairments and reverses amyloid and tau pathology in aged Alzheimer’s disease mice. Neurobiol. Aging.

[38] Czapski G.A., Czubowicz K.J., Strosznajder B., Strosznajder R.P. (2016). The lipoxygenases: Their regulation and implication in Alzheimer’s disease. Neurochem. Res..

[39] Kim H.P., Mani I., Iversen L., Ziboh V.A. (1998). Effects of naturally-occurring flavonoids and bioflavonoids on epidermal cyclooxygenase and lipoxygenase from guinea-pigs. Prostaglandins Leukot. Essent. Fat. Acids.

[40] Pergola C., Werz O. (2010). 5-Lipoxygenase inhibitors: A review of recent developments and patents. Expert Opin. Ther. Pat..

[41] Ortiz G.G., Pacheco Moisés F.P., Mireles-Ramírez M., Flores-Alvarado L.J., González-Usigli H., Sánchez-González V.J., Sánchez-López A.L., Sánchez-Romero L., Díaz-Barba E.I., Santoscoy-Gutiérrez F. (2017). Oxidative stress: Love and hate history in central nervous system. Adv. Protein Chem. Struct. Biol..

[42] Bonda D.J., Wang X.L., Perry G., Nunomura A., Tabaton M., Zhu X.W., Smith M.A. (2010). Oxidative stress in Alzheimer disease: A possibility for prevention. Neuropharmacology.

[43] Ellman G.L., Courtney K.D., Andres V., Featherstone R.M. (1961). A new and rapid colorimetric determination of acetylcholinesterase activity. Biochem. Pharmacol..

[44] Aminudin N.I., Ahmad F., Taher M., Zulkifli R.M. (2015). Glucosidase and 15-lipoxygenase inhibitory activities of phytochemicals from Calophyllum symingtonianum. Nat. Prod. Commun..

[45] Mphahlele M.J., Agbo E.N., Gildenhuys S. (2018). Synthesis and evaluation of the 4-substituted 2-hydroxy-5-iodochalcones and their 7-substituted 6-iodoflavonol derivatives for inhibitory effect on cholinesterases and β-secretase. Int. J. Mol. Sci..

[46] Zhu K., Zhou H., Qian H. (2006). Antioxidant and free radical scavenging activities of wheat germ protein hydrolysates (WGPH) prepared with alcalase. Process. Biochem..

