# Supplementary material for: In Vitro Evaluation and Docking Studies of 5-oxo-5H-furo[3,2-g]chromene-6-carbaldehyde Derivatives as Potential Anti-Alzheimer’s Agents
Source: Int J Mol Sci. 2019 Nov 1;20(21):5451. doi: 10.3390/ijms20215451 (PMC6862662; doi:10.3390/ijms20215451)
Supplement: Supplementary file 1 [file ijms-20-05451-s001.pdf]

Supplementary Materials

Figure S1: Copies of ^{1}H-NMR and ^{13}C-NMR spectra for compounds **1**, **2a–i** and **3a–i**.

Figure S2.1: Lineweaver-Burk and Dixon plots of **2b**, **2f**, **3b**, **3d** and **3f** against AChE.

Figure S2.2: Lineweaver-Burk and Dixon plots of **2b**, **2f**, **3b**, **3d** and **3f** against BChE.

Figure S2:3: Lineweaver-Burk and Dixon plots of **2f** against BACE-1.

Figure S3: Docking pose of donepezil showing interaction with AChE protein residues.

Figure S4: Docking pose of donepezil showing interaction with BChE protein residues.

Figure S5 Docking pose of quercetin showing interaction with BACE-1 protein residues.

Figure S6: Docking pose of zileuton showing interaction with LOX-5 protein residues.

Figure S8: Evaluation of toxicity of **2f** in Hek293-T cells.

Figure S1: Copies of ^{1}H-NMR and ^{13}C-NMR spectra for compounds 1, 2a–i and 3a–i.

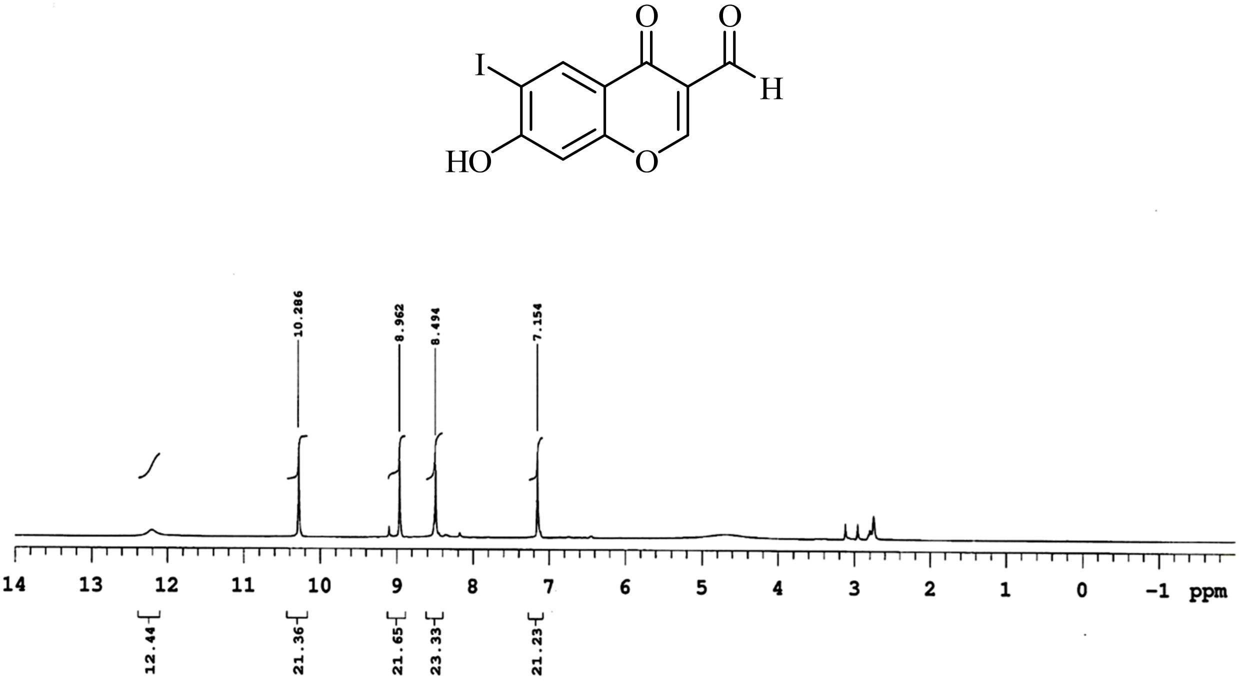

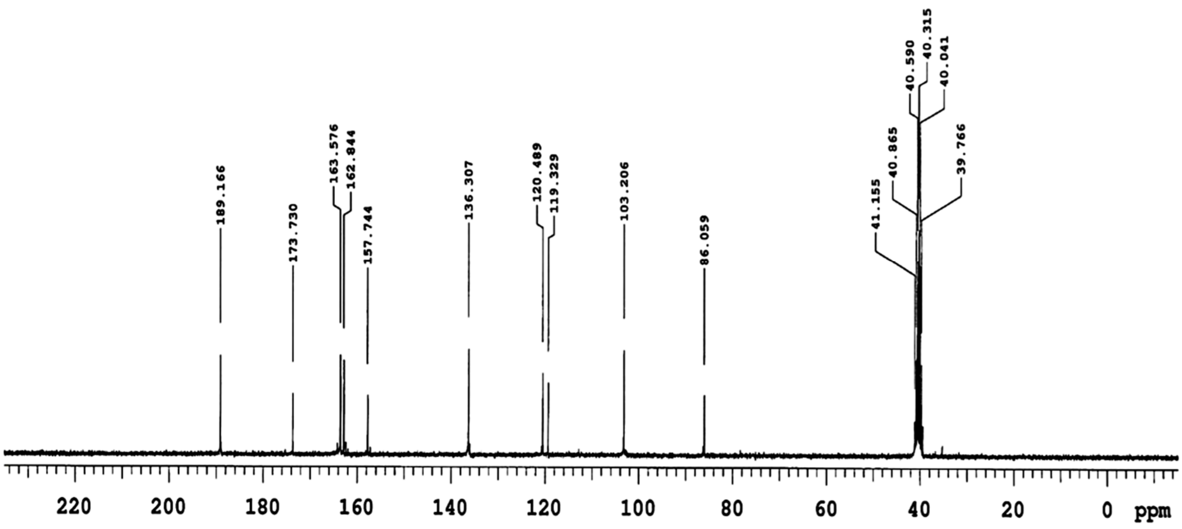

Figure S1.1: ^{1}H- and ^{13}C-NMR spectra of **1** in DMSO-d_6 at 300 and 75 MHz, respectively.

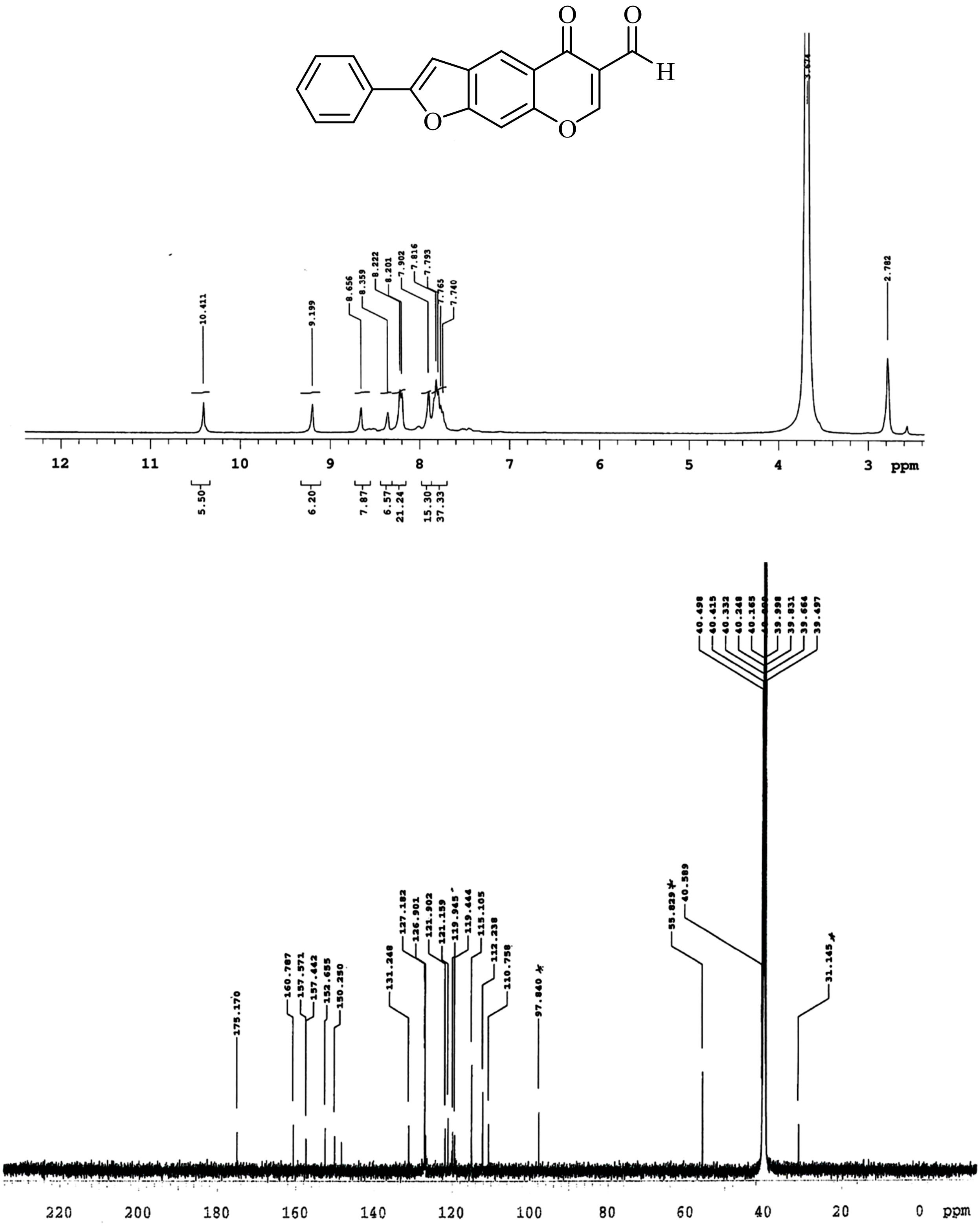

Figure S1.3: ^{1}H- and ^{13}C-NMR spectra of **2a** in DMSO-d_6 at 300 and 75 MHz, respectively.

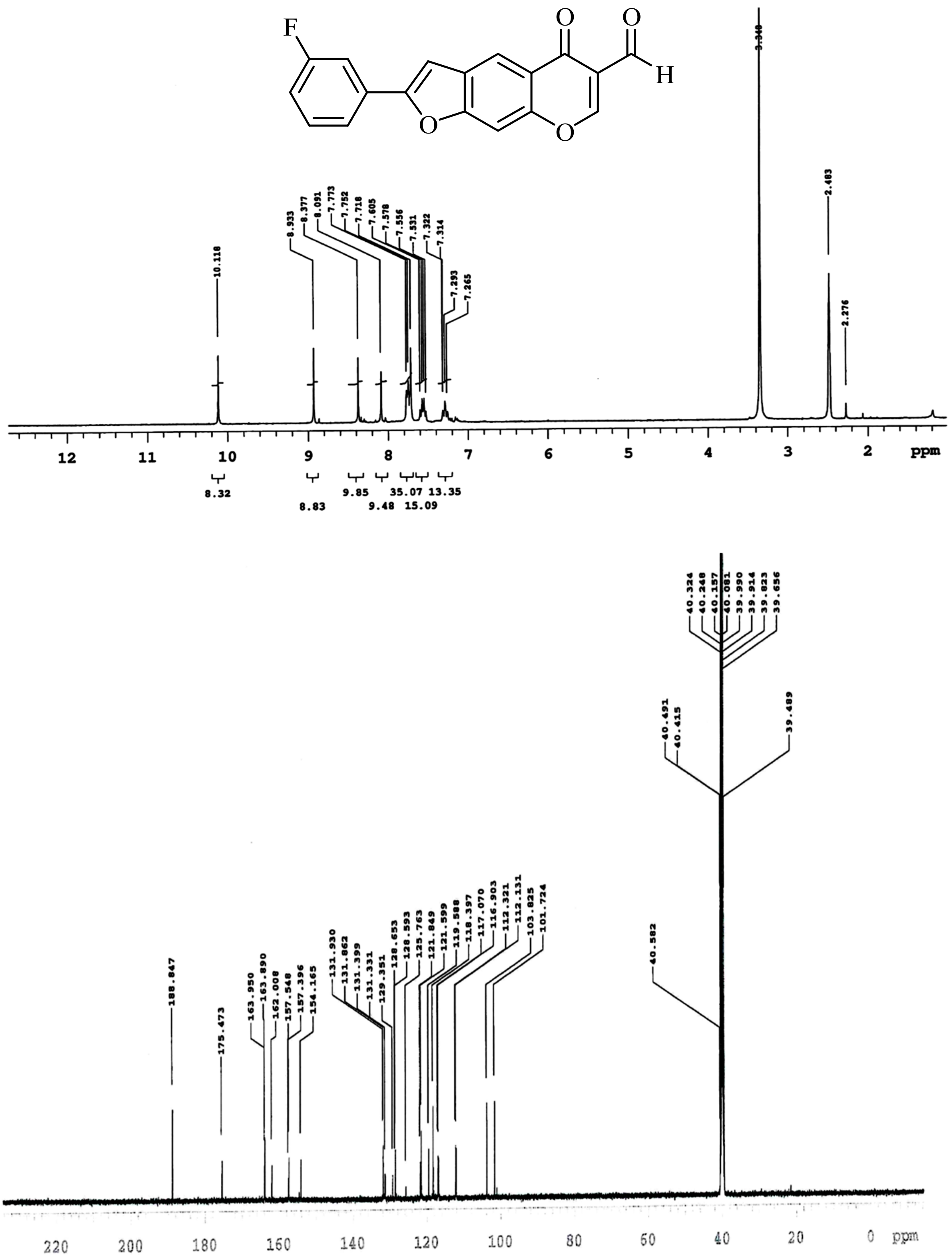

Figure S1.4: ^{1}H- and ^{13}C-NMR spectra of **2b** in DMSO-d_6 at 300 and 75 MHz, respectively.

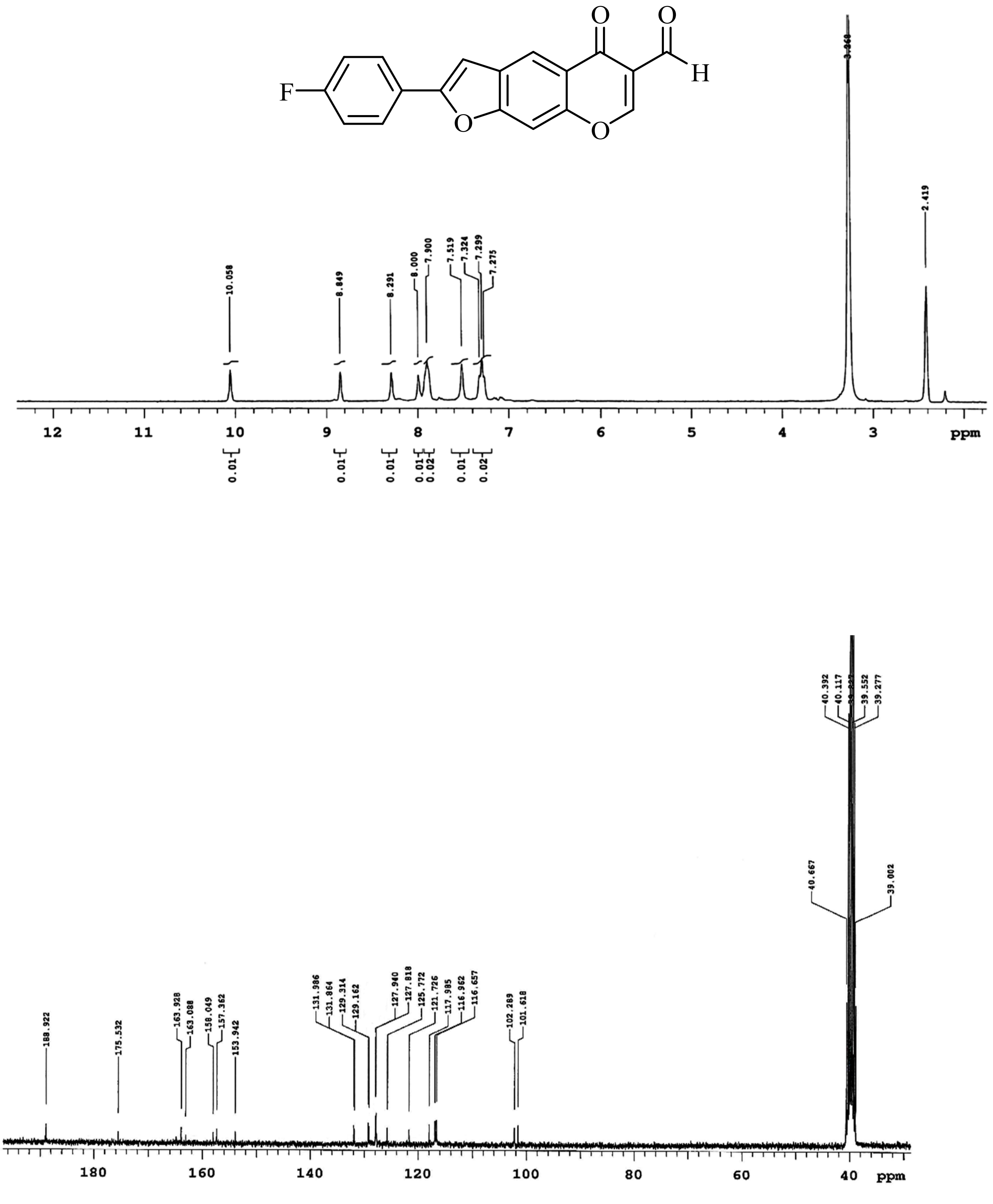

Figure S1.5: ^{1}H- and ^{13}C-NMR spectra of **2c** in DMSO-d_6 at 300 and 75 MHz, respectively.

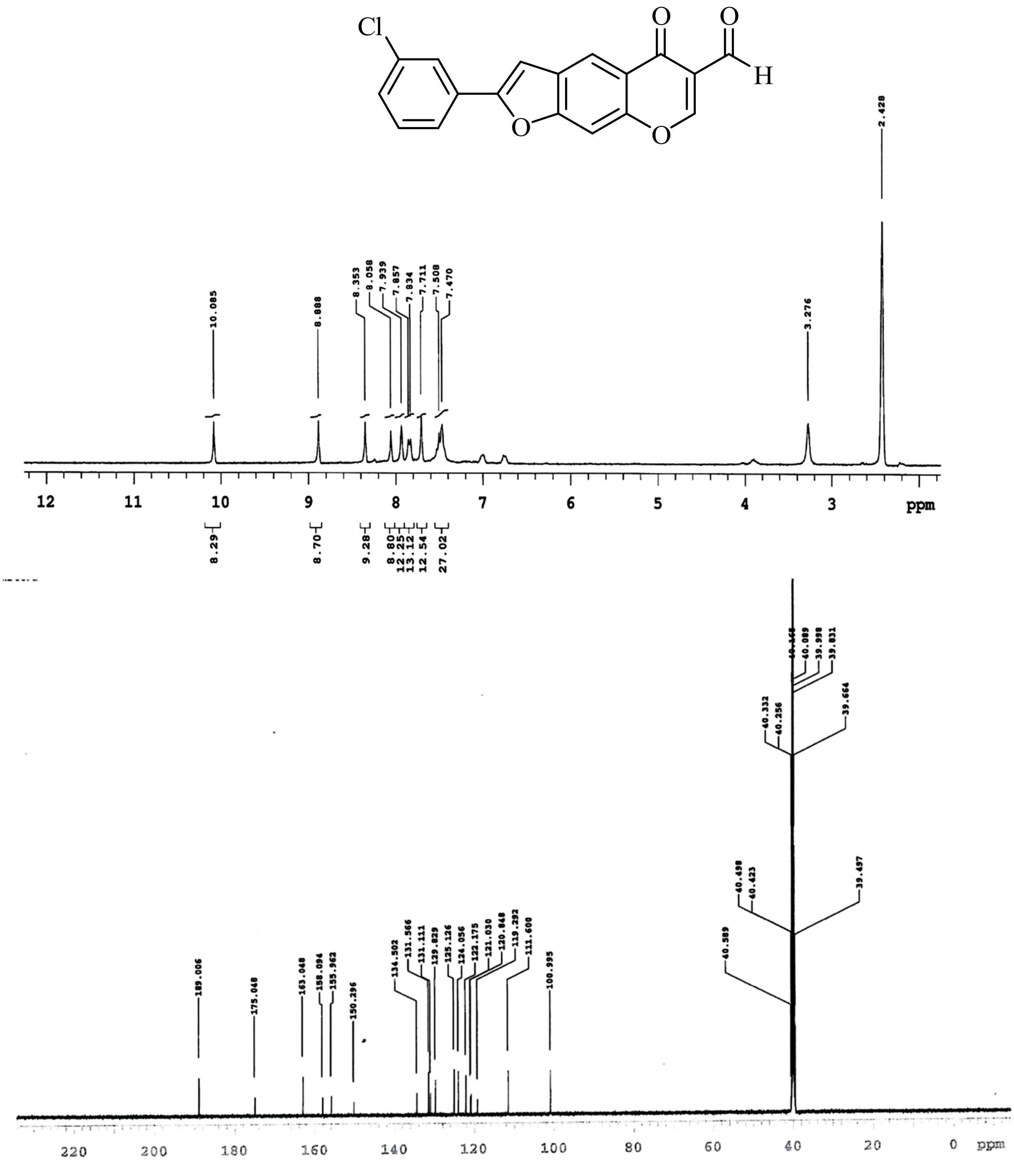

Figure S1.6: ^{1}H- and ^{13}C-NMR spectra of **2d** in DMSO-d_6 at 300 and 75 MHz, respectively.

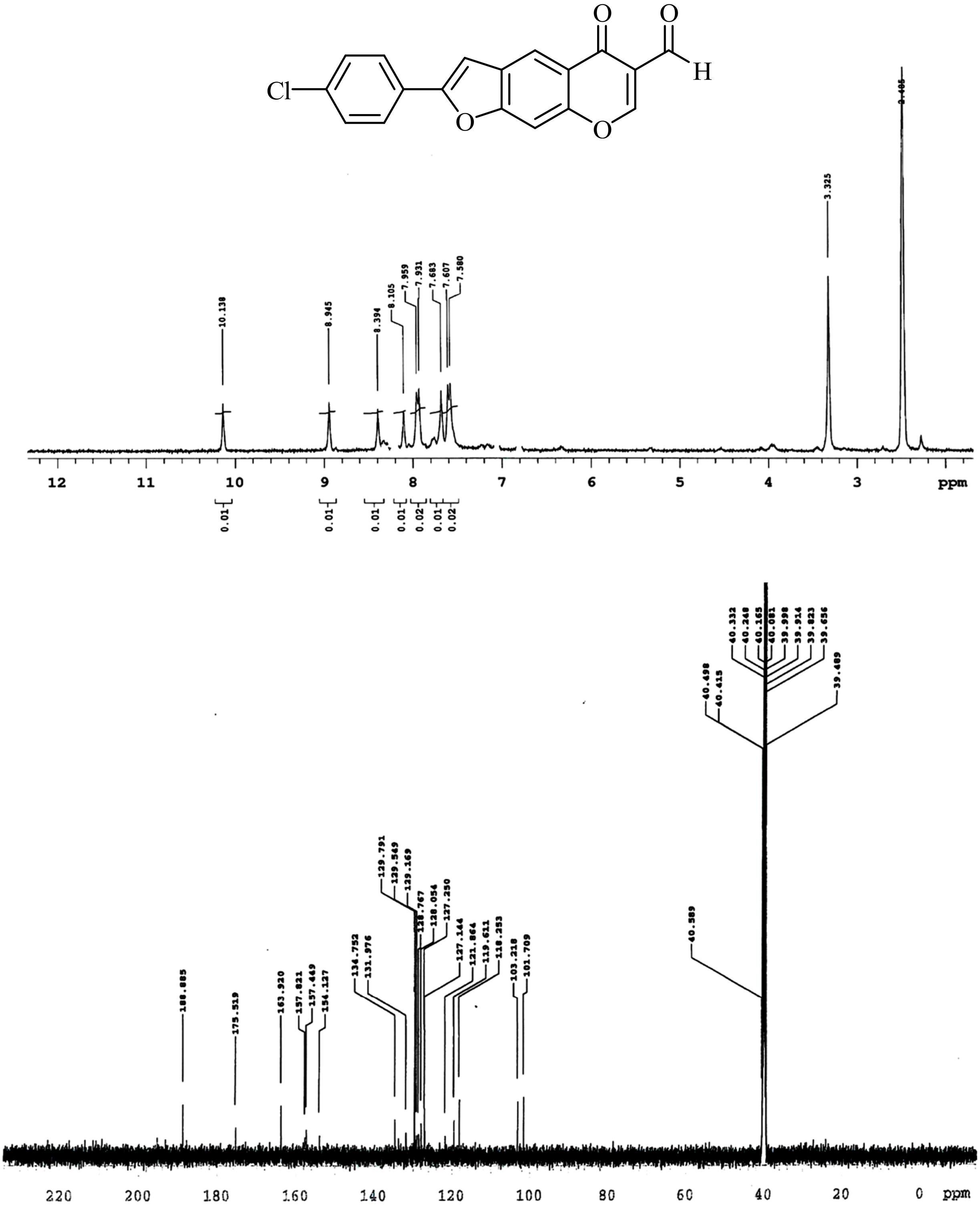

Figure S1.7: ^{1}H- and ^{13}C-NMR spectra of **2e** in DMSO-d_6 at 300 and 75 MHz, respectively.

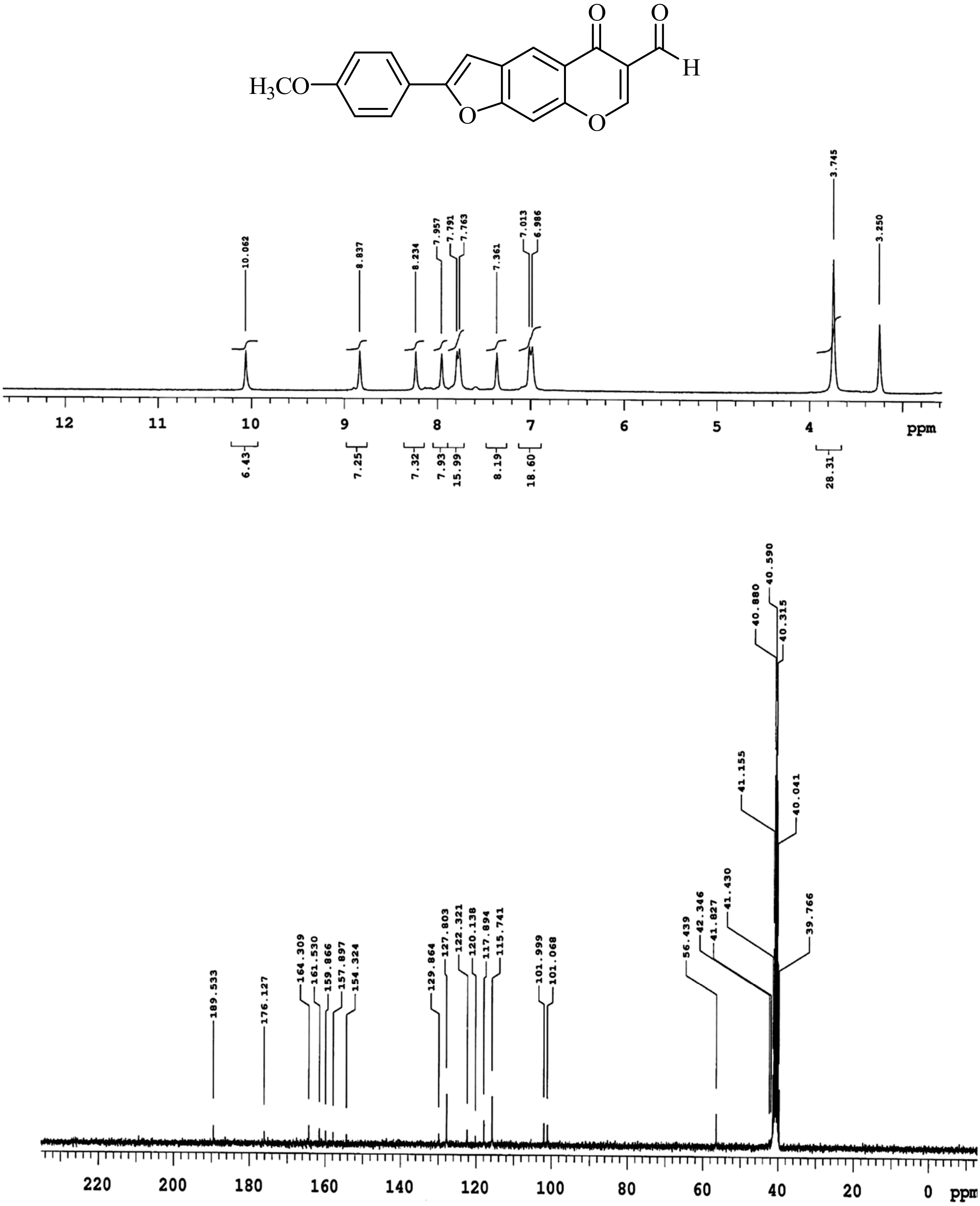

Figure S1.8: ^{1}H- and ^{13}C-NMR spectra of **2f** in DMSO-d_6 at 300 and 75 MHz, respectively.

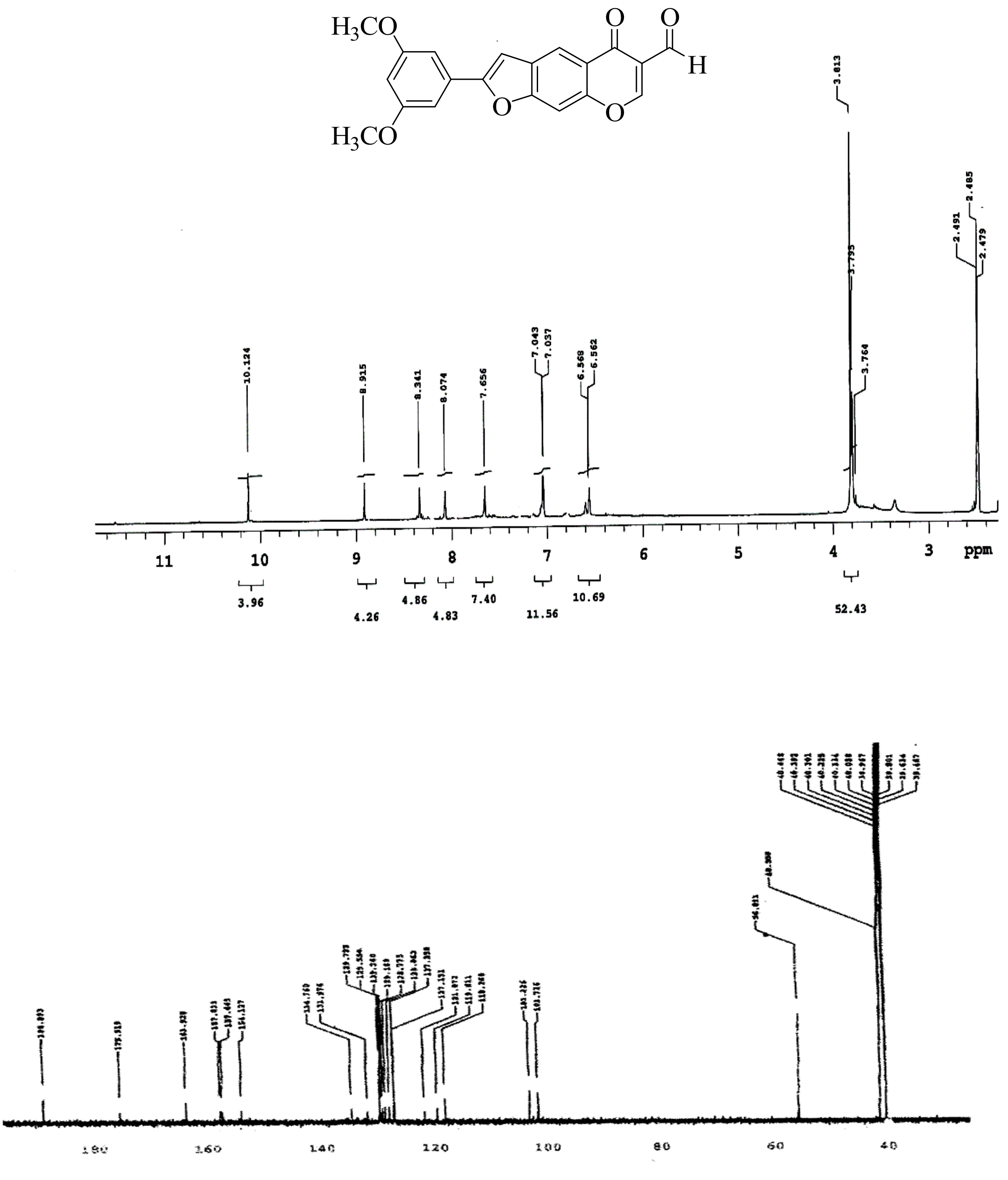

Figure S1.9: ^{1}H- and ^{13}C-NMR spectra of **2g** in DMSO-d_6 at 300 and 75 MHz, respectively

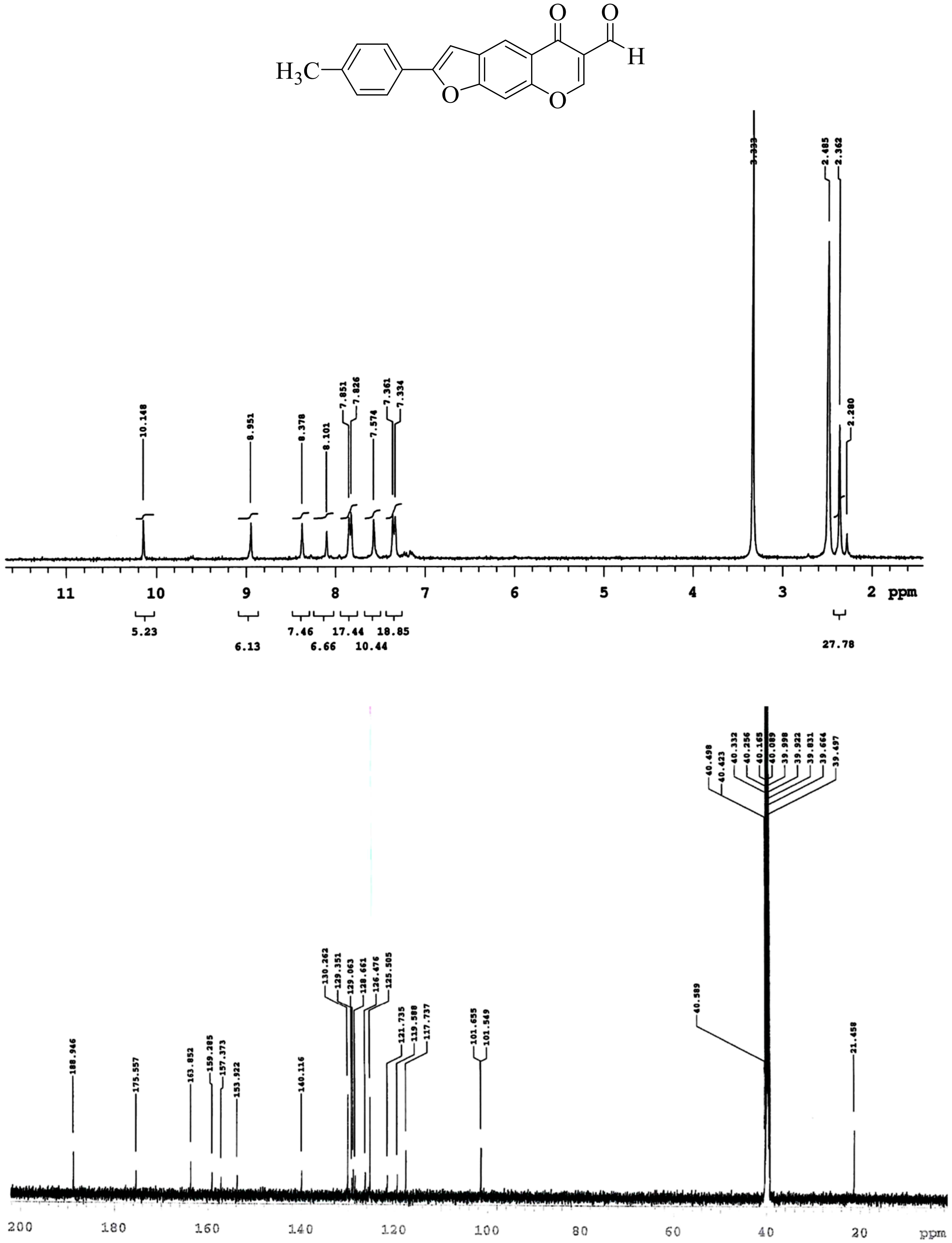

Figure S1.10: ^{1}H- and ^{13}C-NMR spectra of **2h** in DMSO-d_6 at 300 and 75 MHz, respectively.

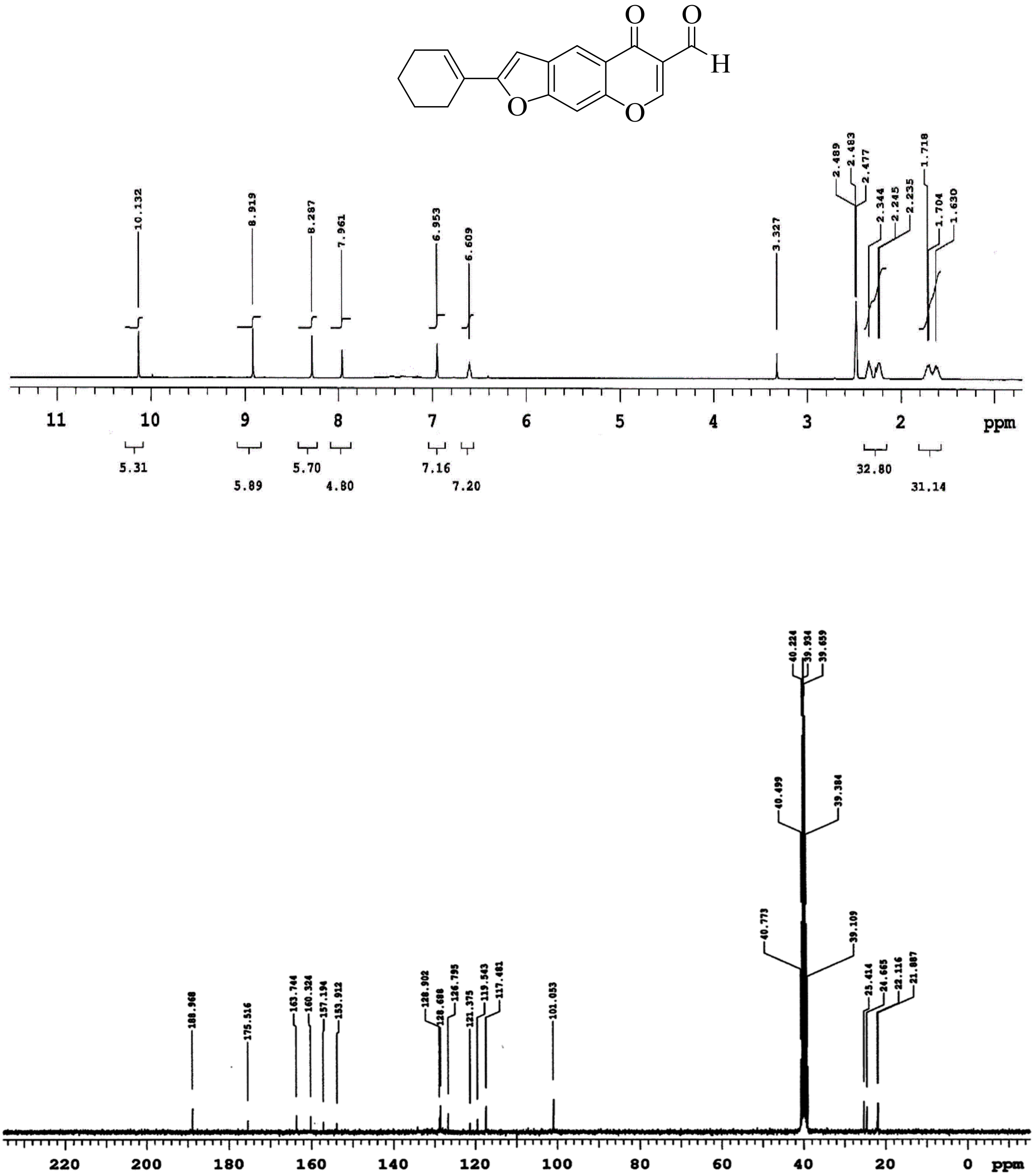

Figure S1.11: ^{1}H- and ^{13}C-NMR spectra of **2i** in DMSO-d_6 at 300 and 75 MHz, respectively.

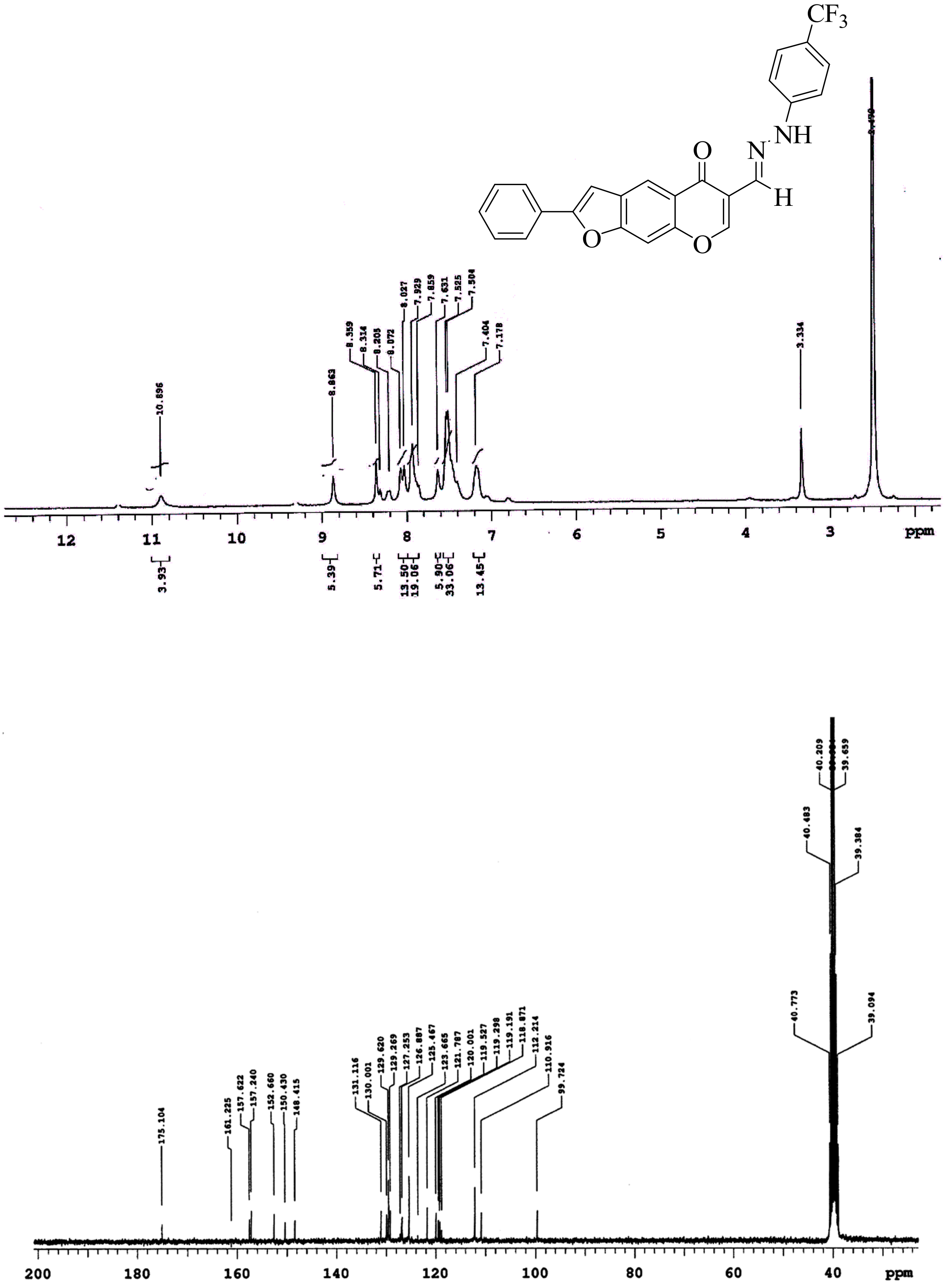

Figure S1.12: ^{1}H- and ^{13}C-NMR spectra of **3a** in DMSO-d_6 at 300 and 75 MHz, respectively.

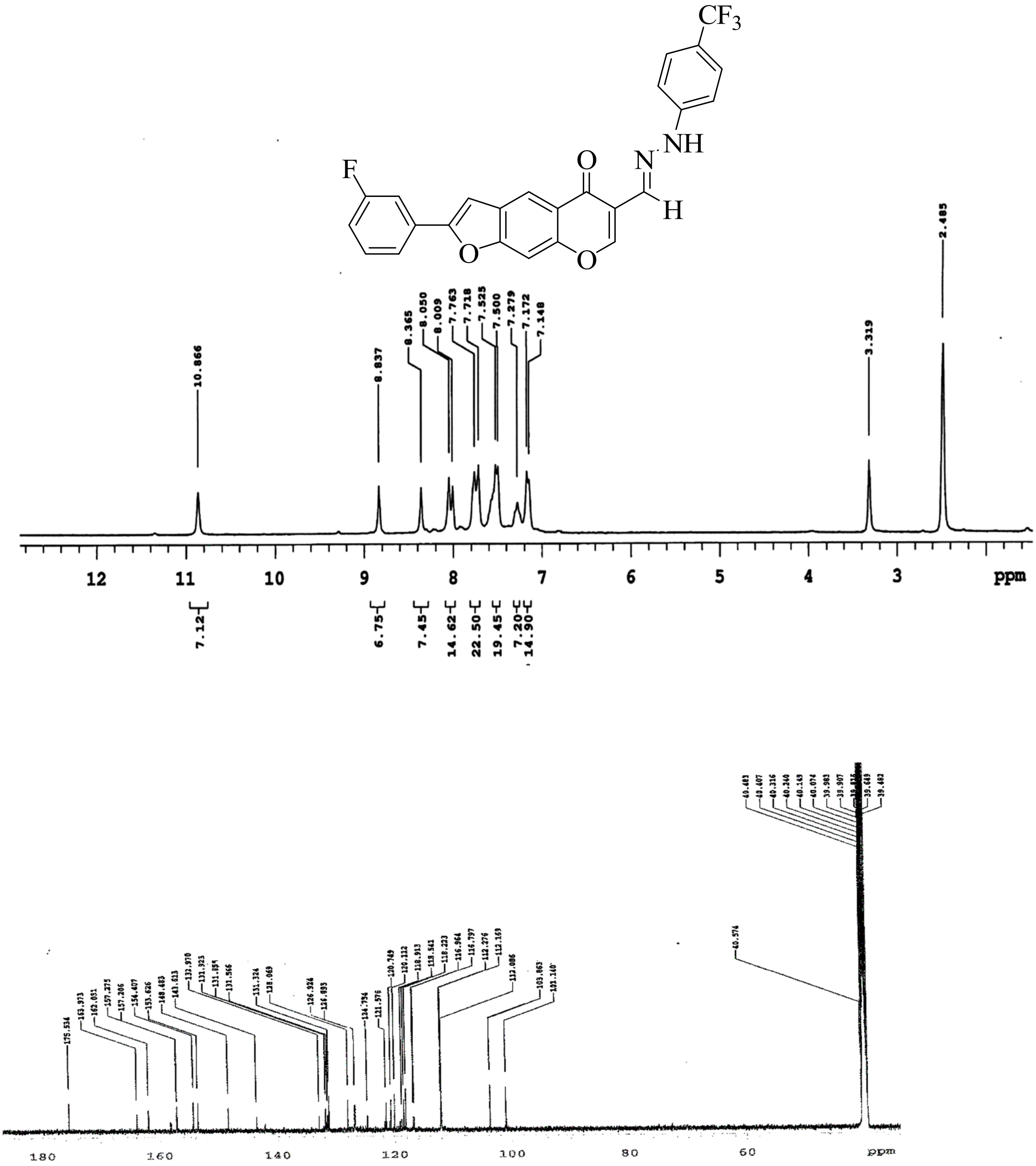

Figure S1.13: ^{1}H- and ^{13}C-NMR spectra of **3b** in DMSO-d_6 at 300 and 75 MHz, respectively.

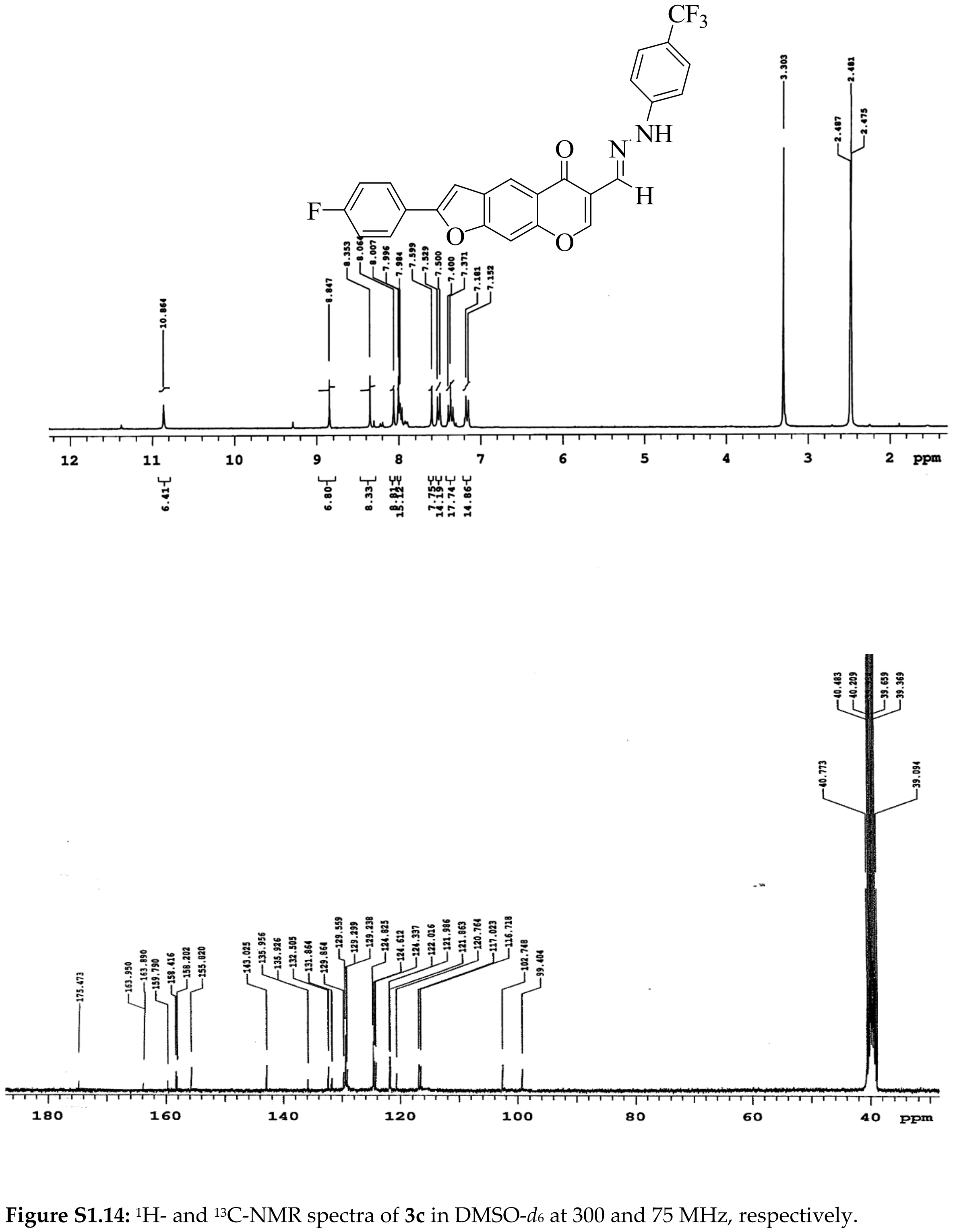

Figure S1.14: ^{1}H- and ^{13}C-NMR spectra of **3c** in DMSO-d_6 at 300 and 75 MHz, respectively.

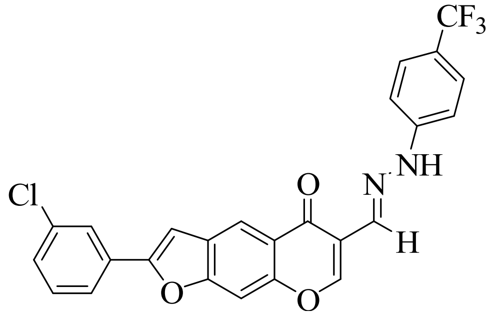

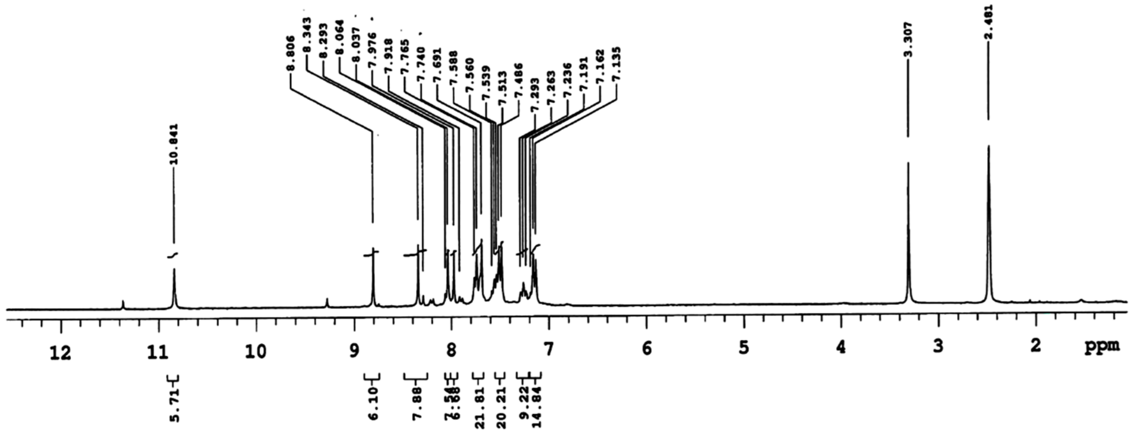

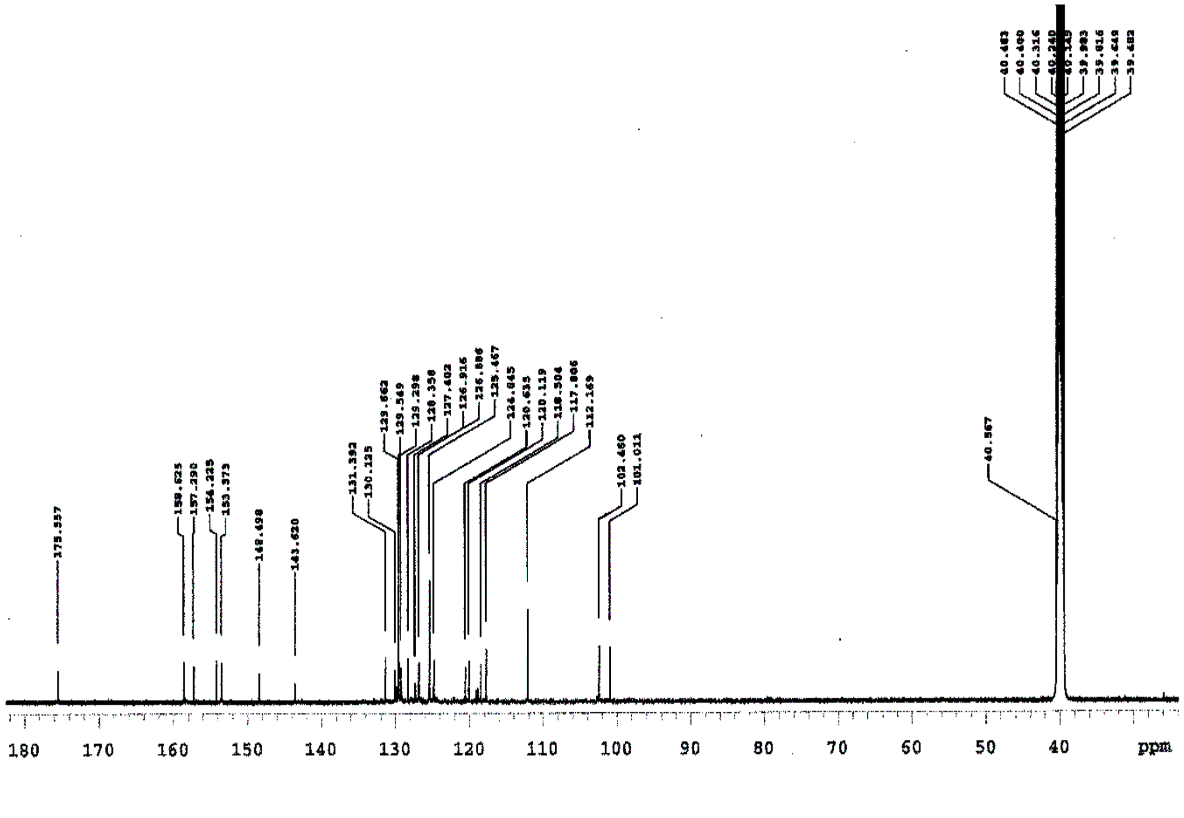

Figure S1.15: ^{1}H- and ^{13}C-NMR spectra of **3d** in DMSO-d_6 at 300 and 75 MHz, respectively.

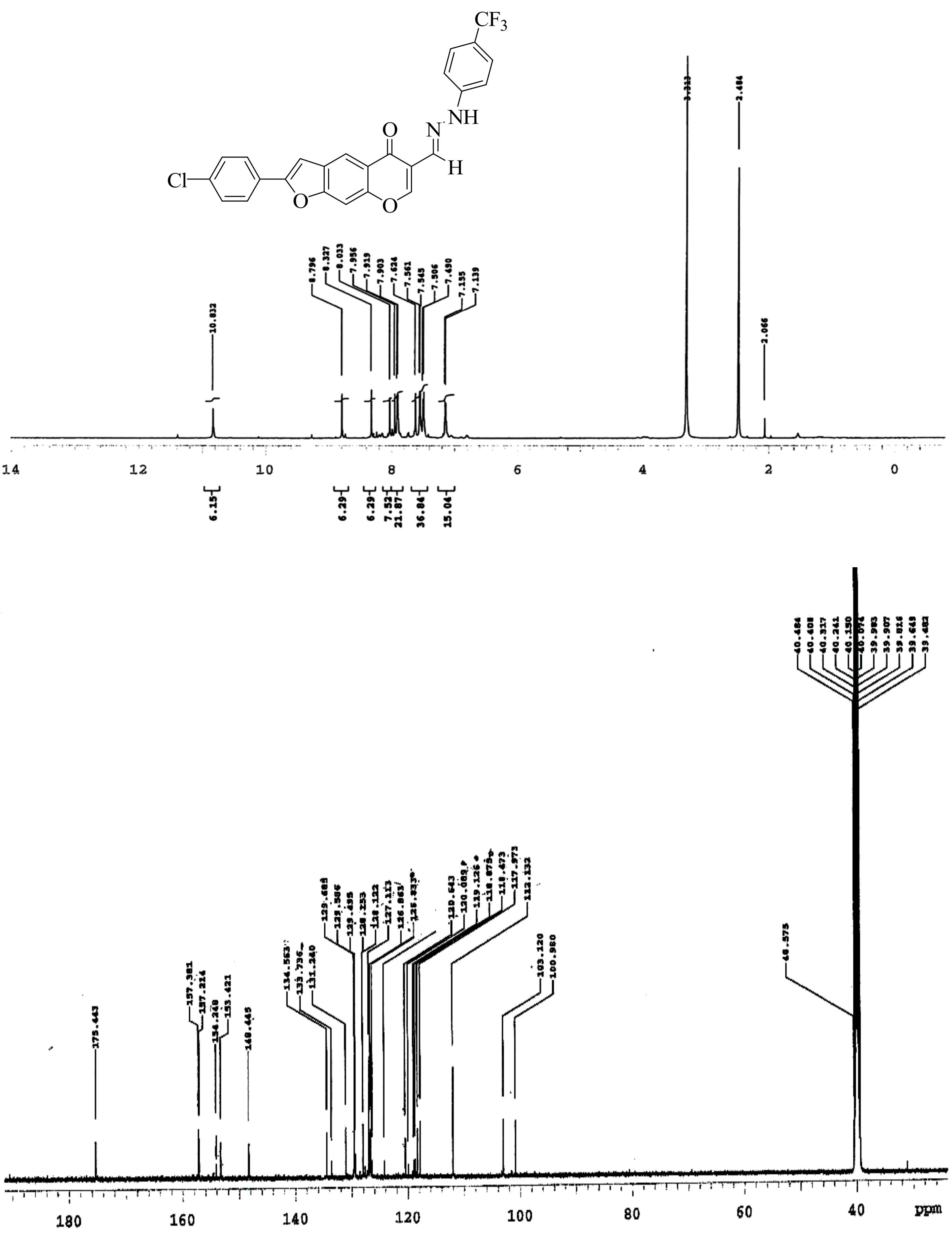

Figure S1.16: ^{1}H- and ^{13}C-NMR spectra of **3e** in DMSO-d_6 at 300 and 75 MHz, respectively.

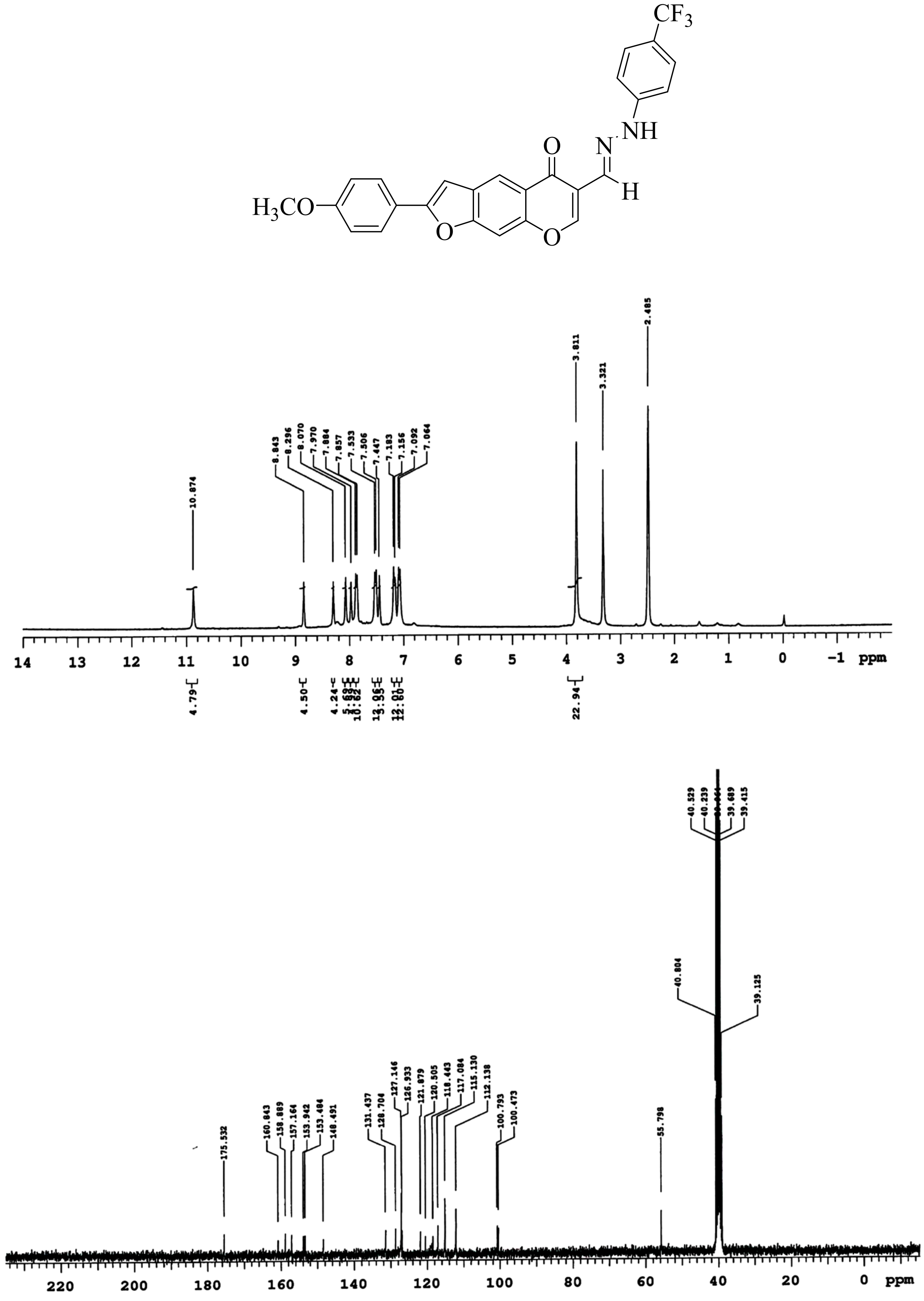

Figure S1.17: ^{1}H- and ^{13}C-NMR spectra of **3f** in DMSO-d_6 at 300 and 75 MHz, respectively.

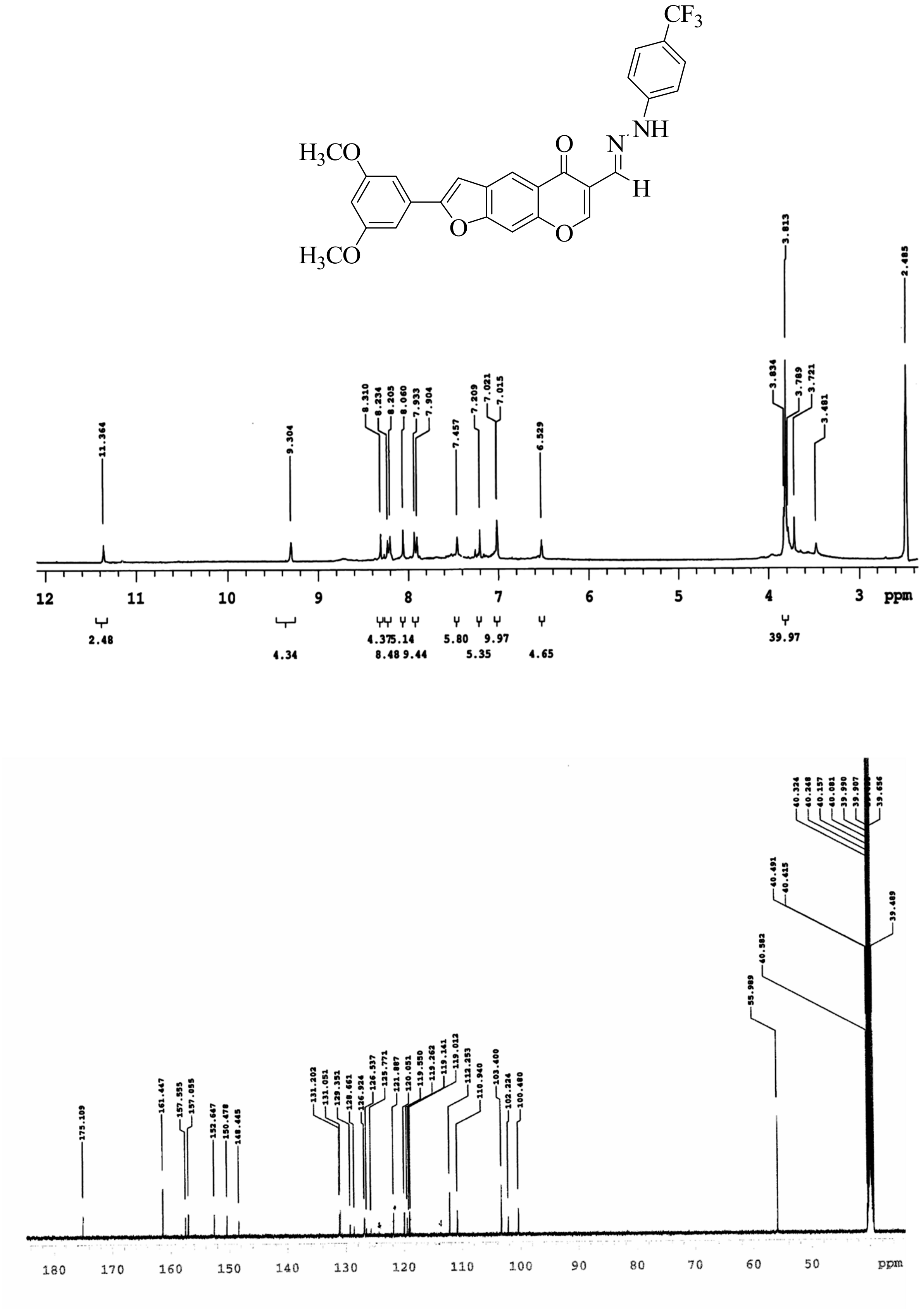

Figure S1.18: ^{1}H- and ^{13}C-NMR spectra of **3g** in DMSO-d_6 at 300 and 75 MHz, respectively.

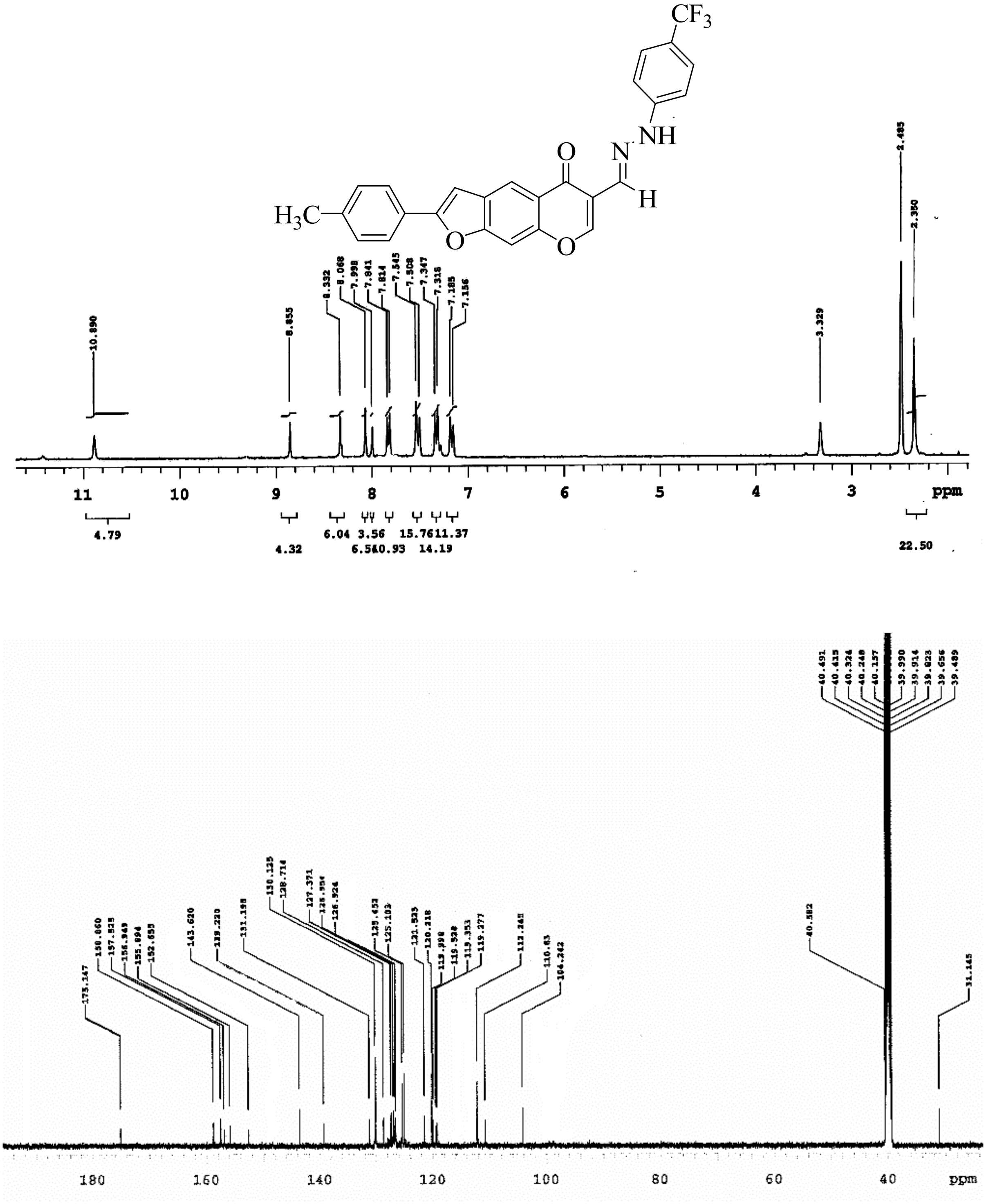

Figure S1.19: ^{1}H- and ^{13}C-NMR spectra of **3h** in DMSO-*d*$_6$ at 300 and 75 MHz, respectively.

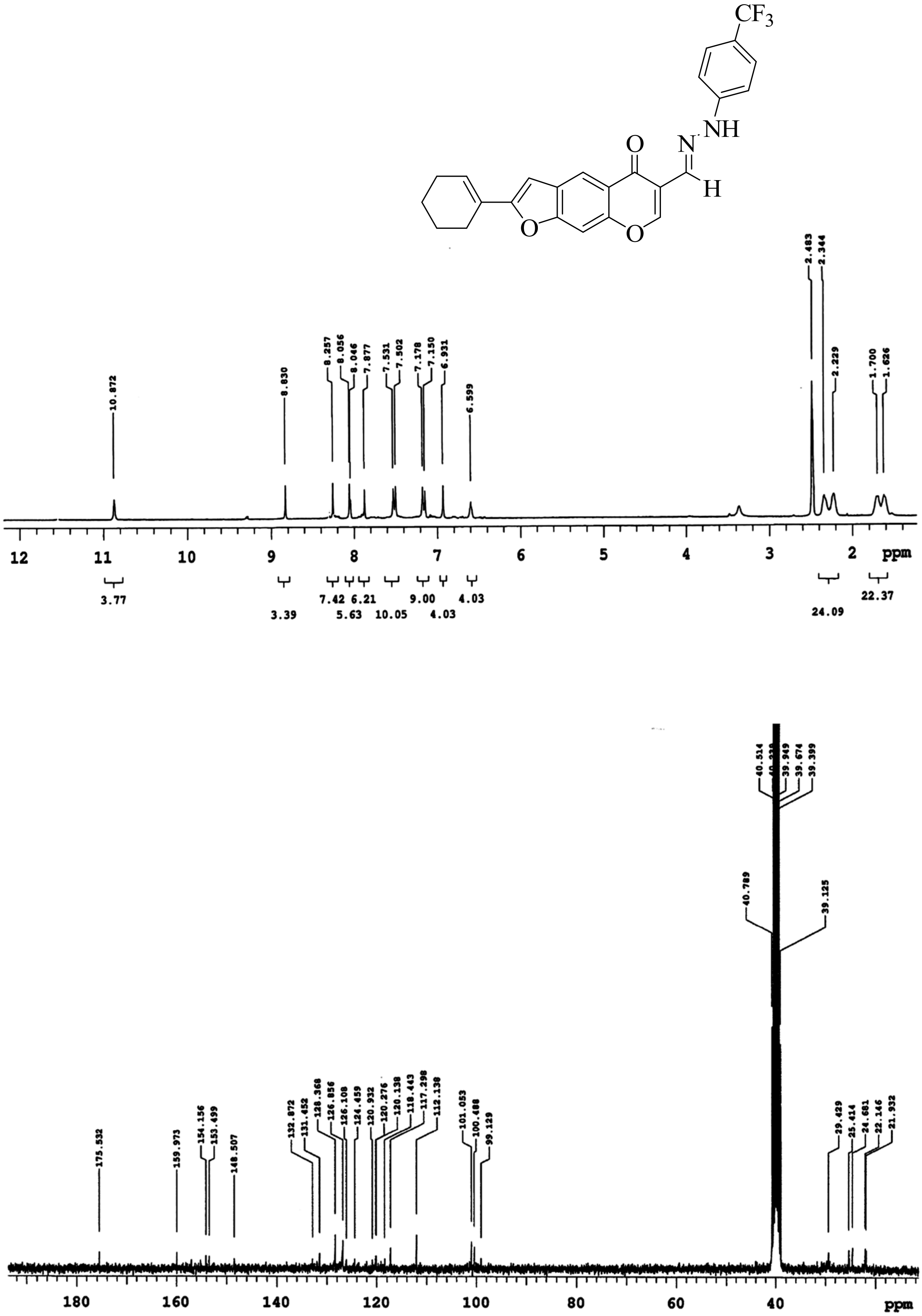

Figure S1.20: ^{1}H- and ^{13}C-NMR spectra of **3i** in DMSO-d_6 at 300 and 75 MHz, respectively.

Figure S2.1: Lineweaver-Burk and Dixon plots of 2b, 2f, 3b, 3d and 3f against ACh.

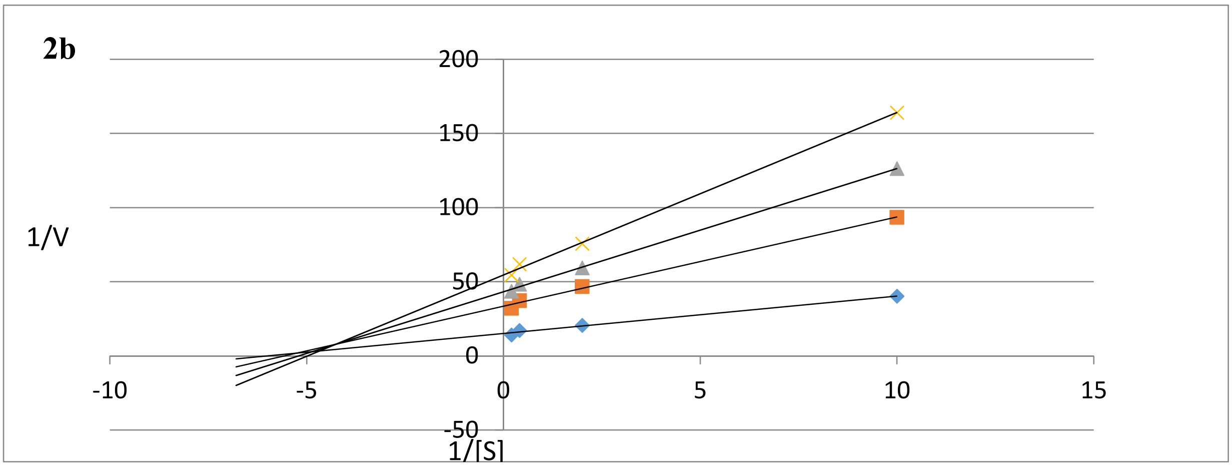

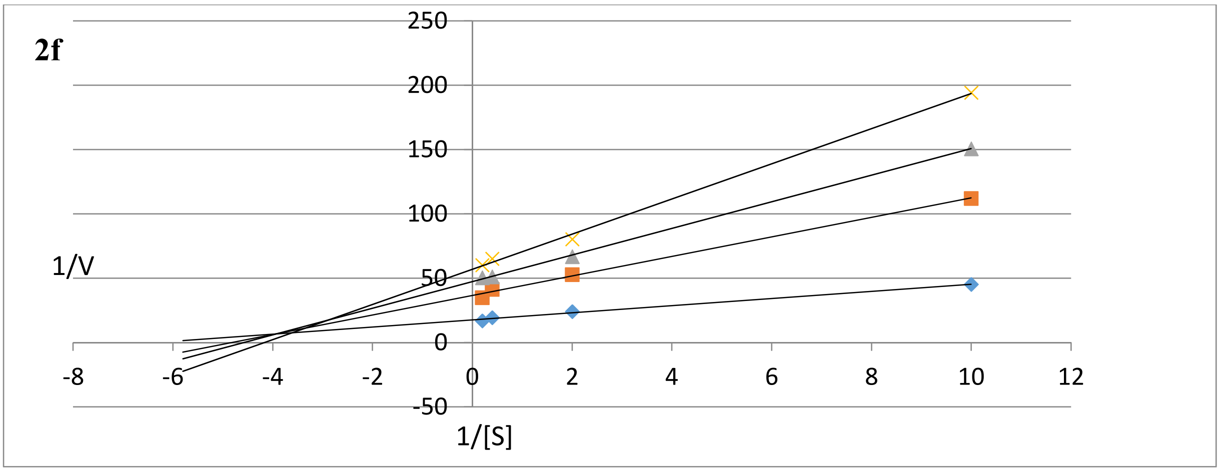

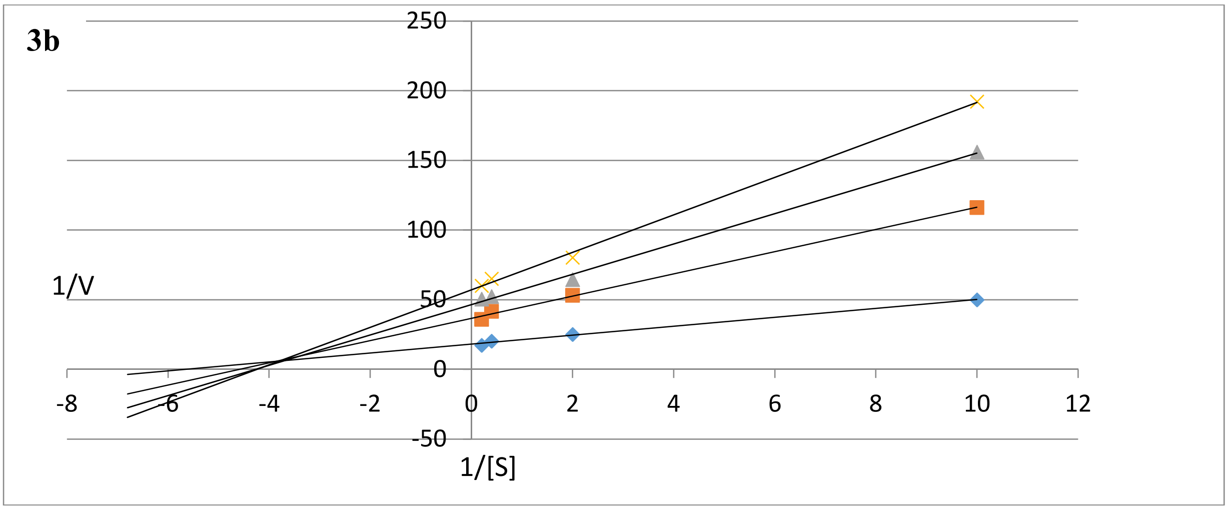

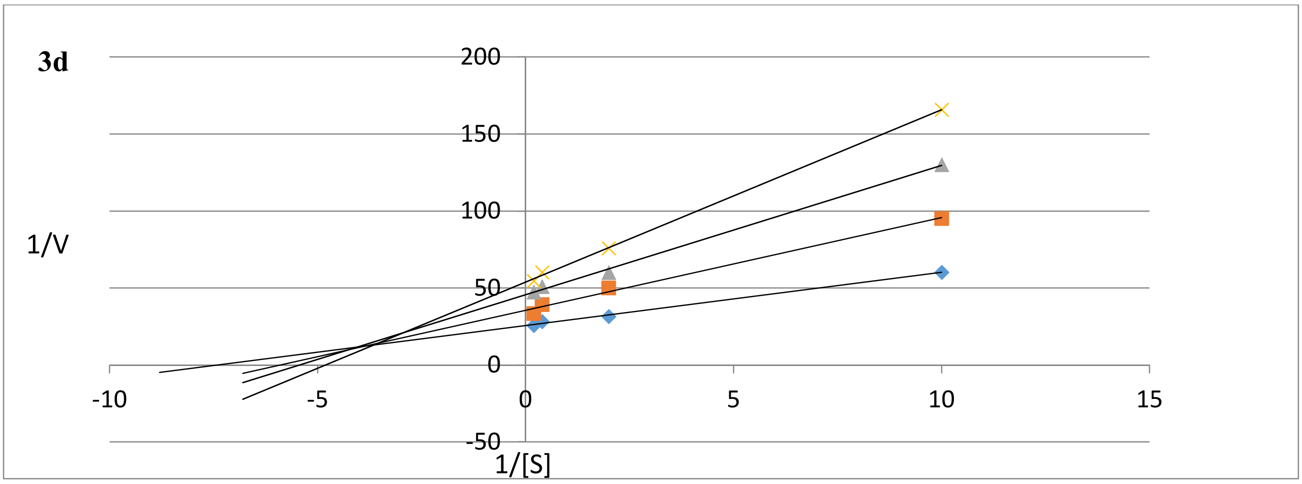

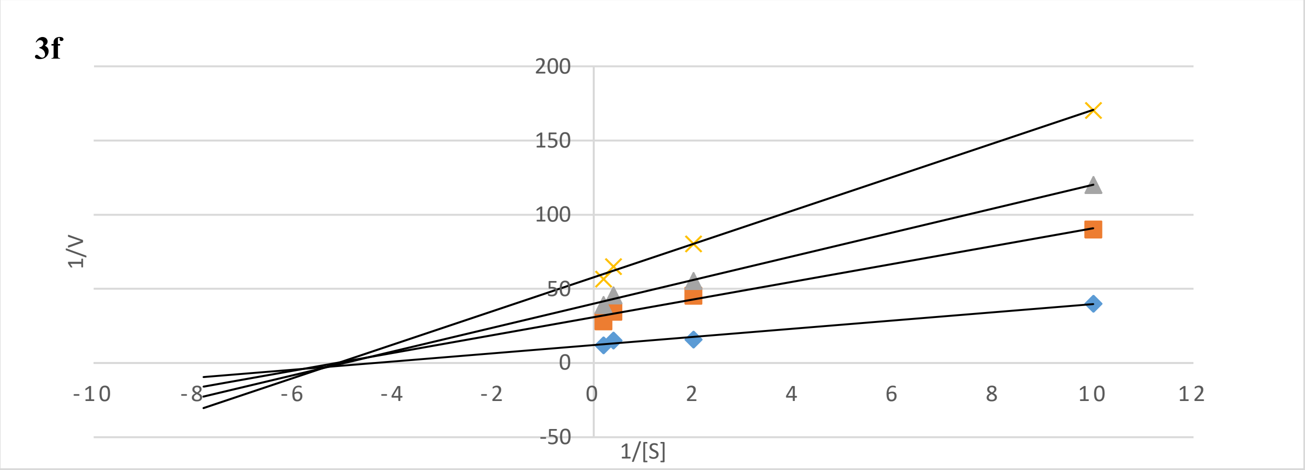

Figure S2.1.1: Lineweaver–Burk plots for inhibition of AChE by **2b**, **2f**, **3b**, **3d**, **3f**. Blue symbols and fitted straight lines represent enzyme activity in the absence of inhibitor, while orange (2.5 μM), grey (3.5 μM) and yellow (5 μM) represent various concentrations of inhibitor.

2b

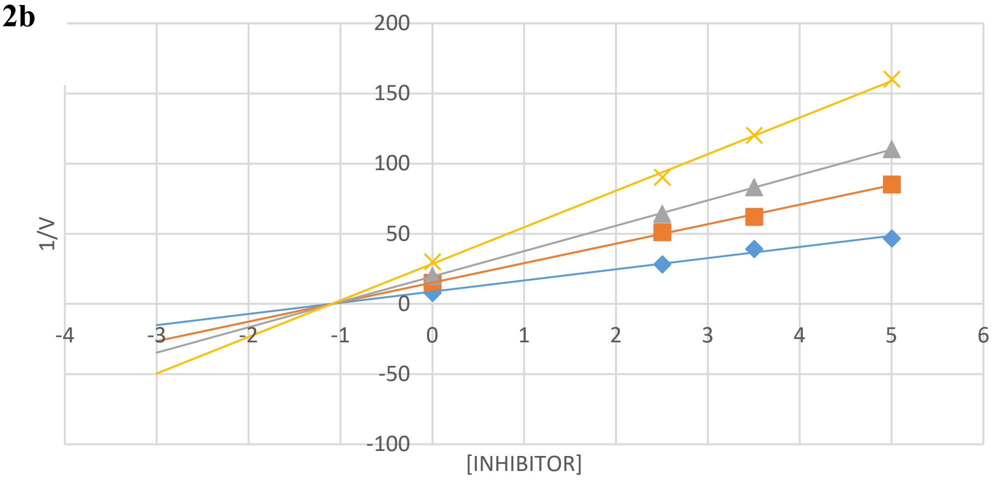

200
150
100
50
0
-50
-100
1/V
-4
-3
-2
-1
0
1
2
3
4
5
6
[INHIBITOR]

2f

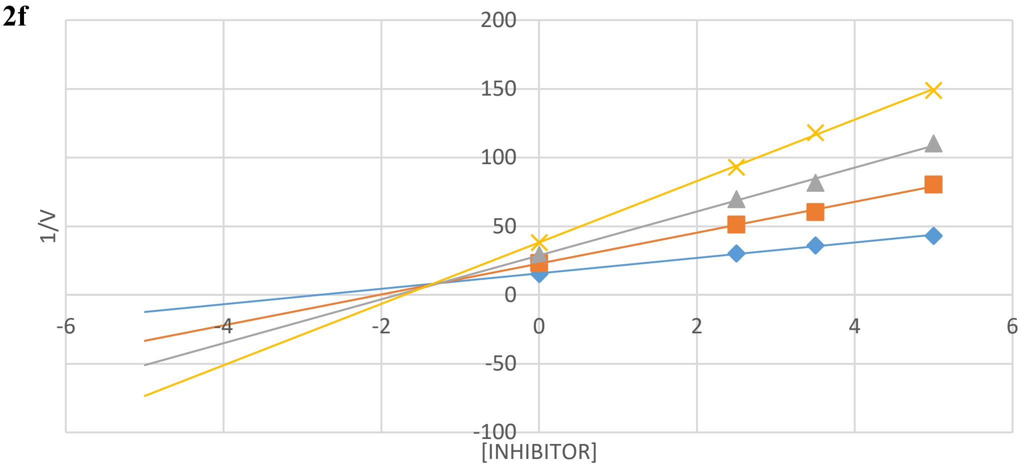

200
150
100
50
0
-50
-100
1/V
-6
-4
-2
0
2
4
6
[INHIBITOR]

3b

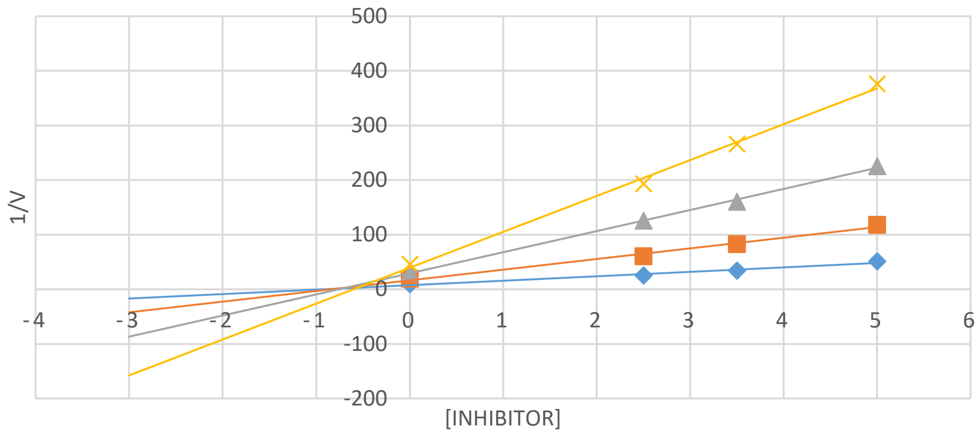

500
400
300
200
100
0
-100
-200
1/V
-4
-3
-2
-1
0
1
2
3
4
5
6
[INHIBITOR]

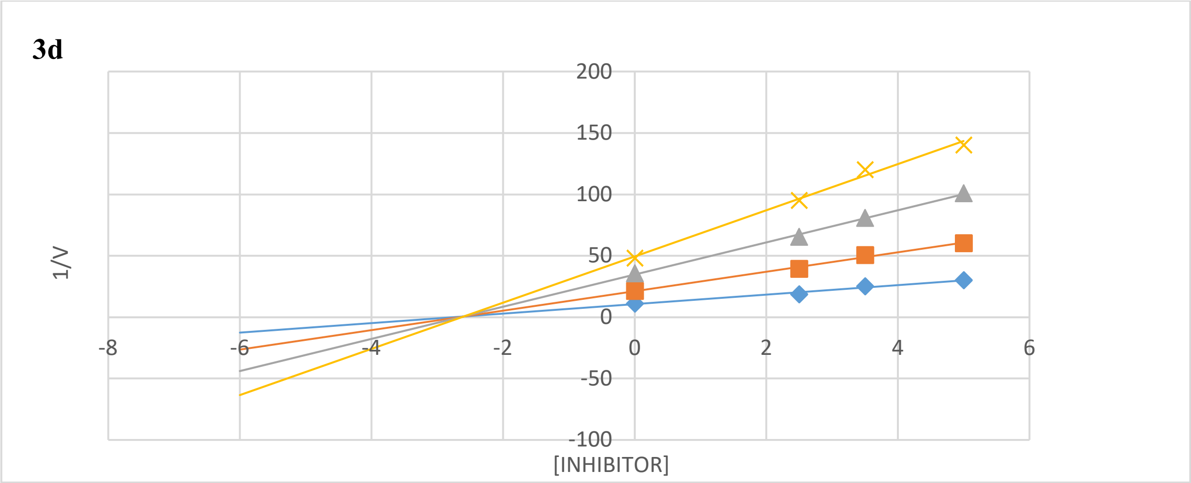

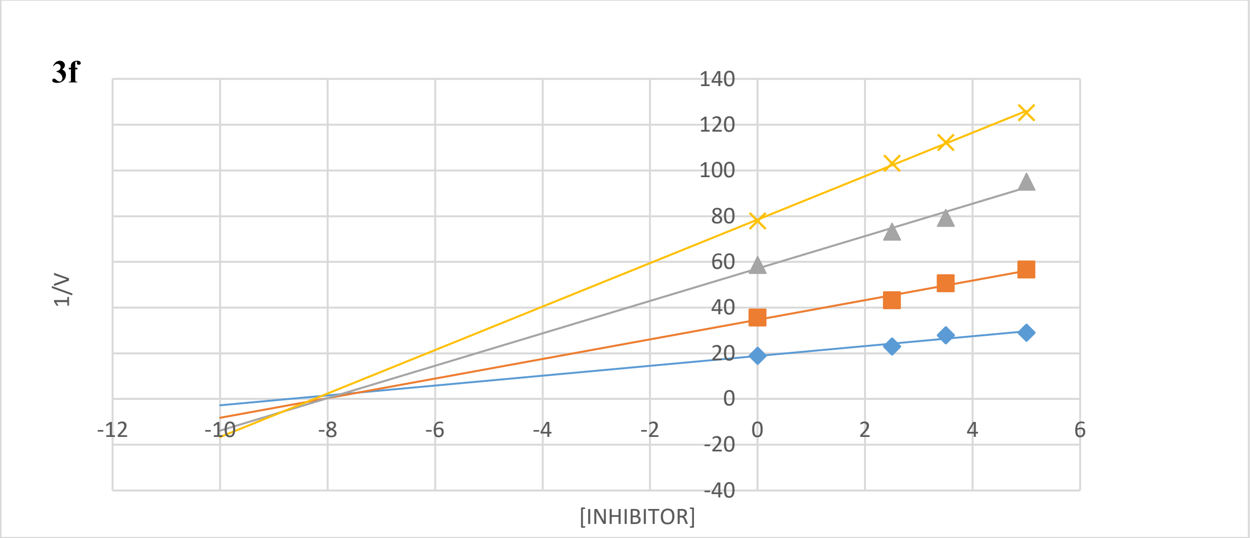

Figure S2.1.2: Dixon plots for inhibition of AChE by **2b**, **2f**, **3b**, **3d**, **3f**. Blue symbols and fitted straight lines represent enzyme activity with 5 mM substrate, while orange (2.5 mM), grey (0.5 mM) and yellow (0.1 mM) represent various concentrations of substrate.

Figure S2.2: **Lineweaver-Burk and Dixon plots of 2b, 2f, 3b, 3d and 3f against BChE.**

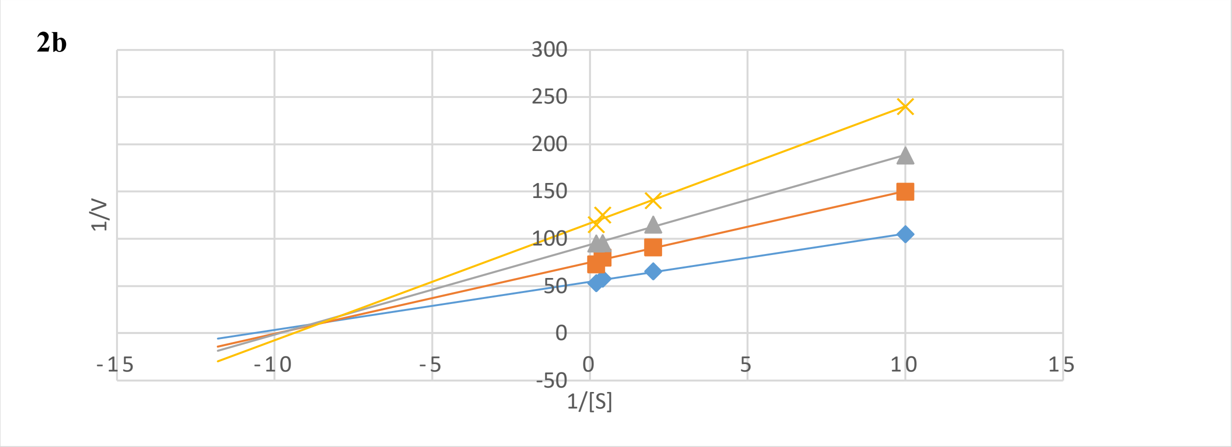

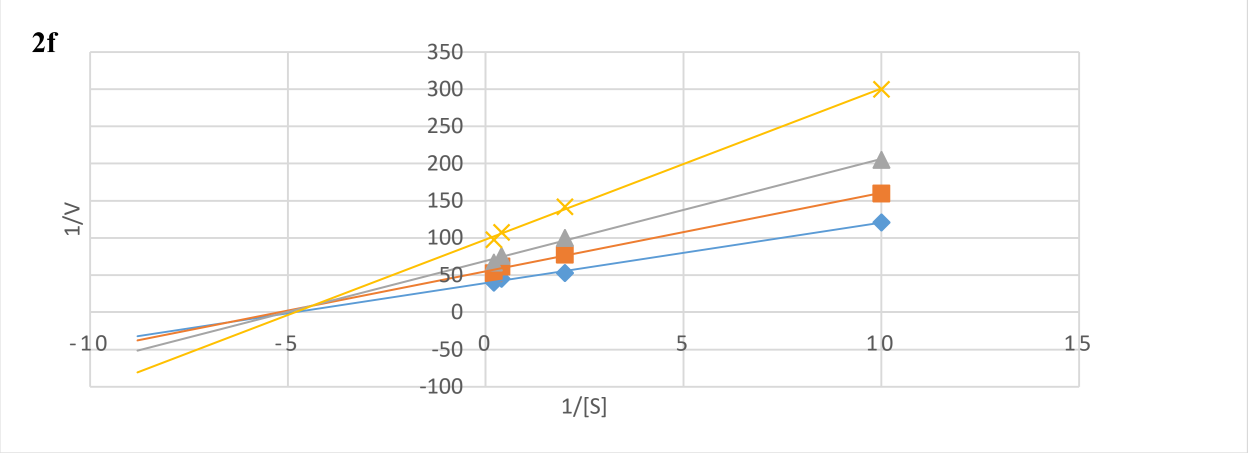

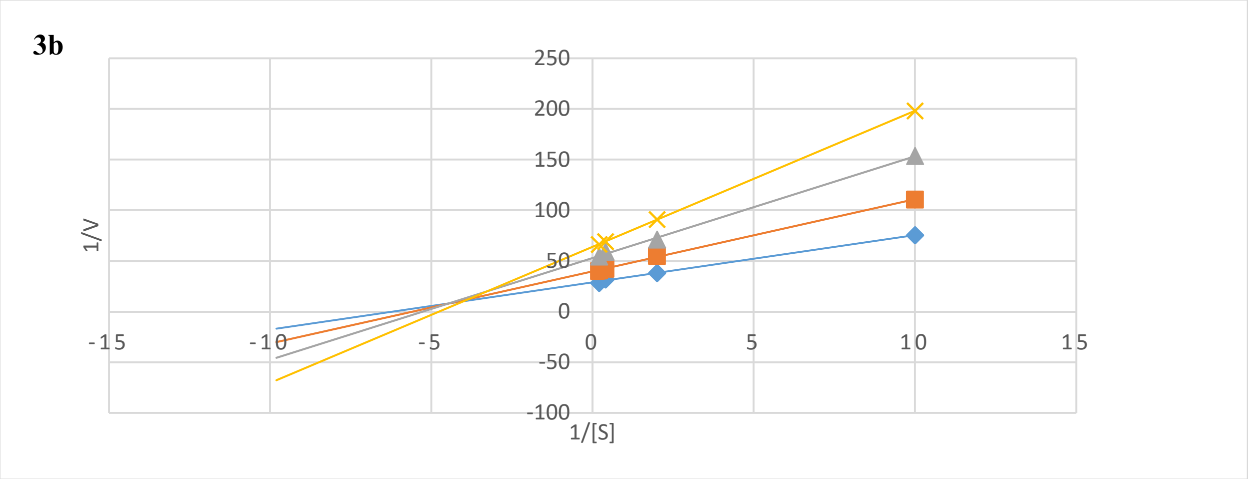

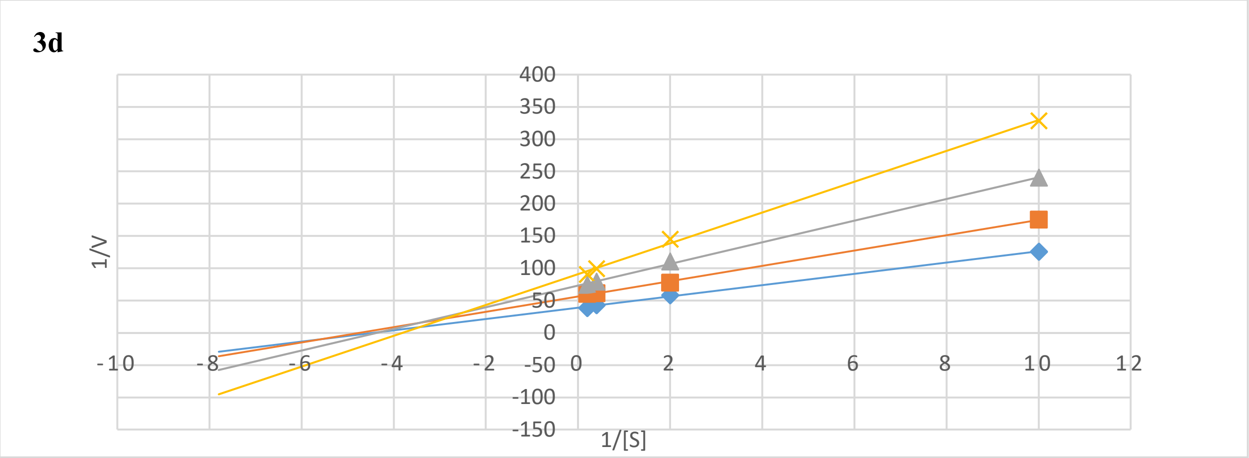

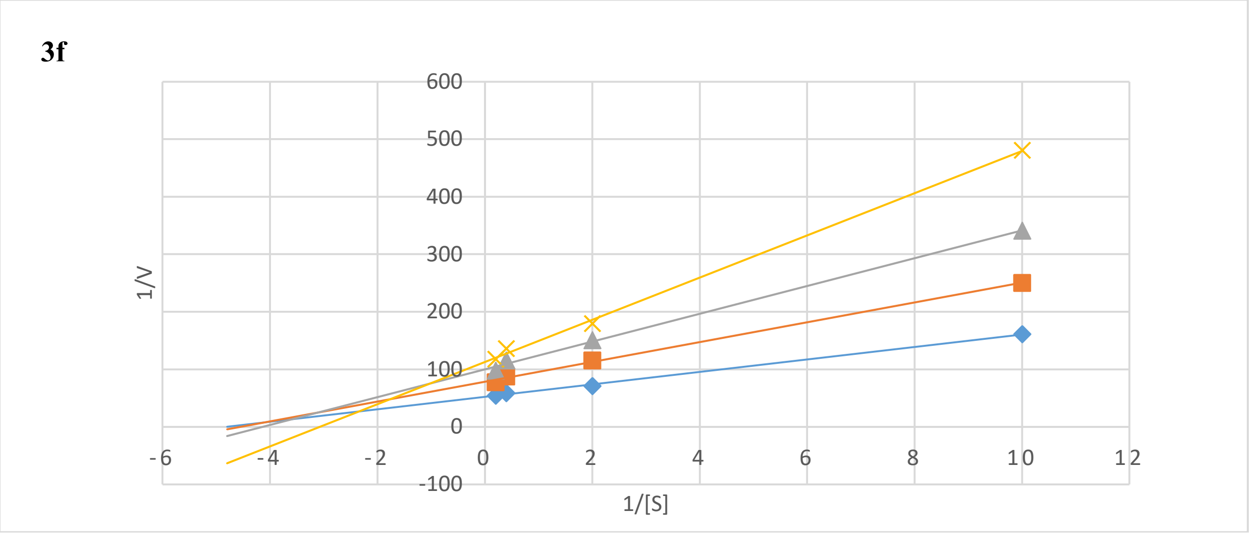

Figure S2.2.1: Lineweaver–Burk plots for inhibition of BChE by **2b**, **2f**, **3b**, **3d**, **3f**. Blue symbols and fitted straight lines represent enzyme activity in the absence of inhibitor, while orange (2.5 μM), grey (3.5 μM) and yellow (5 μM) represent various concentrations of inhibitor.

2b

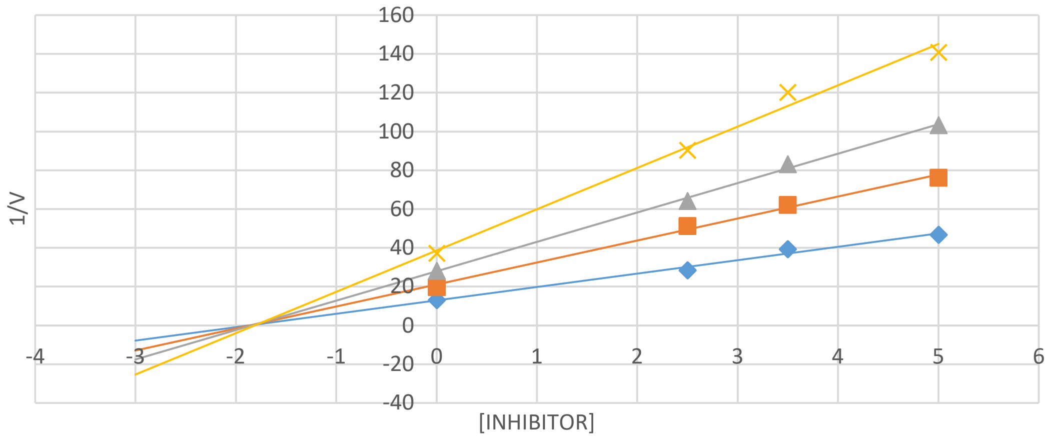
160
140
120
100
80
60
40
20
0
-20
-40
1/V
-4
-3
-2
-1
0
1
2
3
4
5
6
[INHIBITOR]

2f

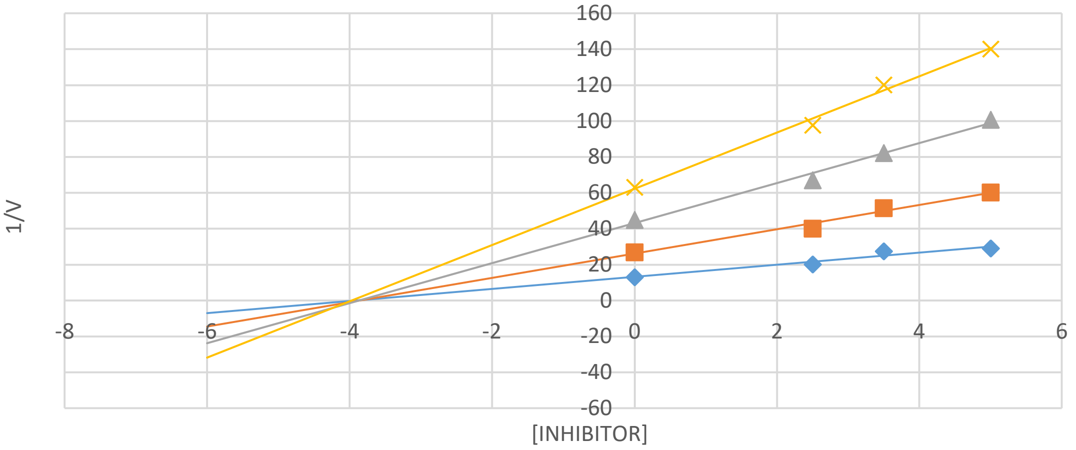
160
140
120
100
80
60
40
20
0
-20
-40
-60
1/V
-8
-6
-4
-2
0
2
4
6
[INHIBITOR]

3b

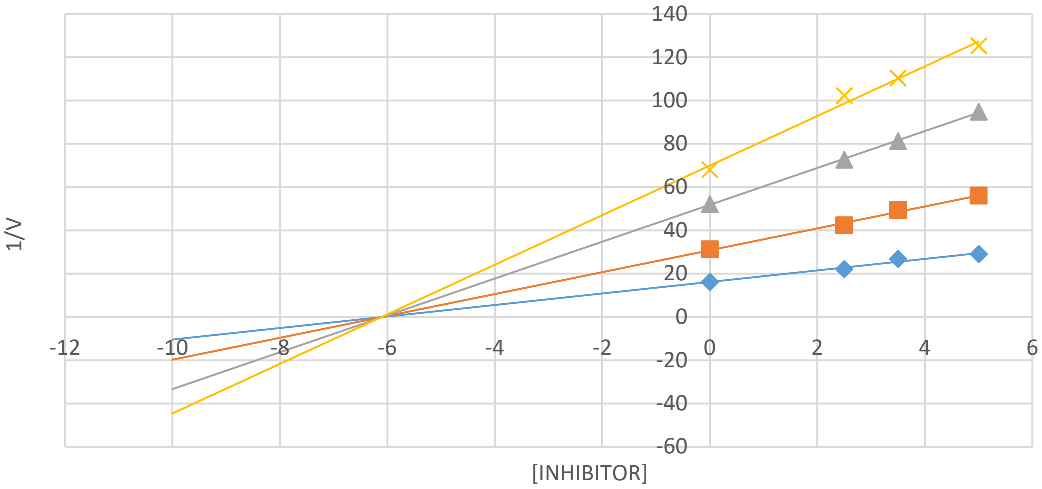
140
120
100
80
60
40
20
0
-20
-40
-60
1/V
-12
-10
-8
-6
-4
-2
0
2
4
6
[INHIBITOR]

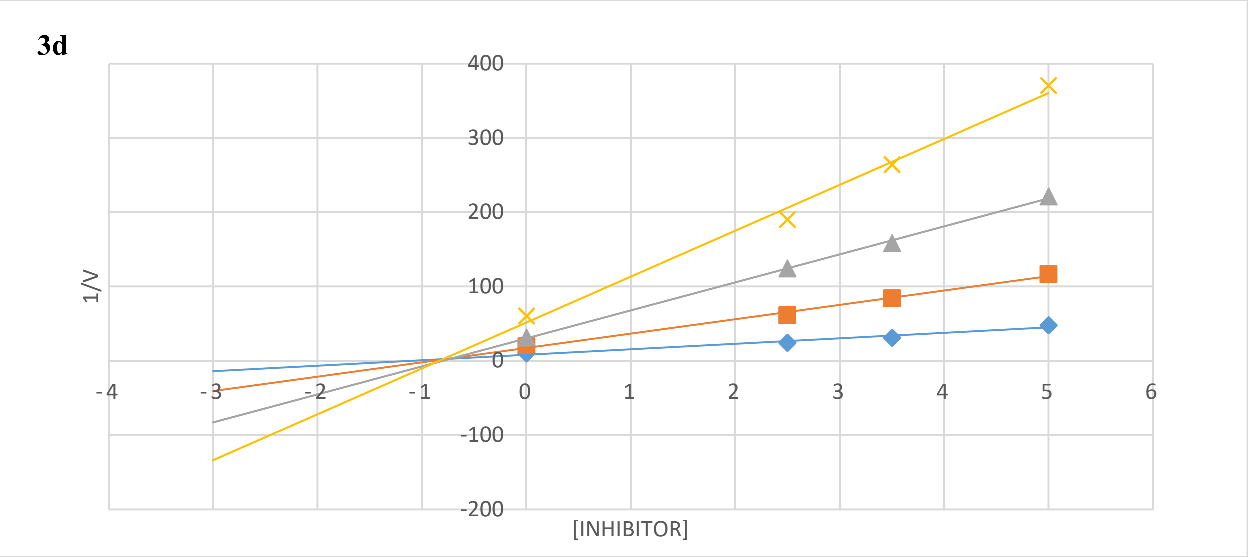

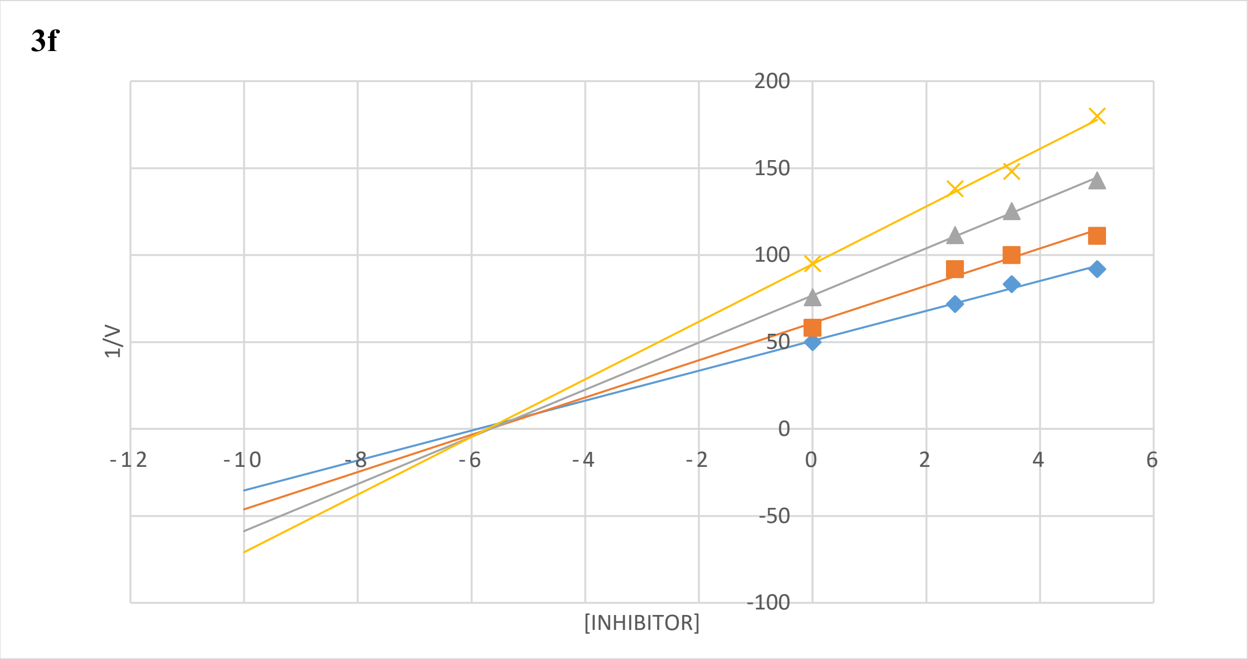

Figure S2.2.2: Dixon plots for inhibition of BChE by **2b**, **2f**, **3b**, **3d**, **3f**. Blue symbols and fitted straight lines represent enzyme activity with 5 mM substrate, while orange (2.5 mM), grey (0.5 mM) and yellow (0.1 mM) represent various concentrations of substrate.

Figure S2.3: Lineweaver-Burk and Dixon plots of 2f against BACE-1.

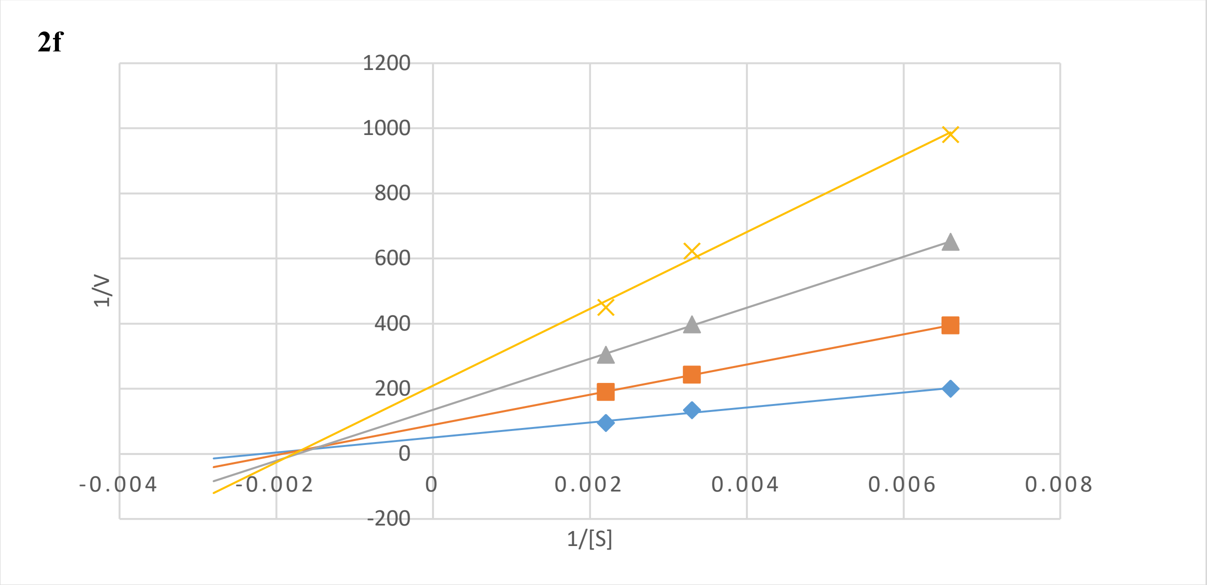

Figure S2.3.1: Lineweaver–Burk plots for inhibition of BACE-1 by **2f**. Blue symbols and fitted straight lines represent enzyme activity in the absence of inhibitor, while orange (2.5 µM), grey (3.5 µM) and yellow (5 µM) represent various concentrations of inhibitor.

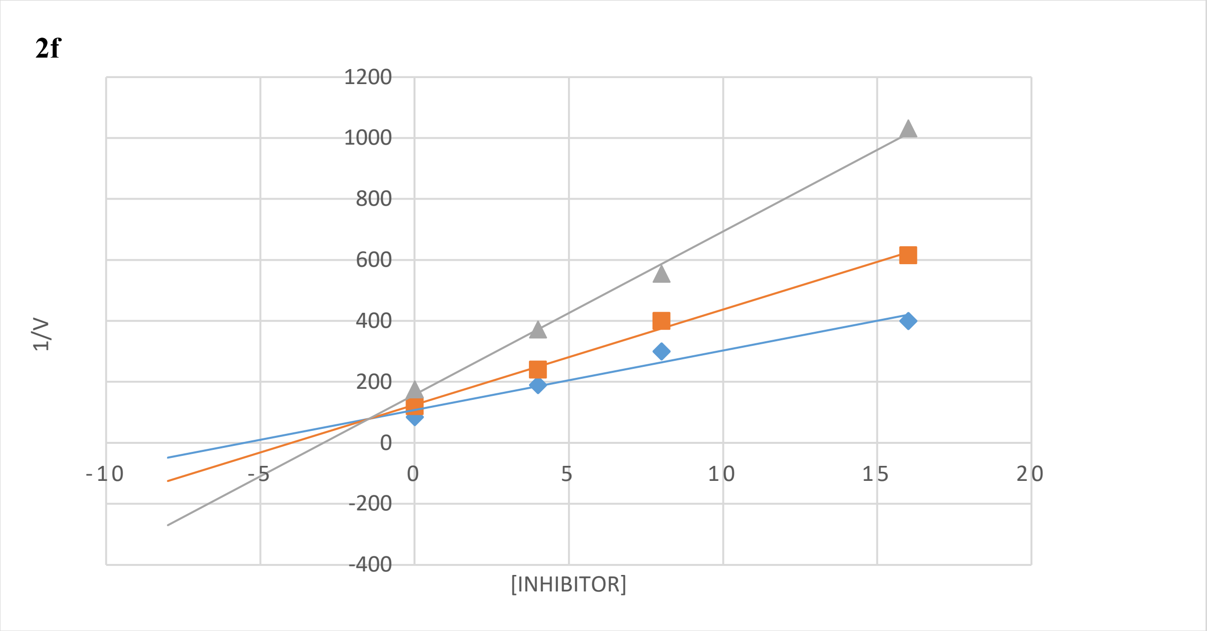

Figure S2.3.2: Dixon plots for inhibition of BACE-1 by **2f**. Blue symbols and fitted straight lines represent enzyme activity in the presence of 450 nM substrate and orange and grey, 300 nM and 150 nM substrate, respectively.

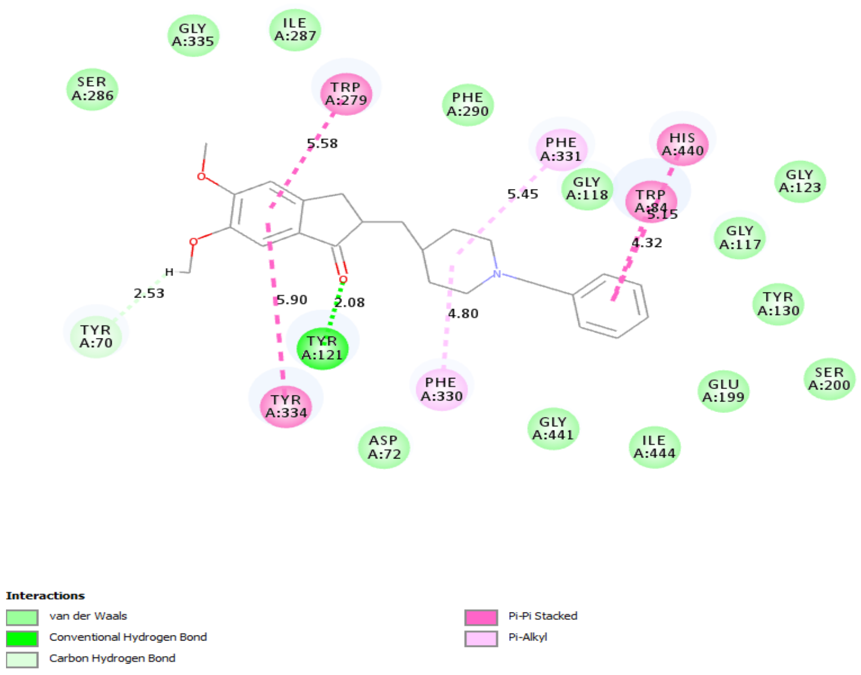

Figure S3: Docking pose of donepezil showing interaction with AChE protein residues.

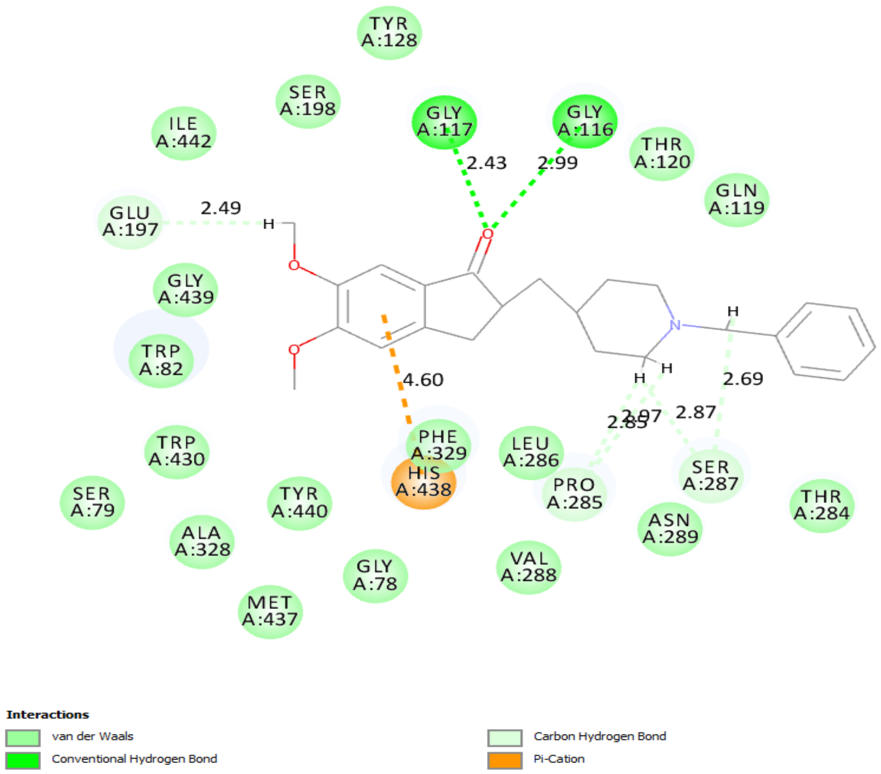

Figure S4: Docking pose of donepezil showing interaction with BChE protein residues.

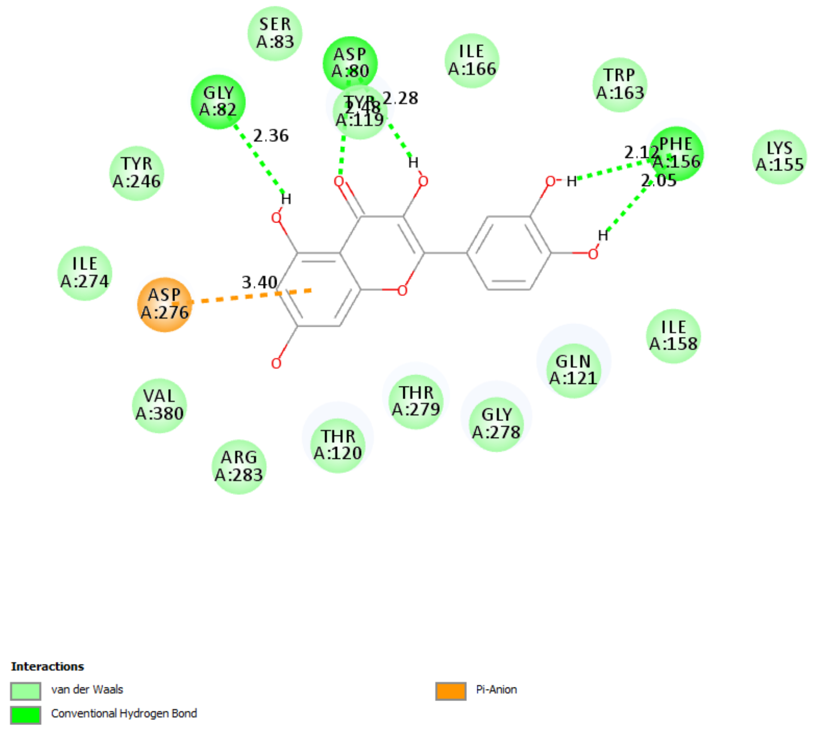

Figure S5: **Docking pose of quercetin showing interaction with BACE-1 protein residues.**

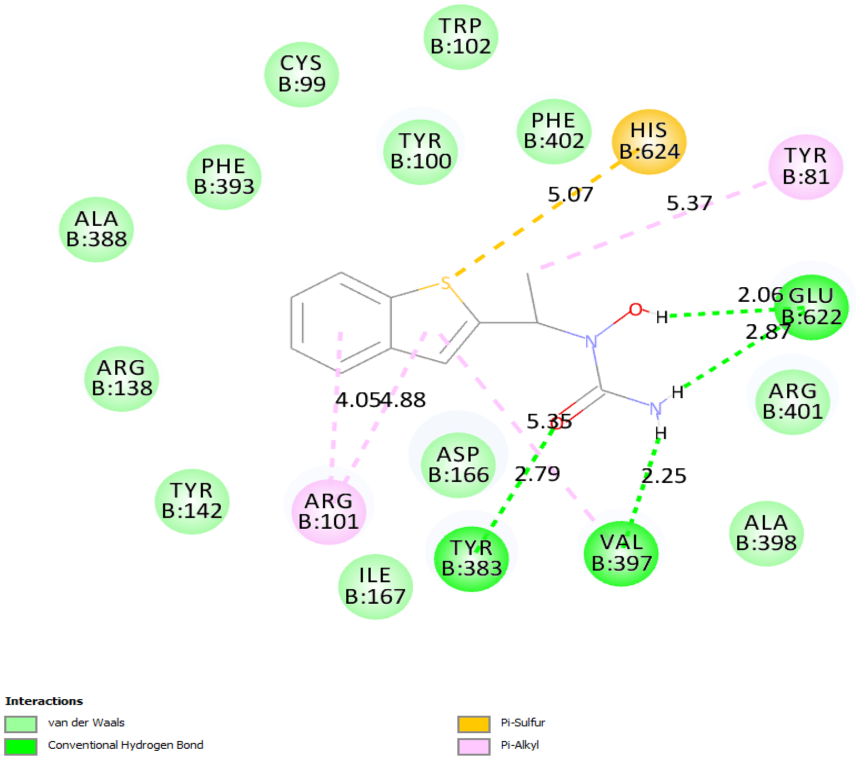

Figure S6: **Docking pose of zileuton showing interaction with LOX-5 protein residues.**

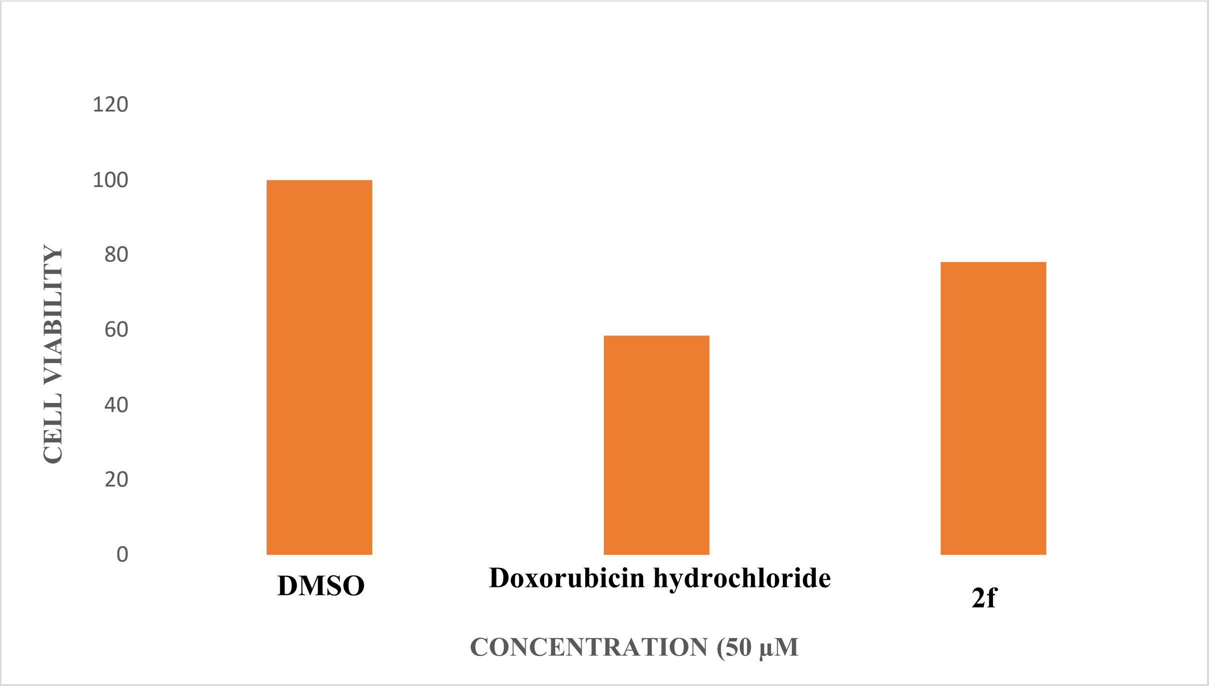

Figure S7: Evaluation of toxicity of **2f** in Hek293-T.
